# Investigating the molecular mechanism of traditional Chinese medicine for the treatment of placental syndromes by influencing inflammatory cytokines using the Mendelian randomization and molecular docking technology

**DOI:** 10.3389/fendo.2023.1290766

**Published:** 2024-02-01

**Authors:** Shan Huang, Shuangming Cai, Lin Ling, Wenni Zhang, Huanshun Xiao, Danfeng Yu, Xuan Zhong, Pei Tao, Yiping Luo

**Affiliations:** ^1^ Medical Intensive Care Unit, Guangdong Women and Children Hospital, Guangzhou, Guangdong, China; ^2^ Department of Rehabilitation, The Fifth Affiliated Hospital of Guangzhou Medical University, Guangzhou, Guangdong, China

**Keywords:** placental syndromes, inflammatory cytokines, molecular docking, traditional Chinese medicine, Mendelian randomization

## Abstract

**Introduction:**

Placental syndromes, which include pregnancy loss, preterm birth, gestational diabetes mellitus (GDM), and hypertensive disorders in pregnancy (HDP), have a strong association with disorder inflammatory reactions. Nonetheless, the exact causal relationship has not been established. This study aims to investigate the causal relationship between placental syndromes and inflammatory cytokines utilizing Mendelian randomization (MR). Additionally, we examined the interaction between small molecular compounds derived from traditional Chinese medicine and inflammatory cytokines using molecular docking method.

**Methods:**

After obtaining the data of inflammatory cytokines and placental syndromes, as well as establishing single nucleotide polymorphisms (SNPs), we employed the inverse variance weighted (IVW) method to assess the causal relationship. We also accessed the heterogeneity and the horizontal pleiotropy of these data. The “ClusterProfiler” R package was utilized for Kyoto Encyclopedia of Genes and Genomes (KEGG) pathway and Gene Ontology (GO) term analyses. The protein-protein interaction (PPI) network was constructed using STRING database. AutoDock Vina software was used for molecular docking, and Discovery Studio 2019 was used for visualization purposes.

**Results:**

We found that the growth regulated oncogene A (GROA) and interleukin-9 (IL-9) were associated with the development of pregnancy hypertension, whereas interleukin-10 (IL-10) and hepatocyte growth factor (HGF) were linked to the occurrence of preeclampsia. Moreover, there were correlations observed between interleukin-18 (IL-18), IL-10, macrophage colony-stimulating factor (MCSF), and platelet-derived growth factor BB (PDGFbb) in cases of chronic hypertension combined with pregnancy (CHP). Additionally, macrophage migration inhibitory factor (MIF) exhibited a connection with GDM, and TNF related apoptosis inducing ligand (TRAIL) demonstrated a causal relationship with preterm birth. It is plausible to suggest that interleukin-1β (IL-1β) might contribute to the promotion of pregnancy loss. All of the binding free energy values of small molecular compounds with inflammatory cytokines were below −5.0 kcal/mol. Furthermore, all of the RMSD values were less than 2.

**Conclusions:**

GROA, IL-1β, IL-9, IL-10, IL-18, MIF, MCSF, HGF, PDGFbb and TRAIL were found to be causally associated with placental syndromes. Molecular docking analysis revealed that small molecular compounds, such as puerarin, magnolol, atractylenolide I, paeoniflorin, tumulosic acid and wogonin, are closely bound to these inflammatory cytokines.

## 1 Introduction

During the pregnancy period, proper placentation plays an important role in allowing the development and maintenance of pregnancy (1). On the other hand, inadequate placentation may potentially contribute to the manifestation of various pregnancy-related pathologies, encompassing pregnancy loss, preterm birth, gestational diabetes mellitus (GDM), and hypertensive disorders in pregnancy (HDP) (2). The HDP includes a range of classifications, such as pregnancy hypertension, preeclampsia(PE)/eclampsia, and chronic hypertension combined with pregnancy (CHP) (3). In a 2019 article by Anne Cathrine Staff, these diseases were referred to as “placental syndromes” due to their common characteristic of impaired trophoblast invasion or trophoblast stress (4).

Placental syndromes are common diseases during pregnancy. The global incidence of PE ranges from 2% to 8% (5). The global incidence of CHP is estimated to be 0.9% to 1.5%, and from 2000 to 2009, there was a 67% increase in the incidence of CHP (6). The probability of experiencing a single pregnancy loss among women of reproductive age is 10%, while the probability of recurrent miscarriage is 1% to 5% (7). The incidence of preterm birth is approximately 10% (8). GDM, a major global public health issue, affects approximately 5.8% to 12.9% of pregnancies (9). These syndromes present a significant risk to the health of both the mother and fetus, resulting in an increasing strain on global healthcare systems (10). Therefore, identifying the pathophysiological causes of placental syndromes and searching for therapeutic drugs for etiological treatment are needed.

The pathological process of placental syndromes is not fully understood, and its association with inflammatory response has been suggested but not yet confirmed (11). The presence of a generalized inflammatory process can potentially result in the shallow invasion of trophoblasts or induce uterine contractions, subsequently leading to the development of HDP, preterm birth, pregnancy loss, and GDM (12). Despite several observational investigations aiming to elucidate the links between inflammatory cytokines and placental syndromes, the interpretation of their findings may be influenced by unforeseen confounders or reverse causality, thereby impeding the establishment of definitive causal relationships (13).

Mendelian randomization (MR) analysis is an innovative and potent epidemiological technique that utilizes genetic variants as unbiased instrumental variables to explore the causal associations between exposures and clinical manifestations of illnesses. MR has the potential to mitigate the impact of confounding variables and reverse causation, thereby enhancing the quality of evidence for causal inference (14). We extracted the relevant valid variations of 41 inflammatory cytokines from the genome-wide association study (GWAS) and used two-sample MR methods to investigate the causal effects of inflammatory cytokines on placental syndromes for further clarification of the psychophysiological causes.

As for searching therapeutic targets for placental syndromes, inflammatory factors, as systemic circulating proteins, are susceptible to the influence of small molecular compounds or biologics, making them ideal drug targets (15). Small molecules, such as puerarin, magnolol, atractylenolide I, paeoniflorin, tumulosic acid and wogonin, are traditional Chinese medicine compounds with anti-inflammatory effects. They have been widely used in the treatment of diseases such as hypertension, but their pharmacological mechanism on placental syndromes is still unclear (16). Molecular docking is a method used to study the interaction between compounds and targets to clarify the pharmacological mechanism (17). Therefore, in this study, we performed molecular docking to investigate the interaction between these small molecular compounds and inflammatory factors, thereby demonstrating their potential as therapeutic targets for placental syndromes through their interaction with inflammatory factors.

## 2 Methods

### 2.1 Study design

In order to estimate the causal relationship between inflammatory cytokines and placental syndromes, we conducted a two-sample MR study. We also used single nucleotide polymorphisms (SNPs) as instrumental variables (IV), which must satisfy three assumptions: (i) They must exhibit a strong association with the exposure; (ii) They must be independent of confounders that influence the association between the exposure and outcome; (iii) They must only show a relationship with the outcome though the exposure.

**Figure 1:**
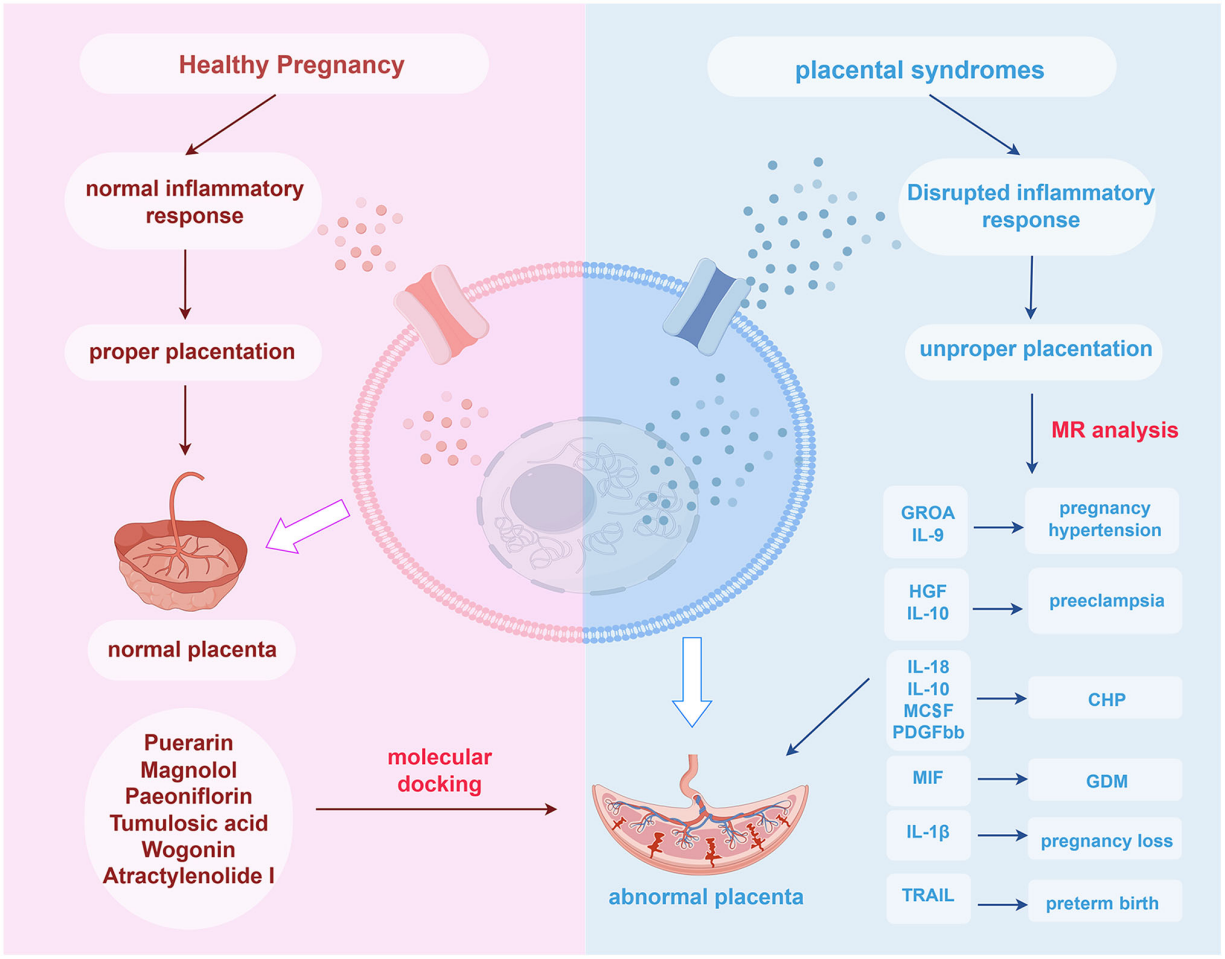
Overview of the research. GROA, growth-regulated oncogene-A; IL, interleukin; HGF, hepatocyte growth factor; MCSF, macrophage colony-stimulating factor; PDGFbb, platelet-derived growth factor BB; MIF, macrophage migration inhibitory factor; TRAIL, TNF-related apoptosis-inducing ligand; CHP, chronic hypertension combined with pregnancy; GDM, gestational diabetes mellitus; MR, Mendelian randomization.

After establishing a causal relationship between inflammatory cytokines and placental syndromes, we conducted gene ontology (GO) and Kyoto Encyclopedia of Genes and Genomes (KEGG) enrichment analyses, as well as protein-protein interaction (PPI) network, to gain a deeper understanding of their effect on diseases. Finally, we performed molecular docking studies of these inflammatory cytokines with small molecular compounds of traditional Chinese medicine with anti-inflammatory effects to investigate their drug targets for placental syndromes (**Figure 1**).

### 2.2 GWAS data for human inflammatory cytokines

The data of inflammatory cytokines were obtained from GWAS conducted by Ari V. Ahola-Olli et al. (18). This study examined the circulating concentrations of 41 inflammatory cytokines in a cohort of 8,293 Finnish individuals from three distinct population cohorts: FINRISK2002, FINRISK1997, and the Cardiovascular Risk in Young Finns Study (YFS), and is available at “https://www.ebi.ac.uk/gwas/downloads/summary-statistics”.

### 2.3 GWAS data for placental syndromes

The publicly available GWAS data for placental syndromes were derived from UK Biobank (UKB) or FinnGen, which can be accessed at “https://gwas.mrcieu.ac.uk”. The diseases associated with placental syndromes in our study include pregnancy hypertension (7686 cases, 115893 controls), PE (1864 cases, 461069 controls), CHP (1109 cases, 114735 controls), GDM (6033 cases, 110330 controls), pregnancy loss (34239 cases, 89340 controls), and preterm birth (5480 cases, 98626 controls). To ensure the reliability of these data, careful consideration was given to avoid any overlap in the selection of populations between the outcome groups and the exposure groups.

### 2.4 Instrumental variable selection

Firstly, the significant threshold for the whole genome was set at a *P*-value of less than 5*10^-6^ to select strongly associated SNPs with placental syndromes and inflammatory cytokines. This threshold was chosen because when set at *P*<5*10^-8^, only a few SNPs were selected. These selected SNPs were then clumped to avoid linkage disequilibrium. Next, R^2^ of each SNP was used to evaluate the proportion of variance in the exposure. Finally, F-statistic was used to estimate the instrument strength to avoid the weak instrument bias. And exposures with three or more valid SNPs were chosen in these study.

### 2.5 Two-sample MR analysis

As the inverse variance weighted (IVW) method weights the associations between SNPs and outcomes by inverse variance, it is an efficient approach to estimate the causal associations between exposures and outcomes. So we used the IVW method to evaluated the causal association between placental syndromes (outcomes) and inflammatory cytokines (exposures). Mendelian Randomization-Egger regression method (MR-Egger regression method) and weighted median method (WM method) were used as complementary method to estimate the association. Furthermore, to assess the influence of individual SNPs, a leave-one-out sensitivity analysis was conducted.

We also performed the Cochran’s Q statistic for MR-inverse-variance weighted and MR Egger analyses to test for heterogeneity. The MR-Egger regression method was used to test for horizontal pleiotropy.

The “TwoSampleMR” and “MendelianRandomization” R packages were employed to estimate these statistical methods. *P*<0.05 was considerate as statistical significance.

### 2.6 GO, KEGG enrichment and PPI network

To analyze the function of placental syndromes associated inflammatory cytokines, we performed KEGG pathway and GO term analyses using the “ClusterProfiler” package in R. Statistical significance was defined as *P* < 0.05.

To further assess protein network of the placental syndromes associated inflammatory cytokines, we utilized the STRING database (http://string-db.org/cgi/input.pl) to construct a PPI network. Subsequently, we employed the Cytoscape software to identify the core targets and evaluate their accuracy.

### 2.7 Molecular docking

To investigate the interaction between small molecular compounds of traditional Chinese medicine with inflammatory cytokines, we conducted molecular docking analysis. The protein crystal structures of these inflammatory cytokines were obtained from the Research Collaboratory for Structural Bioinformatics (RCSB) Protein Data Bank (http://www.pdb.org/). The structures were prepared by removing ligands and water molecules, adding hydrogen atoms, calculating charges, and optimizing amino acids using Autodock 1.5.6 software. The structural files of these small molecular compounds were obtained from The Traditional Chinese Medicine Systems Pharmacology Database and Analysis Platform (TCMSP), and their energy was minimized using Chem3D software. The protein and compound files were converted to PDBQT format, and molecular docking was performed using AutoDock Vina. Visualization of the results was done using Discovery Studio 2019.

## 3 Results

### 3.1 Selection of instruments

When the *P*-value of genome-wide significance was set to 5 × 10^-6^, the number of selected SNPs varied from 0 to 22. For the outcome of PE and the exposure of interleukin-9 (IL-9), no further analysis was conducted as it was not possible to extract SNPs from the outcome. For the outcome of PE and the exposures of interleukin-1β (IL-1β), macrophage colony-stimulating factor (MCSF), and tumor necrosis factor alpha (TNF-α), only 1 SNP was extracted, and the above results were excluded. For the outcome of PE and the exposures of cutaneous T cell-attracting chemokine (CTACK), basic fibroblast growth factor (FGF-Basic), growth-regulated oncogene-A (GROA), interleukin-5 (IL-5), interleukin-16 (IL-16), interleukin-17 (IL-17), monokine induced by interferon gamma (MIG), macrophage inflammatory protein-1A (MIP-1A), and tumor necrosis factor beta (TNF-β), 2 SNPs were extracted from the outcome, and the above results were also excluded. All the remaining SNPs were above 3, and all of the F-statistic values of these remaining SNPs were above 10, suggesting a low likelihood of weak instrumental bias (**Supplementary Table 3**).

### 3.2 The results of MR analysis

After selected the instruments, we used them to analyze the causal inference of inflammatory cytokines and placental syndromes. The IVW methodology was selected as the primary analysis method.

**Figure 2:**
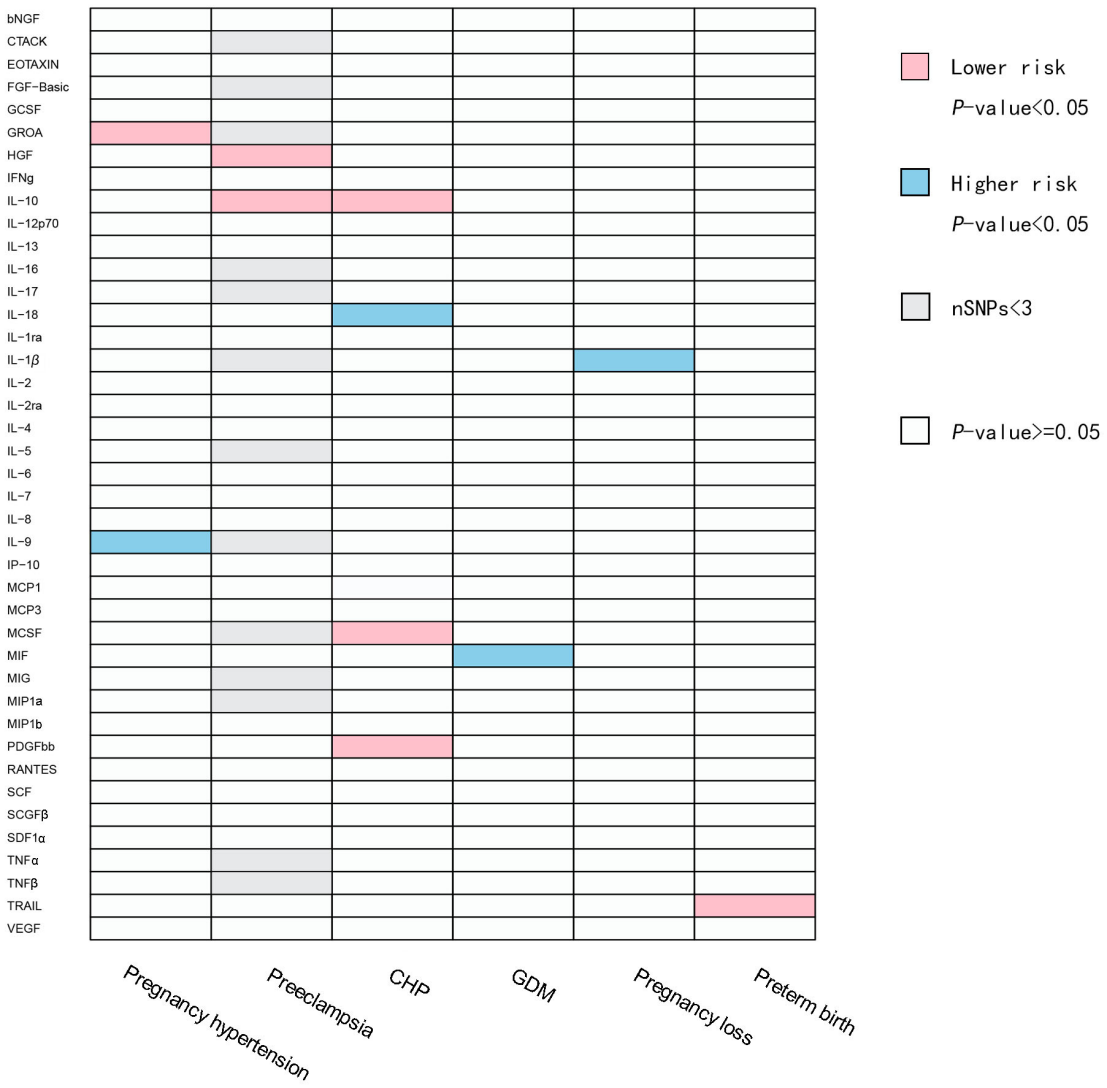
Causal correlations of inflammatory cytokines on placental syndromes. The pink squares represent inflammatory factors with protective effects on placental syndromes (OR<1). The blue squares represent inflammatory factors with promoting effects on placental syndromes (OR>1). The gray squares represent SNPs of the related inflammatory factors that are less than 3, and subsequent MR analysis cannot be performed. bNGF, beta nerve growth factor; CTACK, cutaneous T cell-attracting chemokine; FGF-Basic, basic fibroblast growth factor; GCSF, granulocyte colony-stimulating factor; GROA, growth-regulated oncogene-A; HGF, hepatocyte growth factor; IFNg, interferon gamma; IL, interleukin; IP, interferon gamma-induced protein 10; MCP1, monocyte chemotactic protein 1; MCP3, monocyte-specific chemokine 3; MCSF, macrophage colony-stimulating factor; MIF, macrophage migration inhibitory factor; MIG, monokine induced by interferon gamma; MIP1a, macrophage inflammatory protein-1a; MIP1b, macrophage inflammatory protein-1b; PDGFbb, platelet-derived growth factor BB; RANTES, regulated upon activation normal T cell expressed and secreted factor; SCF, stem cell factor; SCGFβ, stem cell growth factor beta; SDF1α, stromal cellderived factor-1 alpha; TNFα, tumor necrosis factor alpha; TNFβ, tumor necrosis factor beta; TRAIL, TNF-related apoptosis-inducing ligand; VEGF, vascular endothelial growth factor; CHP, chronic hypertension combined with pregnancy; GDM, gestational diabetes mellitus.

In the positive results, heterogeneity may exist when the exposure is IL-9 and the outcome is pregnancy hypertension, as indicated by the Cochran’s Q statistical analysis of MR Egger and IVW with the *P*-value less than 0.05. No heterogeneity was observed in the remaining positive results. Additionally, in the positive results, there is pleiotropy when the exposure is TNF-related apoptosis-inducing ligand (TRAIL) and the outcome is preterm birth. However, when the SNP rs13115587 is removed, the *P*-value of MR Egger is greater than 0.05, indicating no pleiotropy. For the negative results, pleiotropy is observed when the exposure is interleukin-13 (IL-13) and the outcome is preterm birth. However, even when we remove each individual SNP, the results still exhibit pleiotropy. When the exposure is interferon gamma (IFNg) and the outcome is pregnancy loss, pleiotropy is present, but after we removed the SNP rs115729819, the *P*-value of MR Egger is greater than 0.05, indicating no pleiotropy. All the remaining results showed no pleiotropy. No indications of weak instruments were found in any of the MR analyses in this study. A summary of the IVW test results are presented in **Figure 2**; **Supplementary Tables 1**, **2**, **Supplementary Figures 1**, **2**.

**Figure 3:**
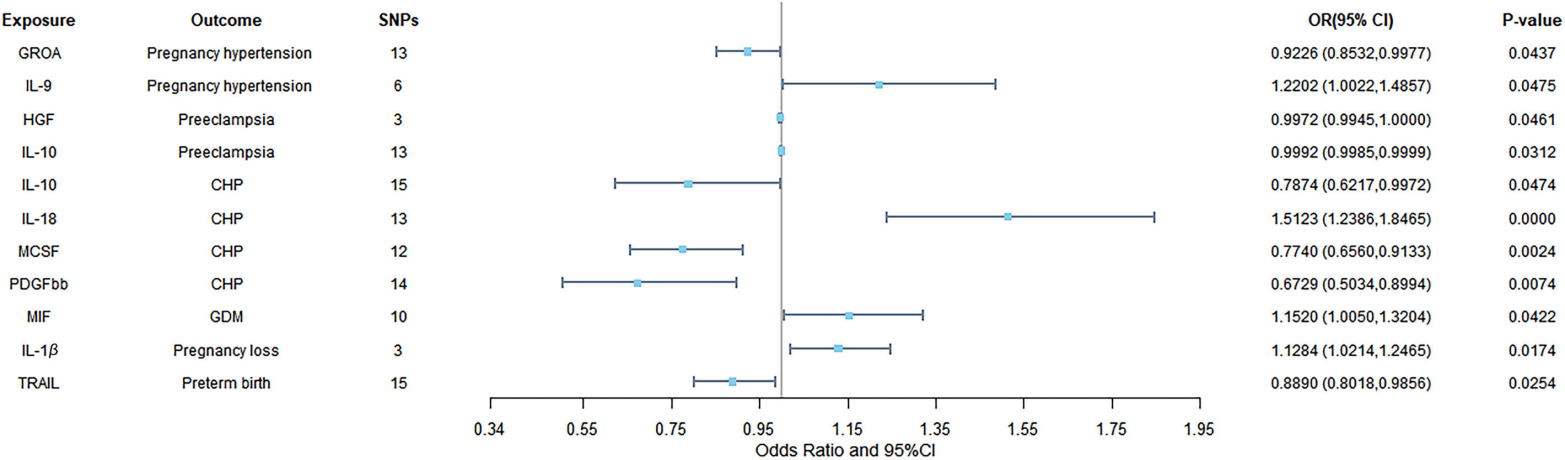
Causal correlations of inflammatory cytokines on placental syndromes. The odds ratio (OR) and the corresponding 95% confidence interval (CI) illustrate the change in the standard deviation (SD) of inflammatory cytokines with each incremental change in log odds related to placental syndromes. GROA, growth-regulated oncogene-A; IL, interleukin; HGF, hepatocyte growth factor; MCSF, macrophage colony stimulating factor; PDGFbb, platelet-derived growth factor BB; CHP, chronic hypertension combined with pregnancy; MIF, macrophage migration inhibitory factor; GDM, gestational diabetes mellitus; TRAIL, TNF-related apoptosis-inducing ligand.

A total of 11 significant associations, including 10 inflammatory cytokines were identified. We found that GROA and IL-9 exhibited causal relationships with pregnancy hypertension, while interleukin-10 (IL-10) and hepatocyte growth factor (HGF) demonstrated causal relationships with PE. Additionally, we observed correlations between interleukin-18 (IL-18), IL-10, MCSF, and platelet-derived growth factor BB (PDGF-bb) with CHP. Furthermore, macrophage migration inhibitory factor (MIF) displayed a relationship with GDM, and TRAIL showed a causal correlation with preterm birth. It is plausible that IL-1β may contribute to the promotion of pregnancy loss. **Figure 3** illustrates all relationships with a significance level of *P*<0.05 in IVW models.

**Figure 4:**
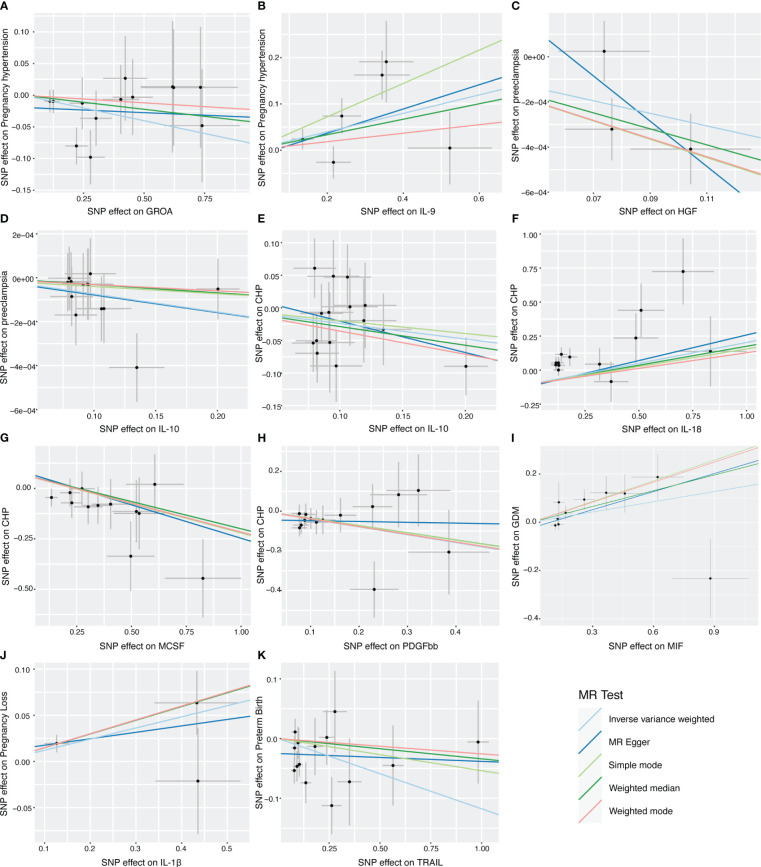
Scatter plots of MR analysis between inflammatory cytokines and the placental syndromes. **(A, B)** GROA and IL-9 on pregnancy hypertension. **(C, D)** HGF and IL-10 on preeclampsia. **(E–H)** IL-10, IL-18, MCSF and PDGFbb on CHP. **(I)** MIF on GDM. **(J)** IL-1β on pregnancy loss. **(K)** TRAIL on preterm birth. GROA, growth-regulated oncogene-A; IL, interleukin; HGF, hepatocyte growth factor; MCSF, macrophage colony-stimulating factor; PDGFbb, platelet-derived growth factor BB; CHP, chronic hypertension combined with pregnancy; MIF, macrophage migration inhibitory factor; GDM, gestational diabetes mellitus; TRAIL, TNF-related apoptosis-inducing ligand.

Hence, IL-1β, IL-9, IL-10, IL-18, HGF, GROA, MIF, MCSF, PDGF-bb and TRAIL may be considered as therapeutic targets for placental syndromes. **Figure 4** is the scatter plots of MR analysis.

### 3.3 GO, KEGG enrichment and PPI network

**Figure 5:**
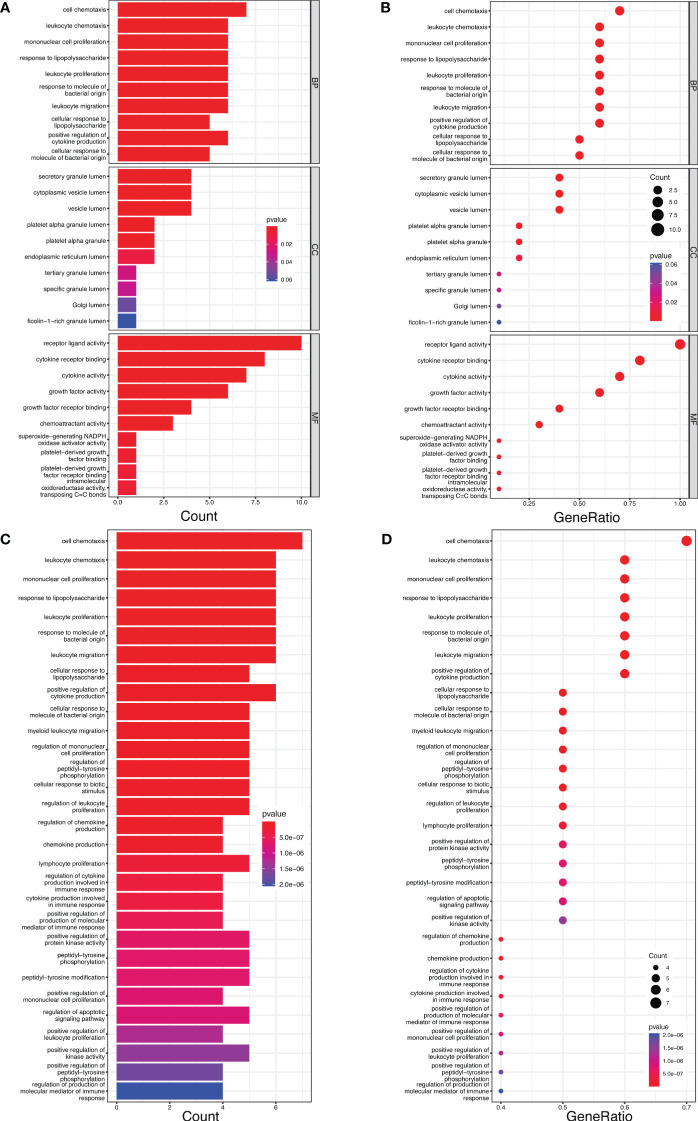
The bar and bubble plots of GO and KEGG analyses. **(A)** Bar plot of GO enrichment. **(B)** Bubble plot of Go enrichment. **(C)** Bar plot of KEGG enrichment. **(D)** Bubble plot of KEGG enrichment.

In GO enrichment analysis, the top five biological process terms were “cell chemotaxis”, “leukocyte chemotaxis”, “mononuclear cell proliferation”, “response to lipopolysaccharide”, and “leukocyte proliferation”. In terms of cellular components, the five most significantly enriched terms were “secretory granule lumen”, “cytoplasmic vesicle lumen”, “vesicle lumen”, “platelet alpha granule lumen” and “platelet alpha granule”. As for molecular functions, the top five enriched terms were “receptor ligand activity”, “cytokine receptor binding”, “cytokine activity”, “growth factor activity” and “growth factor receptor binding” (**Figures 5A, B**).

Furthermore, the KEGG pathway enrichment analysis revealed five enriched pathways: “cell chemotaxis”, “leukocyte chemotaxis”, “mononuclear cell proliferation”, “response to lipopolysaccharide” and “leukocyte proliferation” (**Figures 5C, D**).

**Figure 6:**
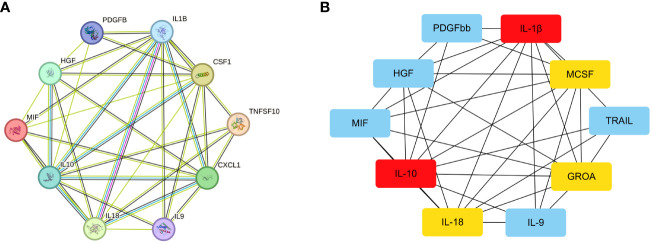
The PPI network for the inflammatory cytokines associated with placental syndromes. **(A)** PPI network for the inflammatory cytokines associated with placental syndromes. **(B)** The most significant top 5 inflammatory cytokines in the PPI network.

After uploading the inflammatory cytokines associated with placental syndromes to the STRING online database, we utilized the Cytoscape software to create a PPI network (**Figure 6**). Subsequently, we employed the CytoHubba software to identify the top 5 hub genes, which are IL-1β, IL-10, MCSF, IL-18 and GROA (**Figure 6**).

### 3.4 Molecular docking

Based on MR analysis, we can draw causal conclusions about disease development by examining changes in gene or protein expressions, which may serve as potential drug targets. Therefore, we specifically selected the small molecular compounds found in traditional Chinese medicine with anti-inflammatory effects, such as puerarin, magnolol, atractylenolide I, paeoniflorin, tumulosic acid and wogonin, as key active ingredients for molecular docking with inflammatory cytokines associated with placental syndromes. During the molecular docking, evaluation of binding capacity depends on the binding energy between ligands and receptors. The lower the binding energy, the more stable the conformation. Therefore, we set the binding energy to be lower than -5 kcal/mol, and all our results meet this criterion, suggesting that the key active components were well combined with the key targets.

**Figure 7:**
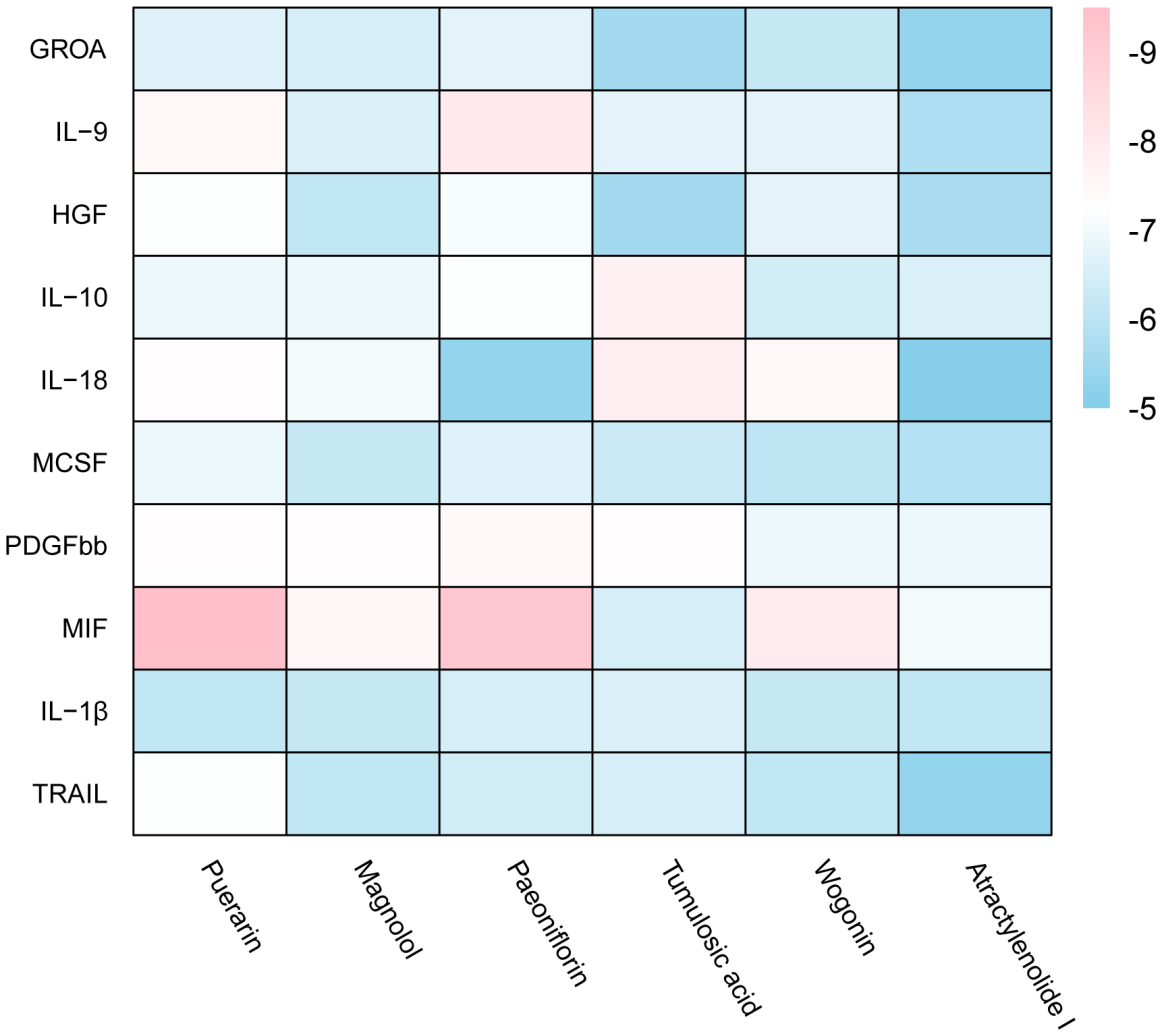
Heat map of estimated free binding energy of small molecular compounds and inflammatory cytokines.

**Figure 8:**
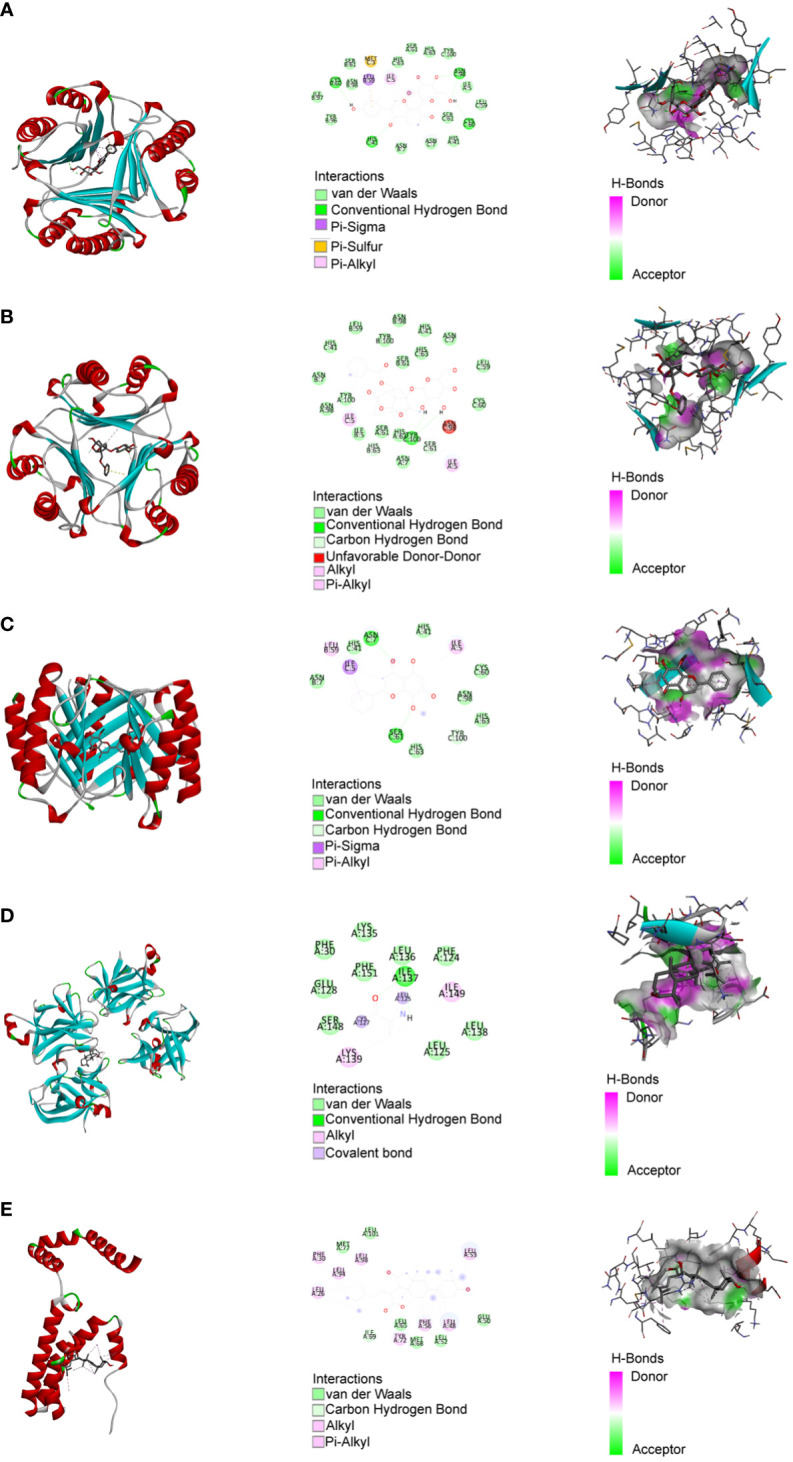
Molecular docking. **(A)** MIF with Puerarin; **(B)** MIF with Paeoniflorin; **(C)** MIF with Wogonin; **(D)** IL-18 with Tumulosic acid **(E)** IL-10 with Tumulosic acid.

Additionally, all of the root-mean-square deviation (RMSD) values in the molecular docking results were less than 2, indicating a strong interaction between these small molecular compounds and the inflammatory cytokines. These results suggest that these small molecular compounds could be suitable targets for the treatment of placental syndromes. These findings are illustrated in **Tables 1**, **2**, **Figures 7**, **8**.

**Table 1:** The basic information of small molecular compounds found in traditional Chinese medicine with anti-inflammatory factors.

Component	Molecular ID	chemical formula	molecular weight	Source	Structure
Puerarin	MOL012297	C_21_H_20_O_9_	416.4	Radix Puerariae	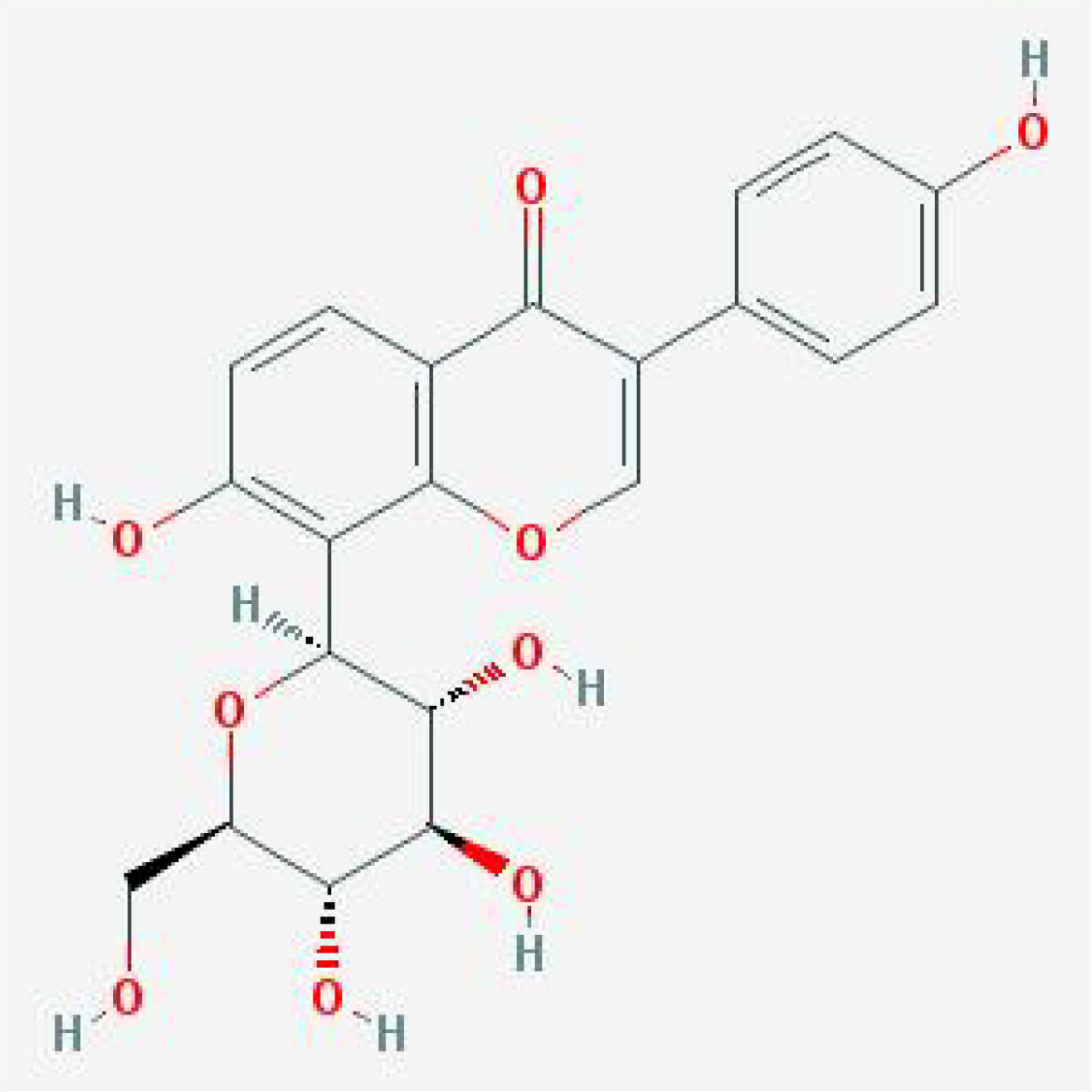
Magnolol	MOL000210	C_18_H_18_O_2_	266.3	Magnolia officinalis	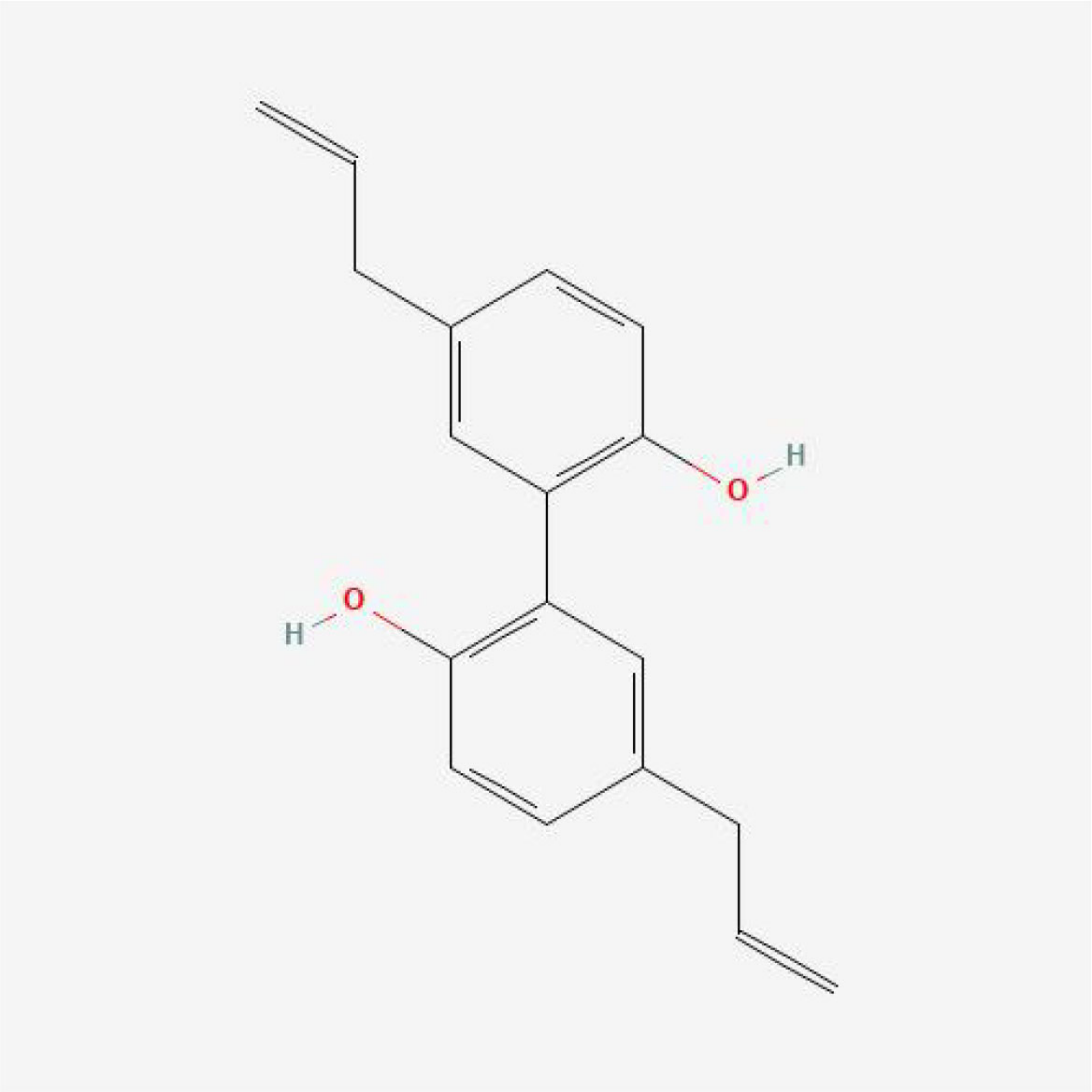
Paeoniflorin	MOL001924	C_23_H_28_O_11_	480.5	Radix Paeoniae Rubra	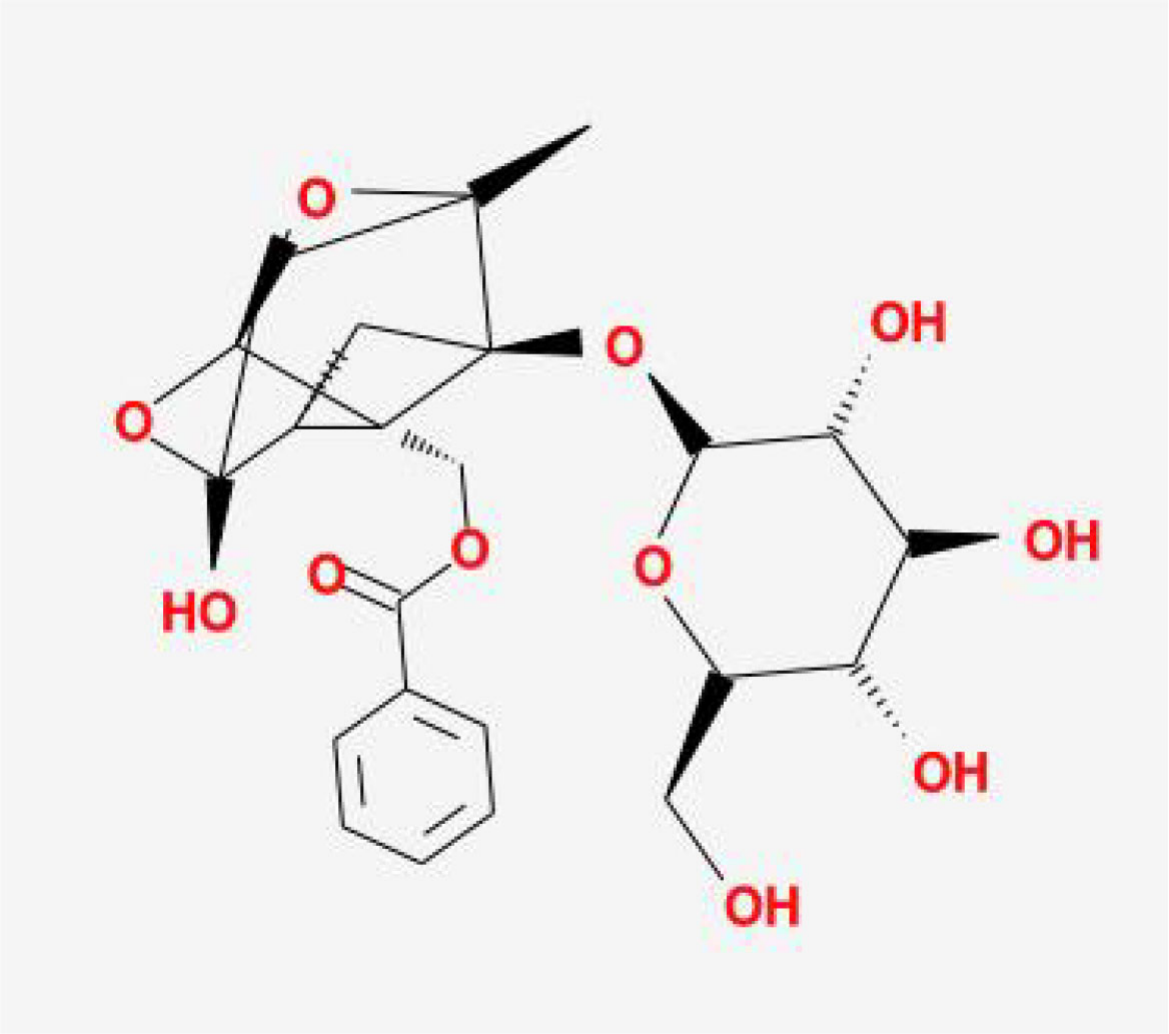
Tumulosic acid	MOL000277	C_31_H_50_O_4_	486.7	Poria cocos Wolf.	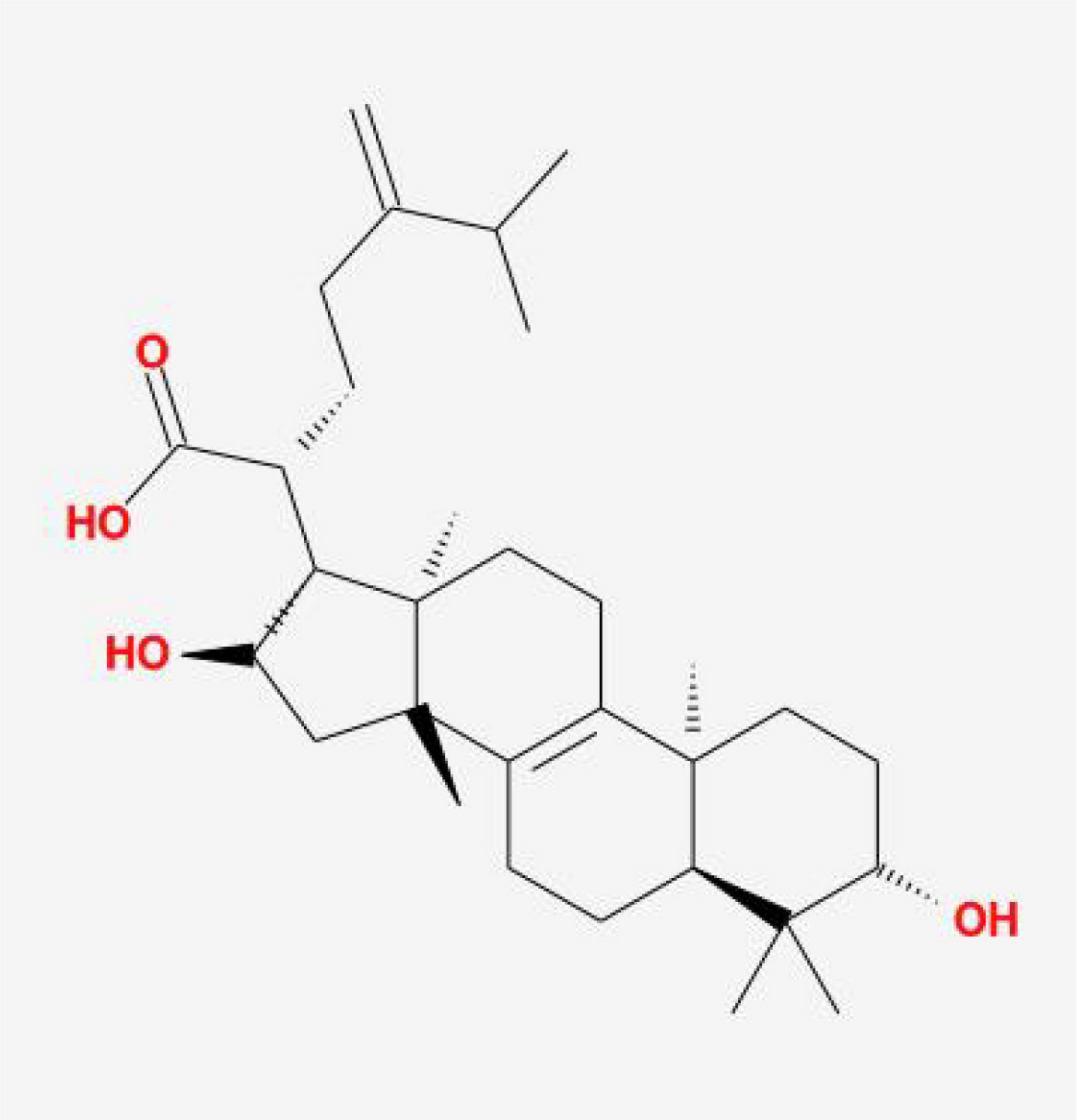
Wogonin	MOL000173	C_16_H_12_O_5_	284.28	Scutellariae Radix	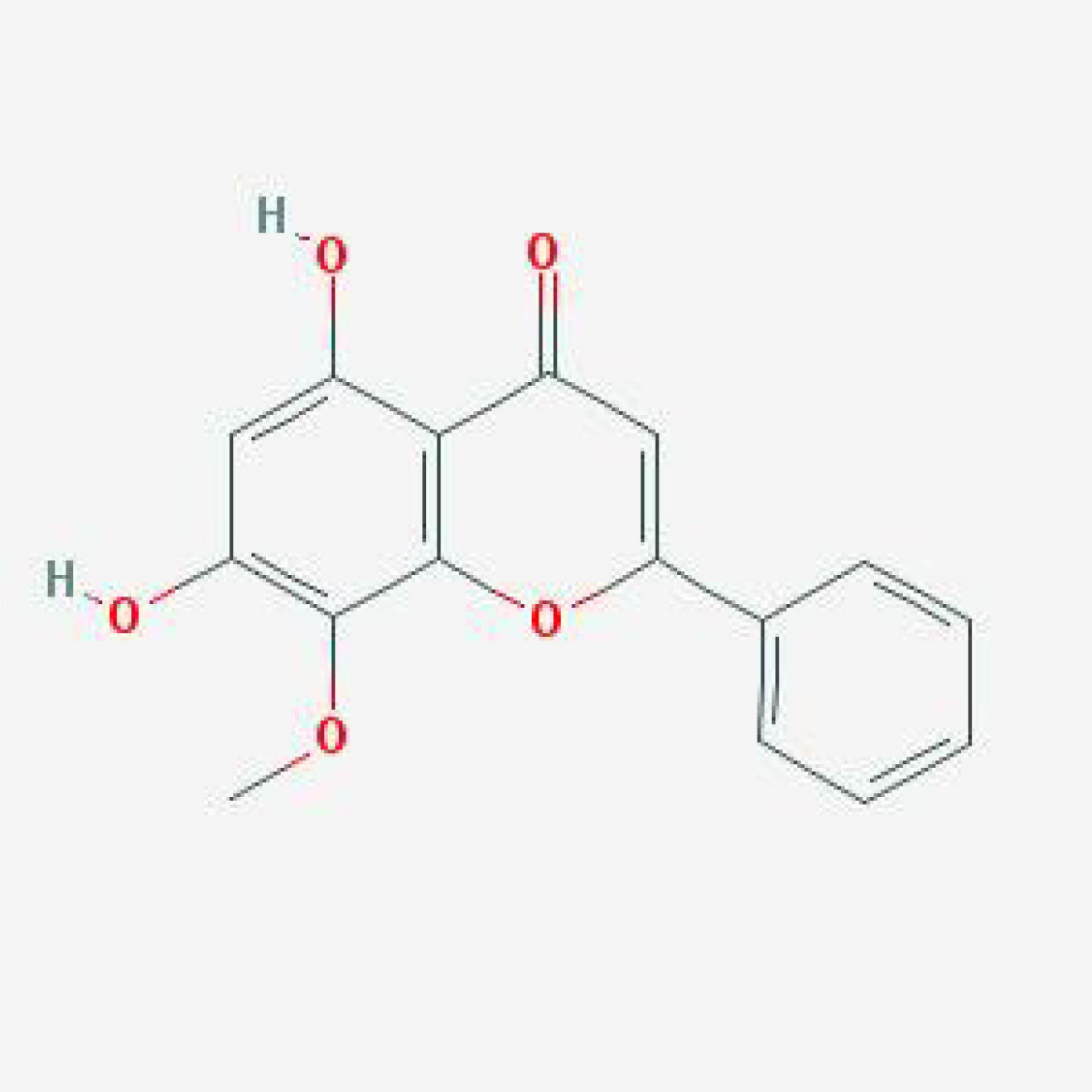
Atractylenolide I	MOL000043	C_15_H_18_O_2_	230.3	Atractylodes Macrocephala Koidz.	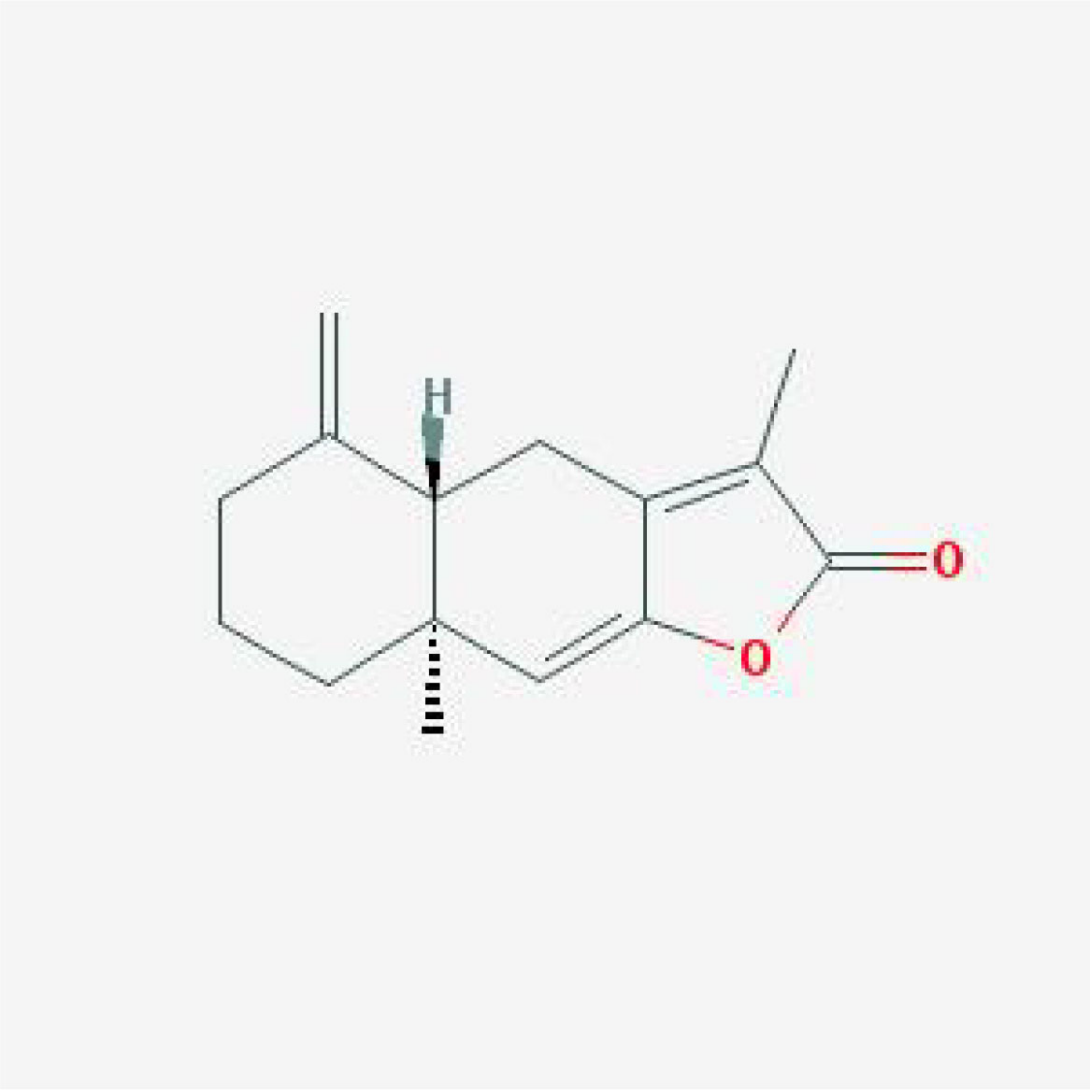

**Table 2:** Free binding energy of these small molecular compounds and inflammatory cytokines.

Small Molecular Compounds	Targets	RMSD	Free binding enegry(kcal/mol)
Puerarin	GROA	1.517	-6.7
	IL-9	1.614	-7.5
	HGF	1.813	-7.2
	IL-10	0.123	-6.9
	IL-18	1.774	-7.3
	MCSF	1.954	-6.9
	PDGFbb	1.688	-7.3
	MIF	1.827	-9.5
	IL-1β	0.702	-6.1
	TRAIL	1.648	-7.2
Magnolol	GROA	0.990	-6.5
	IL-9	0.025	-6.6
	HGF	0.929	-6.1
	IL-10	0.022	-6.9
	IL-18	0.349	-7.0
	MCSF	0.875	-6.2
	PDGFbb	0.024	-7.3
	MIF	0.066	-7.6
	IL-1β	0.350	-6.2
	TRAIL	1.157	-6.1
Paeoniflorin	GROA	1.787	-6.8
	IL-9	1.533	-8.0
	HGF	1.928	-7.1
	IL-10	1.687	-7.2
	IL-18	1.281	-5.3
	MCSF	1.736	-6.7
	PDGFbb	1.500	-7.5
	MIF	1.925	-9.2
	IL-1β	1.450	-6.5
	TRAIL	1.980	-6.4
Tumulosic acid	GROA	1.728	-5.6
	IL-9	1.979	-6.8
	HGF	1.919	-5.6
	IL-10	1.945	-7.7
	IL-18	1.544	-7.8
	MCSF	1.958	-6.3
	PDGFbb	0.810	-7.3
	MIF	1.774	-6.5
	IL-1β	1.741	-6.6
	TRAIL	1.403	-6.5
Wogonin	GROA	1.700	-6.2
	IL-9	1.226	-6.8
	HGF	1.117	-6.8
	IL-10	1.161	-6.4
	IL-18	1.066	-7.5
	MCSF	1.606	-6.0
	PDGFbb	1.369	-6.9
	MIF	1.435	-7.9
	IL-1β	1.396	-6.2
	TRAIL	1.398	-6.1
Atractylenolide I	GROA	1.790	-5.3
	IL-9	1.275	-5.8
	HGF	1.946	-5.7
	IL-10	1.550	-6.6
	IL-18	1.850	-5.0
	MCSF	1.831	-5.9
	PDGFbb	1.362	-6.9
	MIF	1.914	-7.0
	IL-1β	1.258	-6.1
	TRAIL	1.702	-5.3

## 4 Discussion

The underlying causes of placental syndromes are still not fully understood, and there is a lack of effective treatment method (19). Although there is a suggestion that inflammatory factors may be involved in the development of placental syndromes, there is currently insufficient evidence to establish a causal relationship (20).

MR method is often used to assess the causal relationship between exposure and outcome. Traditional statistical research methods such as randomized controlled trials (RCTs), while ensuring consistency in factors other than exposure, are often impractical due to economic and ethical constraints (21). Observational studies, in both control and intervention groups, inevitably exhibit differences in aspects other than exposure, making it difficult to establish direct causal relationships (22). The instrumental variable method has been proposed as an alternative statistical approach to control confounding factors and examine the causal relationship between exposure and outcome (23). In medical research, SNPs are increasingly being used as instrumental variables, as their alleles are assigned to individuals before any exposure occurs. This ensures comparability in all confounding factors, both known and unknown, allowing for the evaluation of the causal relationship between exposure and outcome. The term “Mendelian randomization” was coined due to its association with Mendel’s laws (24). In this study, we used MR method to demonstrate that an imbalance of inflammatory factors may contribute to the development of placental syndromes.

PE, one of the placental syndromes, is characterized by elevated blood pressure and organ damage during pregnancy. The pathogenesis of PE is closely associated with imbalance in inflammatory factors, which may lead to apoptosis of trophoblast, insufficient trophoblast invasion, and inadequate spiral artery remodeling (25).

IL-10, acting as an anti-inflammatory cytokine, drives the expression of anti-inflammatory mediators and suppressing the release of pro-inflammatory cytokines (26). The occurrence of PE is closely associated with inflammatory responses. IL-10 plays a role in inhibiting the release of inflammatory factors, and protecting against apoptosis of trophoblast caused by inflammatory factors, insufficient invasion of spiral arteries, and the occurrence of PE (27). Previous studies have investigated the link between IL-10 and PE. For instance, Kalkunte et al. identified a potential association between IL-10 deficiency and the development of PE (28). Additionally, Aggarwal observed a downregulation of IL-10 protein level in women with PE (29).

HGF plays a crucial role in the normal formation of the placenta. Li et al. observed that upon binding to its receptor Met, HGF can activate the downstream Erk pathway, facilitating trophoblast invasion and maintaining a healthy pregnancy. While hypoxia can disrupt the regulation of the HGF/Met signaling pathway, and promoting the occurrence of PE (30). Chaudhary et al. reported a downregulation of HGF in cases of PE (31). In our study, we also established a protective relationships between IL-10 and HGF with PE. These findings highlight the potential of early intervention therapies targeting IL-10 and HGF to prevent adverse pregnancy outcomes.

Pregnancy hypertension refers to elevated blood pressure that occurs during pregnancy without organ dysfunction. It represents a distinct stage of the same disease as PE and shares similar pathogenic mechanisms.

GROA is a member of the CXC chemokine family. It possesses the ability to activate leukocyte migration, enhance endothelial cell damage, regulate inflammation and angiogenesis (32). However, its specific function in the pathological process of pregnancy hypertension is still largely unknown because of the limited research. Due to the shared pathogenic mechanisms of pregnancy hypertension and PE, we conducted a search on the interaction between GROA and PE, and found that relevant studies are still scarce. Mellembakken JR et al. discovered elevated levels of GROA in PE patients, and found that GROA may activate inflammatory cells such as neutrophils and monocytes, inducing their accumulation in the placenta, leading to excessive inflammatory response, and ultimately resulting in spiral artery microcirculation obstruction, thus contributing to the development of PE (33). While, in our study, we found a protective relationship between GROA and pregnancy hypertension. This discrepancy could be attributed to the fact that MR studies are based on genetic-level investigations and may exclude certain confounding factors. Further research is necessary to evaluate the causal relationship between GROA and pregnancy hypertension.

The immunobiological functions of IL-9 are dual in nature, as it can both promote the development of certain diseases such as hypersensitivity reactions and inhibit the progression of others such as parasitic infections (34). Nevertheless, there is a scarcity of studies investigating the association between IL-9 and pregnancy hypertension. Further research is necessary to elucidate the roles of IL-9 in pregnancy hypertension.

CHP is also a subtype of HDP characterized by high blood pressure occurring before 20 weeks of gestation or persisting for more than 42 days after delivery. IL-18 is a pro-inflammatory cytokine. Some studies have observed an upregulation of IL-18 in HDP (35). Socha MW et al. found that during the progression of HDP, cholesterol, glucose, and uric acid activate the NLRP3 inflammasome, thereby promoting the generation of IL-18. This excessive inflammatory response triggers the apoptosis of trophoblast, ultimately contributing to the development of HDP. However, the research about the relationship of IL-18 and CHP are still rare. Our research revealed the promoting effect of IL-18 on CHP (36).

It has been suggested that MCSF plays a role in the regulation of survival, proliferation, and differentiation of macrophages and monocytes and contribute to the maintenance of normal pregnancy (37). The relationship between MCSF and CHP remains unknown. We observed a protective effect of MCSF on CHP through the MR analysis. Further researches is necessary to assess the impact of MCSF on CHP.

Additionally, PDGFbb has been shown to potentially contribute to the development of PE in certain studies (38). Since both CHP and PE fall under the category of HDP, the role of PDGFbb in CHP may be similar to that in PE. Nevertheless, our study, utilizing the MR method, suggests that PDGFbb has a protective effect on CHP, which may contradict existing research. This discrepancy could be attributed to the fact that MR studies are based on genetic-level investigations and may exclude certain confounding factors. Further research is necessary to elucidate the role of PDGFbb in CHP.

MIF is widely acknowledged as a pro-inflammatory cytokine that plays a crucial role in the innate immune response (39). Investigations into the relationship between MIF and GDM remain relatively scarce. Some researches have demonstrated that MIF may contribute to hyperglycemia by altering lymphocyte activity. MIF can markedly diminish pancreatic islet cell response to specific antigen stimulation and intercellular adhesion, while upregulating inflammatory mediators such as NO, IFN-γ, TNF-α, and IL-10, ultimately leading to elevated blood glucose levels (40). Y. Zhan et al. have confirmed the association between MIF and GDM (41). Yilmaz Ö. et al. have also observed an upregulation of MIF in patients with GDM (42). Consistent with these findings, our study further establishes a promotive causal relationship between MIF and GDM.

Pregnancy loss is a pathological manifestation encompassed within the spectrum of placental syndromes (43). IL-1β is known for a pro-inflammatory cytokines (44). However, the connection between pregnancy loss and IL-1β has not been thoroughly investigated (45). Immune balance is vital for the health of both the mother and the fetus, as any imbalance may lead to miscarriage. The intricate interplay between Th-1 (T-helper 1) and Th-2 (T-helper 2) cells is paramount for immune balance. If there is an imbalance between Th-1 and Th-2, with elevated levels of Th-1 cytokines, the risk of early pregnancy loss significantly increases. IL-1β can induce an elevation in Th-1 cells, which may be one of the causes of miscarriage induced by IL-1β (46). In this study, using the MR method, we present compelling evidence supporting the link between pregnancy loss and IL-1β, suggesting that IL-1β may play a potential role in promoting pregnancy loss.

TRAIL is a well-known factor that promotes cell apoptosis, and it exerts its effects by binding to its receptors TRAILR1 (47). The relationship between TRAIL and preterm birth is not well understood, and there is limited research on this topic. Some studies suggest that in early pregnancy, TRAIL promotes embryonic apoptosis (48). However, other studies propose that in the later stages of pregnancy, atrial natriuretic peptide (49) and PDGF (50) can promote angiogenesis, leading to the upregulation of TRAIL on trophoblast cells. This, in turn, induces lymphocyte and NK cell death in the placenta and protecting trophoblasts from attacks by lymphocyte and NK cells. Our research has discovered a protective causal relationship between TRAIL and preterm birth, but further research is needed to clarify the interaction between TRAIL and preterm birth.

After identifying the causal relationship between inflammatory cytokines and placental syndromes, we performed molecular docking of small molecular compounds found in traditional Chinese herbal medicine with these inflammatory factors. This integrative approach offers a novel perspective on understanding the underlying mechanisms of placental syndromes and identifying potential drug targets.

Molecular docking refers to the process by which two or more molecules recognize each other through geometric and energetic matching to find the optimal binding mode. Molecular docking is employed to uncover the interaction modes between ligand and target proteins. During the process of molecular docking, we evaluate the docking conformations using the concepts of binding energy and RMSD values. A binding energy below 0 suggests the possibility of free binding between the ligand and protein. A binding energy lower than -5 kcal/mol indicates a strong and favorable interaction between the ligand and protein. RMSD, which stands for root-mean-square deviation, quantifies the similarity between molecular structures and is utilized to assess the accuracy of docking. A smaller RMSD value corresponds to a higher level of docking precision (51).

Traditional Chinese medicine has been used for centuries to treat inflammation related diseases and exhibits anti-inflammatory effects at different levels. In this study, we focused on investigating the role of inflammatory factors in the development of placental syndromes. Based on this, we selected small molecular components of traditional Chinese medicine with anti-inflammatory effects to study, which include flavonoids, phenylpropanoids, terpenoids, etc. Among these small molecular components, there is limited research on puerarin, magnolol, paeoniflorin, tumulosic acid, wogonin, and Atractylenolide I in relation to placental syndromes. Therefore, we selected these six small molecular components for our study (52). We utilized these small molecular compounds as key active ingredients for molecular docking with inflammatory cytokines associated with placental syndromes. All of the binding energy was lower than -5 kcal/mol, and all of the RMSD values in the molecular docking results were less than 2, indicating a strong interaction between these small molecular compounds and the inflammatory cytokines, which making these small molecular compounds to be suitable targets for the treatment of placental syndromes.

In general, we used the two-sample MR method to analyze the causal relationship between inflammatory cytokines and placental syndromes. Additionally, we employed molecular docking method to identify potential therapeutic targets. However, our study has several limitations. Firstly, most current GWAS include a significant percentage of female participants (48-100%), but very few provide sex-specific estimates of the effects. Secondly, GROA is an inflammatory factor known to promote inflammation. Research on the relationship between GROA and pregnancy hypertension remains extremely limited. In our study, we discovered a protective effect of GROA on pregnancy hypertension, although its multifunctionality remains uncertain, as well as the effect of MCSF and PDGFbb on CHP. Further research is needed to establish the correlations between GROA and pregnancy hypertension, as well as the relationships between MCSF and PDGFbb with CHP.

## Data availability statement

The original contributions presented in the study are included in the article/**Supplementary Material**. Further inquiries can be directed to the corresponding author.

## Author contributions

YL: Conceptualization, Data curation, Formal analysis, Funding acquisition, Investigation, Methodology, Resources, Software, Supervision, Visualization, Writing – original draft. SH: Conceptualization, Data curation, Formal analysis, Funding acquisition, Investigation, Methodology, Resources, Software, Supervision, Visualization, Writing – original draft. SC: Conceptualization, Data curation, Formal analysis, Funding acquisition, Investigation, Methodology, Resources, Software, Supervision, Visualization, Writing – original draft. LL: Conceptualization, Data curation, Formal analysis, Funding acquisition, Investigation, Methodology, Visualization, Writing – review & editing. WZ: Conceptualization, Data curation, Formal analysis, Funding acquisition, Resources, Visualization, Writing – review & editing. HX: Conceptualization, Data curation, Formal analysis, Funding acquisition, Investigation, Resources, Writing – review & editing. DY: Conceptualization, Data curation, Methodology, Visualization, Writing – original draft, Writing – review & editing. XZ: Investigation, Methodology, Writing – review & editing. PT: Visualization, Writing – review & editing.
