# Supplementary material for: Investigating the molecular mechanism of traditional Chinese medicine for the treatment of placental syndromes by influencing inflammatory cytokines using the Mendelian randomization and molecular docking technology
Source: Front Endocrinol (Lausanne). 2024 Feb 1;14:1290766. doi: 10.3389/fendo.2023.1290766 (PMC10868387; doi:10.3389/fendo.2023.1290766)
Supplement: Supplementary file 1 [file DataSheet_1.docx]

Supplementary Material

# **Supplementary** Table 1. MR analysis for inflammatory cytokines on placental syndromes

| outcome | Exposure | Number of SNPs | Inverse variance weighted | | | MR-Egger | | | Weighted Median | | |
| --- | --- | --- | --- | --- | --- | --- | --- | --- | --- | --- | --- |
|  |  |  | b | P-value | OR（95%CI） | b | P-value | OR（95%CI） | b | P-value | OR（95%CI） |
| Pregnancy hypetension | bNGF | 4 | -0.0293 | 0.6829 | 0.9712 (0.8440,1.1175) | -0.2831 | 0.5229 | 0.7535 (0.3658,1.5520) | -0.0416 | 0.6344 | 0.9593 (0.8082,1.1386) |
| Pregnancy hypetension | CTACK | 12 | 0.0617 | 0.2227 | 1.0636 (0.9632,1.1744) | -0.0038 | 0.9709 | 0.9962 (0.8152,1.2174) | 0.0437 | 0.4840 | 1.0447 (0.9243,1.1808) |
| Pregnancy hypetension | EOTAXIN | 17 | -0.0681 | 0.1585 | 0.9342 (0.8499,1.0269) | 0.0460 | 0.7027 | 1.0470 (0.8306,1.3198) | -0.0874 | 0.1980 | 0.9164 (0.8022,1.0467) |
| Pregnancy hypetension | FGF-Basic | 7 | -0.0217 | 0.8371 | 0.9785 (0.7958,1.2033) | 0.0714 | 0.8415 | 1.0740 (0.5527,2.0868) | -0.1280 | 0.2965 | 0.8799 (0.6919,1.1188) |
| Pregnancy hypetension | GCSF | 9 | -0.0833 | 0.2346 | 0.9201 (0.8020,1.0555) | -0.0805 | 0.5415 | 0.9227 (0.7216,1.1797) | -0.0604 | 0.5196 | 0.9414 (0.7832,1.1314) |
| Pregnancy hypetension | GROA | 13 | -0.0805 | 0.0437 | 0.9226 (0.8532,0.9977) | -0.0164 | 0.8206 | 0.9837 (0.8565,1.1298) | -0.0445 | 0.3987 | 0.9565 (0.8627,1.0605) |
| Pregnancy hypetension | HGF | 9 | 0.0462 | 0.5589 | 1.0473 (0.8970,1.2228) | 0.1729 | 0.3679 | 1.1887 (0.8359,1.6905) | 0.1279 | 0.2158 | 1.1364 (0.9281,1.3915) |
| Pregnancy hypetension | IFNg | 12 | 0.0229 | 0.7389 | 1.0232 (0.8943,1.1706) | -0.2055 | 0.1245 | 0.8142 (0.6404,1.0353) | 0.0265 | 0.7496 | 1.0268 (0.8728,1.2080) |
| Pregnancy hypetension | IL-10 | 15 | -0.0601 | 0.2550 | 0.9417 (0.8490,1.0444) | -0.3334 | 0.0563 | 0.7165 (0.5246,0.9787) | -0.1666 | 0.0196 | 0.8465 (0.7360,0.9736) |
| Pregnancy hypetension | IL-12p70 | 15 | 0.0010 | 0.9861 | 1.0010 (0.8970,1.1170) | 0.2402 | 0.2550 | 1.2715 (0.8563,1.8881) | 0.0209 | 0.7781 | 1.0211 (0.8832,1.1805) |
| Pregnancy hypetension | IL-13 | 14 | -0.0644 | 0.0814 | 0.9377 (0.8722,1.0081) | 0.0045 | 0.9491 | 1.0045 (0.8770,1.1506) | -0.0627 | 0.1991 | 0.9393 (0.8536,1.0335) |
| Pregnancy hypetension | IL-16 | 10 | -0.0578 | 0.1548 | 0.9439 (0.8717,1.0221) | -0.1178 | 0.1140 | 0.8889 (0.7803,1.0125) | -0.0540 | 0.3129 | 0.9475 (0.8532,1.0521) |
| Pregnancy hypetension | IL-17 | 8 | -0.1216 | 0.0669 | 0.8855 (0.7775,1.0085) | -0.0650 | 0.6173 | 0.9370 (0.7356,1.1936) | -0.0422 | 0.6370 | 0.9587 (0.8045,1.1424) |
| Pregnancy hypetension | IL-18 | 13 | 0.0215 | 0.6068 | 1.0217 (0.9414,1.1088) | 0.0547 | 0.5254 | 1.0562 (0.8969,1.2439) | -0.0035 | 0.9490 | 0.9965 (0.8939,1.1108) |
| Pregnancy hypetension | IL-1ra | 10 | -0.0629 | 0.2005 | 0.9390 (0.8527,1.0340) | -0.2616 | 0.0936 | 0.7698 (0.5879,1.0079) | -0.0367 | 0.5886 | 0.9639 (0.8438,1.1012) |
| Pregnancy hypetension | IL-1β | 3 | -0.0771 | 0.4208 | 0.9258 (0.7674,1.1170) | -0.2808 | 0.3814 | 0.7552 (0.5187,1.0997) | -0.0929 | 0.4280 | 0.9112 (0.7241,1.1467) |
| Pregnancy hypetension | IL-2 | 9 | 0.0279 | 0.6317 | 1.0283 (0.9174,1.1526) | 0.0470 | 0.7209 | 1.0481 (0.8197,1.3401) | 0.0614 | 0.3666 | 1.0633 (0.9307,1.2149) |
| Pregnancy hypetension | IL-2ra | 14 | 0.0614 | 0.1736 | 1.0633 (0.9733,1.1616) | -0.0803 | 0.3616 | 0.9229 (0.7855,1.0843) | 0.0163 | 0.7796 | 1.0165 (0.9067,1.1395) |
| Pregnancy hypetension | IL-4 | 8 | -0.0274 | 0.6944 | 0.9730 (0.8487,1.1155) | -0.0851 | 0.5836 | 0.9184 (0.6831,1.2348) | -0.0366 | 0.6865 | 0.9641 (0.8071,1.1515) |
| Pregnancy hypetension | IL-5 | 8 | -0.0690 | 0.1722 | 0.9333 (0.8453,1.0305) | 0.0518 | 0.6543 | 1.0531 (0.8491,1.3062) | -0.1421 | 0.0352 | 0.8676 (0.7601,0.9902) |
| Pregnancy hypetension | IL-6 | 11 | -0.0447 | 0.5344 | 0.9563 (0.8306,1.1011) | -0.0898 | 0.6134 | 0.9141 (0.6531,1.2795) | -0.0150 | 0.8760 | 0.9852 (0.8164,1.1888) |
| Pregnancy hypetension | IL-7 | 12 | 0.0118 | 0.7869 | 1.0119 (0.9287,1.1026) | -0.0114 | 0.9170 | 0.9887 (0.8021,1.2187) | 0.0353 | 0.5124 | 1.0359 (0.9322,1.1512) |
| Pregnancy hypetension | IL-8 | 8 | -0.0026 | 0.9670 | 0.9974 (0.8819,1.1280) | -0.1474 | 0.2142 | 0.8629 (0.7009,1.0625) | -0.0133 | 0.8496 | 0.9868 (0.8604,1.1318) |
| Pregnancy hypetension | IL-9 | 6 | 0.1990 | 0.0475 | 1.2202 (1.0022,1.4857) | 0.2615 | 0.3866 | 1.2989 (0.7661,2.2022) | 0.1673 | 0.1235 | 1.1821 (0.9555,1.4625) |
| Pregnancy hypetension | IP-10 | 12 | -0.0389 | 0.3766 | 0.9618 (0.8822,1.0485) | -0.0558 | 0.5891 | 0.9458 (0.7775,1.1504) | -0.0620 | 0.3002 | 0.9399 (0.8359,1.0569) |
| Pregnancy hypetension | MCP1 | 16 | -0.0485 | 0.3721 | 0.9526 (0.8563,1.0598) | -0.0086 | 0.9553 | 0.9915 (0.7383,1.3314) | -0.0573 | 0.4341 | 0.9443 (0.8179,1.0902) |
| Pregnancy hypetension | MCP3 | 6 | 0.0337 | 0.6077 | 1.0343 (0.9093,1.1765) | 0.1238 | 0.5443 | 1.1318 (0.7843,1.6333) | 0.1015 | 0.0596 | 1.1068 (0.9959,1.2301) |
| Pregnancy hypetension | MCSF | 12 | -0.0159 | 0.6424 | 0.9843 (0.9205,1.0524) | -0.0707 | 0.3446 | 0.9317 (0.8103,1.0714) | -0.0623 | 0.2043 | 0.9396 (0.8534,1.0345) |
| Pregnancy hypetension | MIF | 10 | 0.1149 | 0.1005 | 1.1217 (0.9780,1.2865) | 0.3079 | 0.0362 | 1.3605 (1.0700,1.7299) | 0.0674 | 0.3638 | 1.0697 (0.9249,1.2373) |
| Pregnancy hypetension | MIG | 13 | 0.0276 | 0.4937 | 1.0280 (0.9498,1.1127) | 0.0158 | 0.8560 | 1.0160 (0.8596,1.2008) | 0.0390 | 0.4903 | 1.0398 (0.9306,1.1618) |
| Pregnancy hypetension | MIP1A | 4 | 0.0279 | 0.8484 | 1.0283 (0.7724,1.3690) | -0.2852 | 0.6307 | 0.7518 (0.2780,2.0331) | -0.0815 | 0.4228 | 0.9217 (0.7551,1.1250) |
| Pregnancy hypetension | MIP1B | 22 | 0.0029 | 0.9462 | 1.0029 (0.9210,1.0922) | -0.0533 | 0.6690 | 0.9481 (0.7453,1.2062) | 0.0289 | 0.6515 | 1.0293 (0.9081,1.1666) |
| Pregnancy hypetension | PDGFbb | 14 | -0.0262 | 0.6618 | 0.9741 (0.8663,1.0954) | 0.1003 | 0.4927 | 1.1055 (0.8373,1.4597) | 0.0584 | 0.4635 | 1.0601 (0.9069,1.2392) |
| Pregnancy hypetension | RANTES | 10 | -0.0699 | 0.1434 | 0.9325 (0.8492,1.0240) | -0.1157 | 0.3776 | 0.8907 (0.6987,1.1355) | -0.0780 | 0.2105 | 0.9250 (0.8187,1.0451) |
| Pregnancy hypetension | SCF | 10 | 0.0415 | 0.6578 | 1.0423 (0.8676,1.2523) | 0.3114 | 0.1182 | 1.3653 (0.9634,1.9350) | 0.1643 | 0.1226 | 1.1785 (0.9567,1.4518) |
| Pregnancy hypetension | SCGFb | 21 | -0.0167 | 0.5904 | 0.9834 (0.9253,1.0452) | -0.1162 | 0.0877 | 0.8903 (0.7845,1.0104) | -0.0284 | 0.5234 | 0.9720 (0.8910,1.0605) |
| Pregnancy hypetension | SDF1A | 9 | -0.1130 | 0.1173 | 0.8931 (0.7754,1.0288) | -0.0431 | 0.7755 | 0.9578 (0.7202,1.2739) | -0.1361 | 0.1235 | 0.8728 (0.7340,1.0378) |
| Pregnancy hypetension | TNFα | 4 | 0.0186 | 0.8000 | 1.0188 (0.8820,1.1768) | -0.0312 | 0.8117 | 0.9693 (0.7734,1.2147) | 0.0142 | 0.8690 | 1.0143 (0.8570,1.2004) |
| Pregnancy hypetension | TNFβ | 5 | -0.0066 | 0.8850 | 0.9935 (0.9089,1.0858) | -0.0765 | 0.4829 | 0.9263 (0.7677,1.1177) | 0.0090 | 0.8710 | 1.0091 (0.9051,1.1250) |
| Pregnancy hypetension | TRAIL | 16 | -0.0856 | 0.1401 | 0.9180 (0.8194,1.0285) | -0.0663 | 0.4617 | 0.9358 (0.7881,1.1112) | -0.0784 | 0.1626 | 0.9246 (0.8283,1.0322) |
| Pregnancy hypetension | VEGF | 18 | 0.0321 | 0.5499 | 1.0327 (0.9294,1.1474) | -0.1017 | 0.3874 | 0.9033 (0.7218,1.1304) | -0.0129 | 0.8527 | 0.9872 (0.8614,1.1313) |
| Preeclampsia | bNGF | 3 | -0.0005 | 0.4562 | 0.9995 (0.9983,1.0007) | -0.0001 | 0.9855 | 0.9999 (0.9913,1.0086) | -0.0003 | 0.6258 | 0.9997 (0.9983,1.0010) |
| Preeclampsia | CTACK | 2 | NA | NA | NA | NA | NA | NA | NA | NA | NA |
| Preeclampsia | EOTAXIN | 7 | 0.0013 | 0.1759 | 1.0013 (0.9994,1.0031) | -0.0101 | 0.3026 | 0.9900 (0.9731,1.0071) | 0.0020 | 0.0273 | 1.0021 (1.0002,1.0039) |
| Preeclampsia | FGF-Basic | 2 | NA | NA | NA | NA | NA | NA | NA | NA | NA |
| Preeclampsia | GCSF | 4 | 0.0010 | 0.2852 | 1.0010 (0.9992,1.0029) | 0.0052 | 0.8160 | 1.0052 (0.9671,1.0450) | 0.0006 | 0.5945 | 1.0006 (0.9983,1.0029) |
| Preeclampsia | GROA | 2 | NA | NA | NA | NA | NA | NA | NA | NA | NA |
| Preeclampsia | HGF | 3 | -0.0028 | 0.0461 | 0.9972 (0.9945,1.0000) | -0.0100 | 0.5113 | 0.9900 (0.9701,1.0104) | -0.0035 | 0.0130 | 0.9965 (0.9937,0.9993) |
| Preeclampsia | IFNg | 4 | -0.0002 | 0.8220 | 0.9998 (0.9982,1.0015) | -0.0006 | 0.9674 | 0.9994 (0.9733,1.0262) | -0.0001 | 0.9296 | 0.9999 (0.9980,1.0018) |
| Preeclampsia | IL-10 | 13 | -0.0008 | 0.0312 | 0.9992 (0.9985,0.9999) | -0.0008 | 0.5272 | 0.9992 (0.9968,1.0016) | -0.0003 | 0.5781 | 0.9997 (0.9987,1.0007) |
| Preeclampsia | IL-12p70 | 8 | -0.0005 | 0.5663 | 0.9995 (0.9979,1.0011) | -0.0025 | 0.4607 | 0.9975 (0.9912,1.0038) | -0.0010 | 0.1829 | 0.9990 (0.9975,1.0005) |
| Preeclampsia | IL-13 | 5 | -0.0001 | 0.9137 | 0.9999 (0.9987,1.0011) | 0.0012 | 0.8558 | 1.0012 (0.9893,1.0133) | -0.0002 | 0.8312 | 0.9998 (0.9984,1.0012) |
| Preeclampsia | IL-16 | 2 | NA | NA | NA | NA | NA | NA | NA | NA | NA |
| Preeclampsia | IL-17 | 2 | NA | NA | NA | NA | NA | NA | NA | NA | NA |
| Preeclampsia | IL-18 | 6 | -0.0008 | 0.0898 | 0.9992 (0.9983,1.0001) | 0.0021 | 0.7819 | 1.0021 (0.9884,1.0159) | -0.0006 | 0.2764 | 0.9994 (0.9982,1.0005) |
| Preeclampsia | IL-1ra | 5 | 0.0000 | 0.9710 | 1.0000 (0.9990,1.0010) | 0.0048 | 0.5238 | 1.0049 (0.9917,1.0182) | 0.0003 | 0.7032 | 1.0003 (0.9989,1.0016) |
| Preeclampsia | IL-1β | 1 | NA | NA | NA | NA | NA | NA | NA | NA | NA |
| Preeclampsia | IL-2 | 4 | 0.0004 | 0.5310 | 1.0004 (0.9991,1.0018) | 0.0076 | 0.0842 | 1.0076 (1.0017,1.0135) | -0.0003 | 0.6320 | 0.9997 (0.9983,1.0011) |
| Preeclampsia | IL-2ra | 5 | 0.0000 | 0.9742 | 1.0000 (0.9987,1.0012) | -0.0088 | 0.9250 | 0.9912 (0.8424,1.1663) | -0.0003 | 0.7097 | 0.9997 (0.9983,1.0012) |
| Preeclampsia | IL-4 | 4 | 0.0000 | 0.9839 | 1.0000 (0.9982,1.0018) | 0.0018 | 0.9507 | 1.0018 (0.9523,1.0539) | 0.0000 | 0.9701 | 1.0000 (0.9980,1.0020) |
| Preeclampsia | IL-5 | 2 | NA | NA | NA | NA | NA | NA | NA | NA | NA |
| Preeclampsia | IL-6 | 5 | -0.0011 | 0.2334 | 0.9989 (0.9972,1.0007) | -0.0091 | 0.1010 | 0.9910 (0.9835,0.9985) | -0.0005 | 0.6092 | 0.9995 (0.9974,1.0015) |
| Preeclampsia | IL-7 | 5 | 0.0002 | 0.7056 | 1.0002 (0.9994,1.0009) | -0.0008 | 0.5586 | 0.9992 (0.9966,1.0017) | 0.0002 | 0.7287 | 1.0002 (0.9992,1.0011) |
| Preeclampsia | IL-8 | 3 | 0.0004 | 0.5840 | 1.0004 (0.9989,1.0019) | 0.0478 | 0.6650 | 1.0489 (0.8928,1.2324) | 0.0001 | 0.9166 | 1.0001 (0.9983,1.0019) |
| Preeclampsia | IL-9 | 0 | NA | NA | NA | NA | NA | NA | NA | NA | NA |
| Preeclampsia | IP-10 | 4 | -0.0001 | 0.9055 | 0.9999 (0.9988,1.0011) | -0.0017 | 0.8498 | 0.9983 (0.9833,1.0136) | 0.0000 | 0.9857 | 1.0000 (0.9987,1.0013) |
| Preeclampsia | MCP1 | 4 | -0.0003 | 0.8637 | 0.9997 (0.9969,1.0026) | 0.0105 | 0.6575 | 1.0106 (0.9709,1.0519) | -0.0012 | 0.2605 | 0.9988 (0.9966,1.0009) |
| Preeclampsia | MCP3 | 3 | -0.0007 | 0.4421 | 0.9993 (0.9975,1.0011) | -0.0125 | 0.3883 | 0.9875 (0.9707,1.0046) | 0.0002 | 0.7658 | 1.0002 (0.9989,1.0014) |
| Preeclampsia | MCSF | 1 | NA | NA | NA | NA | NA | NA | NA | NA | NA |
| Preeclampsia | MIF | 4 | 0.0011 | 0.0521 | 1.0011 (1.0000,1.0021) | -0.0031 | 0.5250 | 0.9969 (0.9890,1.0049) | 0.0009 | 0.1897 | 1.0009 (0.9996,1.0023) |
| Preeclampsia | MIG | 2 | NA | NA | NA | NA | NA | NA | NA | NA | NA |
| Preeclampsia | MIP1A | 2 | NA | NA | NA | NA | NA | NA | NA | NA | NA |
| Preeclampsia | MIP1B | 10 | 0.0001 | 0.8621 | 1.0001 (0.9991,1.0010) | 0.0005 | 0.8153 | 1.0005 (0.9961,1.0050) | -0.0002 | 0.7863 | 0.9998 (0.9985,1.0011) |
| Preeclampsia | PDGFbb | 6 | 0.0003 | 0.6703 | 1.0003 (0.9989,1.0017) | -0.0032 | 0.7187 | 0.9968 (0.9805,1.0133) | 0.0001 | 0.9166 | 1.0001 (0.9983,1.0019) |
| Preeclampsia | RANTES | 3 | -0.0009 | 0.3786 | 0.9991 (0.9970,1.0012) | -0.0055 | 0.8193 | 0.9945 (0.9587,1.0318) | -0.0009 | 0.4000 | 0.9991 (0.9972,1.0011) |
| Preeclampsia | SCF | 3 | -0.0015 | 0.2755 | 0.9985 (0.9958,1.0012) | -0.0143 | 0.2890 | 0.9858 (0.9725,0.9994) | -0.0004 | 0.7459 | 0.9996 (0.9969,1.0022) |
| Preeclampsia | SCGFb | 6 | -0.0006 | 0.1947 | 0.9994 (0.9986,1.0003) | 0.0040 | 0.2540 | 1.0040 (0.9981,1.0098) | -0.0003 | 0.6167 | 0.9997 (0.9986,1.0008) |
| Preeclampsia | SDF1A | 4 | -0.0002 | 0.8185 | 0.9998 (0.9982,1.0014) | 0.0023 | 0.9271 | 1.0023 (0.9588,1.0479) | -0.0003 | 0.7693 | 0.9997 (0.9978,1.0016) |
| Preeclampsia | TNFα | 1 | NA | NA | NA | NA | NA | NA | NA | NA | NA |
| Preeclampsia | TNFβ | 2 | NA | NA | NA | NA | NA | NA | NA | NA | NA |
| Preeclampsia | TRAIL | 6 | 0.0001 | 0.8367 | 1.0001 (0.9989,1.0014) | -0.0005 | 0.9368 | 0.9995 (0.9884,1.0107) | 0.0000 | 0.9881 | 1.0000 (0.9985,1.0015) |
| Preeclampsia | VEGF | 6 | -0.0008 | 0.1755 | 0.9992 (0.9980,1.0004) | 0.0013 | 0.5921 | 1.0013 (0.9970,1.0056) | -0.0004 | 0.5787 | 0.9996 (0.9981,1.0011) |
| CHP | bNGF | 4 | -0.0250 | 0.9063 | 0.9753 (0.6432,1.4789) | 0.2268 | 0.8798 | 1.2545 (0.0936,16.8223) | -0.0317 | 0.8858 | 0.9688 (0.6285,1.4933) |
| CHP | CTACK | 12 | 0.0020 | 0.9852 | 1.0020 (0.8072,1.2439) | -0.1290 | 0.5694 | 0.8790 (0.5719,1.3509) | -0.0763 | 0.6057 | 0.9266 (0.6936,1.2378) |
| CHP | EOTAXIN | 17 | -0.0499 | 0.7250 | 0.9513 (0.7205,1.2561) | -0.0869 | 0.8115 | 0.9167 (0.4544,1.8496) | -0.0158 | 0.9274 | 0.9843 (0.7011,1.3820) |
| CHP | FGF-Basic | 7 | 0.1212 | 0.5576 | 1.1288 (0.7529,1.6923) | 0.1145 | 0.8594 | 1.1213 (0.3365,3.7360) | 0.0413 | 0.8747 | 1.0422 (0.6236,1.7416) |
| CHP | GCSF | 9 | 0.1791 | 0.3024 | 1.1961 (0.8511,1.6810) | 0.2551 | 0.4308 | 1.2905 (0.7097,2.3468) | 0.3215 | 0.1802 | 1.3791 (0.8619,2.2069) |
| CHP | GROA | 13 | -0.0550 | 0.5916 | 0.9465 (0.7742,1.1571) | 0.0760 | 0.6880 | 1.0790 (0.7518,1.5486) | -0.0202 | 0.8771 | 0.9800 (0.7588,1.2657) |
| CHP | HGF | 9 | -0.0871 | 0.6464 | 0.9166 (0.6317,1.3299) | -0.0546 | 0.9070 | 0.9468 (0.3910,2.2926) | -0.0149 | 0.9524 | 0.9852 (0.6045,1.6057) |
| CHP | IFNg | 12 | -0.0743 | 0.6631 | 0.9284 (0.6646,1.2969) | -0.1190 | 0.7496 | 0.8878 (0.4360,1.8078) | -0.2023 | 0.3118 | 0.8169 (0.5520,1.2089) |
| CHP | IL-10 | 15 | -0.2390 | 0.0474 | 0.7874 (0.6217,0.9972) | -0.4827 | 0.2385 | 0.6171 (0.2869,1.3273) | -0.2868 | 0.0945 | 0.7506 (0.5363,1.0506) |
| CHP | IL-12p70 | 15 | 0.0900 | 0.5155 | 1.0942 (0.8341,1.4354) | 0.2490 | 0.6265 | 1.2827 (0.4818,3.4146) | 0.1029 | 0.5804 | 1.1084 (0.7695,1.5966) |
| CHP | IL-13 | 14 | -0.1143 | 0.2817 | 0.8920 (0.7243,1.0984) | 0.0695 | 0.7319 | 1.0719 (0.7270,1.5805) | 0.0441 | 0.7444 | 1.0451 (0.8019,1.3620) |
| CHP | IL-16 | 10 | -0.0970 | 0.3386 | 0.9076 (0.7441,1.1070) | -0.0610 | 0.7239 | 0.9409 (0.6788,1.3040) | -0.1787 | 0.1814 | 0.8363 (0.6435,1.0869) |
| CHP | IL-17 | 8 | -0.1590 | 0.3329 | 0.8530 (0.6183,1.1768) | -0.0546 | 0.8644 | 0.9468 (0.5193,1.7265) | -0.0671 | 0.7611 | 0.9351 (0.6066,1.4415) |
| CHP | IL-18 | 13 | 0.4136 | 0.0000 | 1.5123 (1.2386,1.8465) | 0.4837 | 0.0327 | 1.6220 (1.1003,2.3912) | 0.3852 | 0.0084 | 1.4699 (1.1040,1.9571) |
| CHP | IL-1ra | 10 | -0.0493 | 0.6848 | 0.9519 (0.7503,1.2077) | -0.4596 | 0.2131 | 0.6315 (0.3245,1.2291) | 0.0250 | 0.8690 | 1.0253 (0.7618,1.3799) |
| CHP | IL-1β | 3 | -0.0997 | 0.6742 | 0.9051 (0.5687,1.4406) | -0.2731 | 0.6683 | 0.7610 (0.2994,1.9340) | -0.0817 | 0.7638 | 0.9215 (0.5408,1.5703) |
| CHP | IL-2 | 8 | -0.1132 | 0.4029 | 0.8930 (0.6849,1.1643) | 0.2774 | 0.2954 | 1.3196 (0.8212,2.1206) | 0.0837 | 0.6158 | 1.0873 (0.7841,1.5077) |
| CHP | IL-2ra | 9 | 0.0631 | 0.6082 | 1.0651 (0.8369,1.3555) | -0.0118 | 0.9626 | 0.9882 (0.6133,1.5924) | -0.0843 | 0.5786 | 0.9192 (0.6828,1.2375) |
| CHP | IL-4 | 14 | -0.2882 | 0.1304 | 0.7496 (0.5160,1.0890) | -0.5292 | 0.2236 | 0.5891 (0.2625,1.3220) | -0.4492 | 0.0368 | 0.6381 (0.4185,0.9729) |
| CHP | IL-5 | 8 | -0.2209 | 0.1197 | 0.8018 (0.6070,1.0591) | -0.2738 | 0.4422 | 0.7605 (0.3960,1.4603) | -0.3338 | 0.0501 | 0.7162 (0.5129,1.0002) |
| CHP | IL-6 | 11 | 0.1503 | 0.3854 | 1.1621 (0.8277,1.6316) | 0.0382 | 0.9290 | 1.0389 (0.4590,2.3518) | 0.0772 | 0.7455 | 1.0803 (0.6777,1.7222) |
| CHP | IL-7 | 12 | 0.0356 | 0.6979 | 1.0363 (0.8657,1.2404) | -0.1060 | 0.6319 | 0.8994 (0.5907,1.3695) | 0.0095 | 0.9397 | 1.0095 (0.7898,1.2903) |
| CHP | IL-8 | 8 | 0.1435 | 0.2860 | 1.1543 (0.8868,1.5025) | -0.1015 | 0.6994 | 0.9034 (0.5528,1.4764) | -0.0158 | 0.9313 | 0.9843 (0.6874,1.4096) |
| CHP | IL-9 | 6 | 0.2841 | 0.0908 | 1.3286 (0.9559,1.8467) | 0.5419 | 0.2532 | 1.7193 (0.7754,3.8123) | 0.2257 | 0.3138 | 1.2532 (0.8078,1.9440) |
| CHP | IP-10 | 12 | -0.0366 | 0.7372 | 0.9641 (0.7787,1.1937) | -0.1130 | 0.6576 | 0.8931 (0.5499,1.4507) | 0.0814 | 0.5501 | 1.0849 (0.8305,1.4171) |
| CHP | MCP1 | 16 | -0.0541 | 0.6875 | 0.9474 (0.7280,1.2328) | -0.1559 | 0.6809 | 0.8556 (0.4132,1.7716) | 0.0009 | 0.9962 | 1.0009 (0.6968,1.4376) |
| CHP | MCP3 | 6 | -0.0588 | 0.6322 | 0.9429 (0.7410,1.1998) | -0.0988 | 0.7974 | 0.9059 (0.4471,1.8356) | -0.0378 | 0.7538 | 0.9629 (0.7602,1.2197) |
| CHP | MCSF | 12 | -0.2561 | 0.0024 | 0.7740 (0.6560,0.9133) | -0.2963 | 0.1231 | 0.7436 (0.5267,1.0497) | -0.2353 | 0.0391 | 0.7904 (0.6320,0.9883) |
| CHP | MIF | 10 | -0.0770 | 0.5525 | 0.9259 (0.7181,1.1938) | 0.1030 | 0.7041 | 1.1085 (0.6638,1.8511) | -0.0772 | 0.6548 | 0.9257 (0.6599,1.2986) |
| CHP | MIG | 13 | 0.0420 | 0.6749 | 1.0429 (0.8571,1.2688) | 0.0013 | 0.9950 | 1.0013 (0.6657,1.5062) | 0.1613 | 0.2502 | 1.1750 (0.8926,1.5469) |
| CHP | MIP1A | 4 | -0.1316 | 0.7239 | 0.8767 (0.4224,1.8196) | -0.6516 | 0.6810 | 0.5212 (0.0356,7.6281) | -0.3120 | 0.2034 | 0.7320 (0.4526,1.1838) |
| CHP | MIP1B | 22 | -0.1176 | 0.2747 | 0.8891 (0.7200,1.0979) | -0.6826 | 0.0370 | 0.5053 (0.2777,0.9194) | -0.1582 | 0.3010 | 0.8536 (0.6325,1.1521) |
| CHP | PDGFbb | 14 | -0.3962 | 0.0074 | 0.6729 (0.5034,0.8994) | -0.0403 | 0.9105 | 0.9605 (0.4824,1.9121) | -0.3634 | 0.0601 | 0.6953 (0.4760,1.0156) |
| CHP | RANTES | 10 | -0.0931 | 0.4382 | 0.9111 (0.7201,1.1529) | -0.3076 | 0.3442 | 0.7352 (0.4037,1.3392) | -0.1817 | 0.2472 | 0.8338 (0.6129,1.1344) |
| CHP | SCF | 10 | -0.2208 | 0.2129 | 0.8019 (0.5666,1.1350) | 0.0399 | 0.9176 | 1.0407 (0.5005,2.1636) | -0.1148 | 0.6361 | 0.8915 (0.5541,1.4345) |
| CHP | SCGFb | 21 | 0.1035 | 0.2147 | 1.1090 (0.9418,1.3059) | -0.0195 | 0.9124 | 0.9807 (0.6965,1.3808) | 0.1460 | 0.2023 | 1.1572 (0.9246,1.4483) |
| CHP | SDF1A | 9 | -0.1146 | 0.5240 | 0.8917 (0.6269,1.2685) | -0.1571 | 0.6849 | 0.8546 (0.4128,1.7694) | -0.1862 | 0.4343 | 0.8301 (0.5205,1.3239) |
| CHP | TNFα | 4 | 0.1353 | 0.4582 | 1.1448 (0.8008,1.6366) | 0.0071 | 0.9824 | 1.0071 (0.5763,1.7599) | 0.2712 | 0.2030 | 1.3115 (0.8639,1.9911) |
| CHP | TNFβ | 5 | -0.0379 | 0.7429 | 0.9628 (0.7675,1.2078) | -0.1621 | 0.5742 | 0.8503 (0.5129,1.4098) | -0.0259 | 0.8493 | 0.9744 (0.7459,1.2729) |
| CHP | TRAIL | 16 | 0.0415 | 0.6589 | 1.0424 (0.8669,1.2535) | 0.1013 | 0.4731 | 1.1066 (0.8454,1.4486) | 0.0914 | 0.4866 | 1.0958 (0.8469,1.4177) |
| CHP | VEGF | 18 | 0.0802 | 0.5227 | 1.0835 (0.8473,1.3854) | -0.1995 | 0.4717 | 0.8192 (0.4820,1.3922) | 0.1559 | 0.3494 | 1.1688 (0.8431,1.6202) |
| GDM | bNGF | 4 | 0.0585 | 0.5142 | 1.0602 (0.8894,1.2639) | 0.5481 | 0.3395 | 1.7300 (0.7294,4.1033) | 0.1112 | 0.2749 | 1.1176 (0.9154,1.3645) |
| GDM | CTACK | 12 | -0.0422 | 0.4850 | 0.9587 (0.8515,1.0793) | 0.1306 | 0.2544 | 1.1395 (0.9221,1.4080) | -0.0063 | 0.9288 | 0.9937 (0.8650,1.1416) |
| GDM | EOTAXIN | 17 | 0.0578 | 0.4430 | 1.0595 (0.9140,1.2282) | 0.2302 | 0.2305 | 1.2588 (0.8774,1.8060) | 0.0659 | 0.4155 | 1.0681 (0.9115,1.2517) |
| GDM | FGF-Basic | 7 | -0.0067 | 0.9439 | 0.9933 (0.8234,1.1982) | -0.0044 | 0.9880 | 0.9956 (0.5754,1.7227) | -0.0578 | 0.6361 | 0.9439 (0.7430,1.1990) |
| GDM | GCSF | 9 | -0.0730 | 0.4076 | 0.9296 (0.7820,1.1050) | -0.0052 | 0.9754 | 0.9948 (0.7241,1.3667) | 0.0257 | 0.8146 | 1.0261 (0.8274,1.2725) |
| GDM | GROA | 13 | -0.0548 | 0.3176 | 0.9467 (0.8501,1.0541) | 0.0939 | 0.2933 | 1.0985 (0.9297,1.2979) | -0.0320 | 0.6192 | 0.9685 (0.8536,1.0989) |
| GDM | HGF | 9 | 0.0307 | 0.7170 | 1.0312 (0.8734,1.2175) | -0.0723 | 0.7225 | 0.9303 (0.6342,1.3646) | 0.0700 | 0.5307 | 1.0725 (0.8617,1.3348) |
| GDM | IFNg | 12 | -0.0017 | 0.9809 | 0.9983 (0.8720,1.1430) | -0.1155 | 0.4307 | 0.8910 (0.6764,1.1737) | 0.0063 | 0.9451 | 1.0063 (0.8411,1.2039) |
| GDM | IL-10 | 15 | 0.0146 | 0.7935 | 1.0147 (0.9096,1.1320) | 0.1599 | 0.3937 | 1.1733 (0.8226,1.6736) | 0.0450 | 0.5616 | 1.0461 (0.8985,1.2178) |
| GDM | IL-12p70 | 15 | -0.0649 | 0.3555 | 0.9372 (0.8168,1.0754) | -0.4775 | 0.0622 | 0.6203 (0.3921,0.9814) | -0.1090 | 0.2465 | 0.8967 (0.7457,1.0783) |
| GDM | IL-13 | 14 | -0.0662 | 0.1250 | 0.9360 (0.8601,1.0185) | -0.1058 | 0.2269 | 0.8996 (0.7645,1.0586) | -0.0446 | 0.4548 | 0.9564 (0.8509,1.0750) |
| GDM | IL-16 | 10 | -0.0493 | 0.2890 | 0.9519 (0.8690,1.0427) | 0.0066 | 0.9331 | 1.0066 (0.8667,1.1692) | -0.0272 | 0.6651 | 0.9732 (0.8604,1.1007) |
| GDM | IL-17 | 8 | -0.0604 | 0.4269 | 0.9414 (0.8110,1.0927) | -0.0871 | 0.5609 | 0.9166 (0.6945,1.2096) | -0.0865 | 0.3714 | 0.9172 (0.7588,1.1086) |
| GDM | IL-18 | 13 | 0.0445 | 0.3415 | 1.0455 (0.9539,1.1458) | 0.0791 | 0.3988 | 1.0823 (0.9071,1.2915) | 0.0196 | 0.7555 | 1.0198 (0.9015,1.1535) |
| GDM | IL-1ra | 10 | 0.0186 | 0.7410 | 1.0188 (0.9124,1.1375) | -0.0228 | 0.8878 | 0.9775 (0.7193,1.3283) | 0.0372 | 0.6016 | 1.0379 (0.9026,1.1934) |
| GDM | IL-1β | 3 | 0.1380 | 0.3058 | 1.1480 (0.8815,1.4950) | -0.0980 | 0.7738 | 0.9067 (0.5404,1.5211) | 0.1608 | 0.2791 | 1.1745 (0.8777,1.5715) |
| GDM | IL-2 | 8 | -0.0069 | 0.9007 | 0.9931 (0.8914,1.1065) | -0.0802 | 0.4898 | 0.9230 (0.7454,1.1428) | -0.0358 | 0.6277 | 0.9648 (0.8347,1.1152) |
| GDM | IL-2ra | 9 | 0.0278 | 0.6201 | 1.0282 (0.9212,1.1476) | -0.0797 | 0.4503 | 0.9234 (0.7595,1.1227) | 0.0672 | 0.3563 | 1.0695 (0.9272,1.2338) |
| GDM | IL-4 | 14 | -0.0481 | 0.4851 | 0.9531 (0.8328,1.0908) | -0.1208 | 0.4177 | 0.8862 (0.6683,1.1751) | -0.0446 | 0.6295 | 0.9564 (0.7980,1.1463) |
| GDM | IL-5 | 8 | -0.0992 | 0.1491 | 0.9056 (0.7914,1.0362) | 0.1461 | 0.2909 | 1.1574 (0.9037,1.4822) | -0.0417 | 0.6117 | 0.9592 (0.8166,1.1267) |
| GDM | IL-6 | 11 | 0.0143 | 0.8548 | 1.0144 (0.8706,1.1819) | -0.1080 | 0.5543 | 0.8976 (0.6360,1.2670) | -0.0409 | 0.7050 | 0.9599 (0.7767,1.1864) |
| GDM | IL-7 | 12 | -0.0437 | 0.3043 | 0.9573 (0.8807,1.0405) | -0.1648 | 0.1272 | 0.8480 (0.6983,1.0298) | -0.0308 | 0.5836 | 0.9696 (0.8684,1.0826) |
| GDM | IL-8 | 8 | -0.0620 | 0.3063 | 0.9399 (0.8347,1.0584) | -0.2297 | 0.0876 | 0.7948 (0.6373,0.9912) | -0.0682 | 0.3966 | 0.9341 (0.7979,1.0936) |
| GDM | IL-9 | 6 | -0.0321 | 0.8020 | 0.9684 (0.7533,1.2448) | -0.0147 | 0.9682 | 0.9855 (0.5008,1.9390) | 0.0326 | 0.7804 | 1.0331 (0.8217,1.2989) |
| GDM | IP-10 | 12 | -0.0128 | 0.7996 | 0.9873 (0.8945,1.0897) | 0.1306 | 0.2782 | 1.1395 (0.9115,1.4246) | -0.0229 | 0.7219 | 0.9773 (0.8613,1.1090) |
| GDM | MCP1 | 16 | 0.0871 | 0.1619 | 1.0910 (0.9656,1.2327) | -0.0605 | 0.7306 | 0.9413 (0.6718,1.3190) | 0.1511 | 0.0752 | 1.1631 (0.9848,1.3737) |
| GDM | MCP3 | 6 | -0.0073 | 0.8894 | 0.9927 (0.8951,1.1009) | -0.0759 | 0.6412 | 0.9269 (0.6898,1.2456) | 0.0188 | 0.7428 | 1.0190 (0.9107,1.1401) |
| GDM | MCSF | 12 | -0.0233 | 0.5675 | 0.9770 (0.9019,1.0582) | 0.0041 | 0.9644 | 1.0041 (0.8441,1.1944) | 0.0170 | 0.7613 | 1.0172 (0.9114,1.1352) |
| GDM | MIF | 10 | 0.1415 | 0.0422 | 1.1520 (1.0050,1.3204) | 0.2504 | 0.1097 | 1.2845 (0.9779,1.6874) | 0.2178 | 0.0071 | 1.2433 (1.0611,1.4568) |
| GDM | MIG | 13 | 0.0430 | 0.4566 | 1.0439 (0.9322,1.1689) | -0.0266 | 0.8337 | 0.9738 (0.7641,1.2409) | -0.0267 | 0.6781 | 0.9736 (0.8582,1.1046) |
| GDM | MIP1A | 4 | -0.1053 | 0.3024 | 0.9001 (0.7368,1.0995) | -0.3989 | 0.3414 | 0.6711 (0.3568,1.2620) | -0.0875 | 0.4501 | 0.9162 (0.7301,1.1498) |
| GDM | MIP1B | 22 | -0.0182 | 0.7324 | 0.9820 (0.8850,1.0897) | -0.1719 | 0.2610 | 0.8421 (0.6294,1.1267) | -0.0348 | 0.6286 | 0.9658 (0.8387,1.1121) |
| GDM | PDGFbb | 14 | -0.0240 | 0.7264 | 0.9763 (0.8533,1.1169) | 0.0370 | 0.8234 | 1.0377 (0.7548,1.4268) | -0.0378 | 0.6855 | 0.9629 (0.8018,1.1563) |
| GDM | RANTES | 10 | 0.0381 | 0.4860 | 1.0388 (0.9333,1.1563) | 0.1840 | 0.2139 | 1.2020 (0.9203,1.5700) | 0.0295 | 0.6939 | 1.0299 (0.8893,1.1927) |
| GDM | SCF | 10 | 0.0250 | 0.7587 | 1.0253 (0.8744,1.2022) | -0.0166 | 0.9243 | 0.9836 (0.7063,1.3696) | 0.0398 | 0.6936 | 1.0406 (0.8536,1.2685) |
| GDM | SCGFb | 21 | 0.0105 | 0.7692 | 1.0105 (0.9423,1.0836) | -0.1045 | 0.1740 | 0.9008 (0.7792,1.0413) | -0.0039 | 0.9395 | 0.9961 (0.9017,1.1005) |
| GDM | SDF1A | 9 | -0.0491 | 0.5789 | 0.9521 (0.8007,1.1322) | -0.2639 | 0.1507 | 0.7681 (0.5574,1.0584) | -0.0738 | 0.5105 | 0.9288 (0.7456,1.1572) |
| GDM | TNFα | 4 | 0.0365 | 0.6617 | 1.0372 (0.8808,1.2213) | 0.0149 | 0.9193 | 1.0150 (0.7866,1.3097) | 0.0630 | 0.5193 | 1.0650 (0.8794,1.2898) |
| GDM | TNFβ | 5 | -0.0604 | 0.2042 | 0.9414 (0.8576,1.0334) | -0.1434 | 0.2350 | 0.8664 (0.7167,1.0474) | -0.0787 | 0.1881 | 0.9243 (0.8222,1.0392) |
| GDM | TRAIL | 16 | -0.0084 | 0.8525 | 0.9916 (0.9074,1.0836) | -0.0403 | 0.5611 | 0.9605 (0.8411,1.0968) | -0.0392 | 0.5140 | 0.9616 (0.8548,1.0816) |
| GDM | VEGF | 18 | -0.0491 | 0.3453 | 0.9521 (0.8598,1.0543) | -0.0240 | 0.8386 | 0.9763 (0.7776,1.2256) | -0.0158 | 0.8311 | 0.9843 (0.8512,1.1383) |
| Pregnancy loss | bNGF | 4 | 0.0543 | 0.2840 | 1.0558 (0.9560,1.1659) | 0.0467 | 0.8973 | 1.0478 (0.5602,1.9598) | 0.0103 | 0.8397 | 1.0104 (0.9142,1.1166) |
| Pregnancy loss | CTACK | 12 | -0.0149 | 0.5324 | 0.9852 (0.9400,1.0325) | -0.0003 | 0.9949 | 0.9997 (0.9076,1.1011) | 0.0139 | 0.6676 | 1.0139 (0.9518,1.0801) |
| Pregnancy loss | EOTAXIN | 17 | -0.0337 | 0.2221 | 0.9669 (0.9161,1.0206) | -0.0360 | 0.6127 | 0.9646 (0.8414,1.1058) | -0.0226 | 0.5107 | 0.9776 (0.9140,1.0458) |
| Pregnancy loss | FGF-Basic | 7 | 0.0054 | 0.9206 | 1.0054 (0.9042,1.1180) | -0.0538 | 0.7663 | 0.9477 (0.6775,1.3256) | -0.0181 | 0.7664 | 0.9821 (0.8718,1.1064) |
| Pregnancy loss | GCSF | 9 | -0.0536 | 0.1493 | 0.9478 (0.8811,1.0195) | -0.0842 | 0.2342 | 0.9192 (0.8098,1.0435) | -0.0412 | 0.4034 | 0.9596 (0.8712,1.0570) |
| Pregnancy loss | GROA | 13 | -0.0012 | 0.9540 | 0.9988 (0.9580,1.0413) | 0.0117 | 0.7602 | 1.0118 (0.9401,1.0890) | 0.0137 | 0.6233 | 1.0138 (0.9597,1.0710) |
| Pregnancy loss | HGF | 9 | 0.0388 | 0.3253 | 1.0395 (0.9623,1.1230) | 0.0559 | 0.5416 | 1.0575 (0.8914,1.2545) | 0.0009 | 0.9854 | 1.0009 (0.9050,1.1070) |
| Pregnancy loss | IFNg | 11 | -0.0707 | 0.0517 | 0.9318 (0.8677,1.0005) | 0.0520 | 0.4714 | 1.0533 (0.9199,1.2061) | -0.0755 | 0.1035 | 0.9273 (0.8467,1.0155) |
| Pregnancy loss | IL-10 | 15 | 0.0437 | 0.1099 | 1.0447 (0.9902,1.1023) | 0.1357 | 0.1482 | 1.1453 (0.9634,1.3616) | 0.0486 | 0.1690 | 1.0498 (0.9796,1.1250) |
| Pregnancy loss | IL-12p70 | 15 | -0.0461 | 0.1223 | 0.9550 (0.9007,1.0124) | -0.0675 | 0.5403 | 0.9347 (0.7575,1.1535) | -0.0327 | 0.4276 | 0.9678 (0.8927,1.0492) |
| Pregnancy loss | IL-13 | 14 | 0.0002 | 0.9924 | 1.0002 (0.9568,1.0455) | -0.0333 | 0.4482 | 0.9672 (0.8899,1.0513) | -0.0070 | 0.8034 | 0.9931 (0.9401,1.0490) |
| Pregnancy loss | IL-16 | 10 | -0.0277 | 0.1970 | 0.9726 (0.9325,1.0145) | -0.0249 | 0.5005 | 0.9754 (0.9102,1.0453) | 0.0079 | 0.7890 | 1.0080 (0.9511,1.0683) |
| Pregnancy loss | IL-17 | 8 | 0.0094 | 0.8508 | 1.0094 (0.9152,1.1133) | -0.0584 | 0.5612 | 0.9433 (0.7832,1.1362) | -0.0064 | 0.9068 | 0.9936 (0.8928,1.1058) |
| Pregnancy loss | IL-18 | 13 | -0.0103 | 0.6353 | 0.9898 (0.9486,1.0327) | -0.0147 | 0.7307 | 0.9854 (0.9082,1.0692) | -0.0099 | 0.7399 | 0.9902 (0.9341,1.0496) |
| Pregnancy loss | IL-1ra | 10 | -0.0055 | 0.8733 | 0.9946 (0.9301,1.0635) | 0.1188 | 0.2223 | 1.1262 (0.9445,1.3428) | 0.0182 | 0.6129 | 1.0183 (0.9491,1.0926) |
| Pregnancy loss | IL-1β | 3 | 0.1208 | 0.0174 | 1.1284 (1.0214,1.2465) | 0.0698 | 0.6825 | 1.0723 (0.8341,1.3786) | 0.1488 | 0.0142 | 1.1604 (1.0303,1.3069) |
| Pregnancy loss | IL-2 | 9 | 0.0174 | 0.5559 | 1.0176 (0.9603,1.0783) | 0.0444 | 0.5004 | 1.0454 (0.9259,1.1803) | 0.0099 | 0.7881 | 1.0099 (0.9399,1.0851) |
| Pregnancy loss | IL-2ra | 14 | -0.0448 | 0.0565 | 0.9562 (0.9131,1.0012) | -0.0969 | 0.0606 | 0.9076 (0.8336,0.9882) | -0.0376 | 0.2344 | 0.9631 (0.9051,1.0247) |
| Pregnancy loss | IL-4 | 8 | -0.0009 | 0.9839 | 0.9991 (0.9181,1.0874) | -0.0216 | 0.8206 | 0.9786 (0.8149,1.1751) | -0.0626 | 0.2150 | 0.9394 (0.8509,1.0370) |
| Pregnancy loss | IL-5 | 8 | -0.0258 | 0.4806 | 0.9745 (0.9071,1.0470) | -0.0561 | 0.5339 | 0.9454 (0.8003,1.1169) | -0.0247 | 0.5128 | 0.9756 (0.9059,1.0506) |
| Pregnancy loss | IL-6 | 11 | 0.0208 | 0.5677 | 1.0210 (0.9508,1.0963) | 0.0407 | 0.6309 | 1.0416 (0.8871,1.2231) | -0.0258 | 0.5796 | 0.9746 (0.8897,1.0675) |
| Pregnancy loss | IL-7 | 12 | 0.0107 | 0.6949 | 1.0107 (0.9582,1.0661) | 0.0263 | 0.6994 | 1.0267 (0.9016,1.1691) | 0.0112 | 0.7118 | 1.0113 (0.9528,1.0733) |
| Pregnancy loss | IL-8 | 8 | 0.0596 | 0.0614 | 1.0614 (0.9972,1.1297) | 0.0600 | 0.3843 | 1.0619 (0.9367,1.2038) | 0.0847 | 0.0269 | 1.0884 (1.0097,1.1733) |
| Pregnancy loss | IL-9 | 6 | 0.0223 | 0.6138 | 1.0225 (0.9377,1.1150) | -0.1223 | 0.2429 | 0.8849 (0.7427,1.0542) | 0.0444 | 0.3626 | 1.0454 (0.9501,1.1504) |
| Pregnancy loss | IP-10 | 12 | 0.0000 | 0.9996 | 1.0000 (0.9509,1.0517) | 0.0198 | 0.7495 | 1.0200 (0.9061,1.1482) | -0.0001 | 0.9982 | 0.9999 (0.9404,1.0632) |
| Pregnancy loss | MCP1 | 16 | -0.0239 | 0.4101 | 0.9764 (0.9224,1.0335) | -0.0726 | 0.3800 | 0.9300 (0.7949,1.0880) | -0.0386 | 0.3025 | 0.9621 (0.8940,1.0354) |
| Pregnancy loss | MCP3 | 6 | 0.0298 | 0.2109 | 1.0303 (0.9833,1.0795) | 0.0373 | 0.6246 | 1.0380 (0.9042,1.1915) | 0.0267 | 0.2823 | 1.0271 (0.9783,1.0783) |
| Pregnancy loss | MCSF | 12 | 0.0272 | 0.1465 | 1.0276 (0.9905,1.0660) | 0.0408 | 0.3398 | 1.0416 (0.9618,1.1280) | 0.0294 | 0.2683 | 1.0298 (0.9776,1.0847) |
| Pregnancy loss | MIF | 10 | 0.0091 | 0.7293 | 1.0091 (0.9586,1.0623) | 0.0094 | 0.8602 | 1.0094 (0.9124,1.1168) | 0.0119 | 0.7441 | 1.0120 (0.9420,1.0872) |
| Pregnancy loss | MIG | 13 | -0.0067 | 0.7836 | 0.9934 (0.9472,1.0417) | 0.0180 | 0.7349 | 1.0182 (0.9196,1.1273) | -0.0264 | 0.3852 | 0.9740 (0.9176,1.0337) |
| Pregnancy loss | MIP1A | 4 | -0.0385 | 0.3579 | 0.9622 (0.8864,1.0445) | -0.0046 | 0.9752 | 0.9954 (0.7675,1.2909) | -0.0579 | 0.2531 | 0.9438 (0.8546,1.0423) |
| Pregnancy loss | MIP1B | 22 | -0.0442 | 0.1192 | 0.9567 (0.9049,1.0115) | -0.0993 | 0.2333 | 0.9055 (0.7729,1.0608) | -0.0110 | 0.7514 | 0.9891 (0.9242,1.0586) |
| Pregnancy loss | PDGFbb | 14 | -0.0017 | 0.9615 | 0.9983 (0.9315,1.0698) | -0.0535 | 0.5418 | 0.9479 (0.8020,1.1203) | -0.0054 | 0.9092 | 0.9947 (0.9072,1.0905) |
| Pregnancy loss | RANTES | 10 | 0.0279 | 0.4189 | 1.0283 (0.9610,1.1003) | -0.1166 | 0.1442 | 0.8899 (0.7728,1.0249) | 0.0703 | 0.0485 | 1.0728 (1.0005,1.1505) |
| Pregnancy loss | SCF | 10 | -0.0194 | 0.7041 | 0.9808 (0.8874,1.0841) | 0.1246 | 0.2352 | 1.1327 (0.9364,1.3700) | -0.0724 | 0.1788 | 0.9302 (0.8370,1.0337) |
| Pregnancy loss | SCGFb | 21 | -0.0108 | 0.5799 | 0.9893 (0.9523,1.0278) | -0.0595 | 0.1486 | 0.9423 (0.8721,1.0181) | -0.0230 | 0.3303 | 0.9773 (0.9331,1.0236) |
| Pregnancy loss | SDF1A | 9 | -0.0523 | 0.2524 | 0.9490 (0.8677,1.0380) | -0.0886 | 0.3884 | 0.9152 (0.7578,1.1054) | -0.0297 | 0.5805 | 0.9707 (0.8736,1.0786) |
| Pregnancy loss | TNFα | 4 | -0.0163 | 0.7633 | 0.9838 (0.8848,1.0940) | 0.0219 | 0.8431 | 1.0222 (0.8442,1.2377) | -0.0123 | 0.7938 | 0.9878 (0.9006,1.0834) |
| Pregnancy loss | TNFβ | 5 | -0.0178 | 0.5804 | 0.9823 (0.9221,1.0465) | -0.0141 | 0.8648 | 0.9860 (0.8499,1.1440) | 0.0061 | 0.8364 | 1.0062 (0.9492,1.0665) |
| Pregnancy loss | TRAIL | 16 | -0.0065 | 0.7496 | 0.9935 (0.9545,1.0341) | -0.0172 | 0.5744 | 0.9830 (0.9270,1.0423) | 0.0181 | 0.5330 | 1.0183 (0.9618,1.0781) |
| Pregnancy loss | VEGF | 18 | 0.0272 | 0.2449 | 1.0276 (0.9815,1.0758) | 0.0959 | 0.0763 | 1.1007 (0.9967,1.2155) | 0.0394 | 0.2090 | 1.0401 (0.9782,1.1060) |
| Preterm birth | bNGF | 4 | 0.0405 | 0.6201 | 1.0413 (0.8873,1.2220) | 0.3644 | 0.4777 | 1.4396 (0.6312,3.2833) | -0.0320 | 0.7494 | 0.9685 (0.7957,1.1788) |
| Preterm birth | CTACK | 12 | -0.0563 | 0.3760 | 0.9452 (0.8344,1.0708) | -0.0042 | 0.9748 | 0.9958 (0.7706,1.2868) | -0.0686 | 0.3425 | 0.9337 (0.8104,1.0758) |
| Preterm birth | EOTAXIN | 17 | 0.0330 | 0.6439 | 1.0335 (0.8986,1.1887) | 0.0219 | 0.9051 | 1.0221 (0.7175,1.4562) | 0.0566 | 0.4883 | 1.0583 (0.9016,1.2421) |
| Preterm birth | FGF-Basic | 7 | 0.0460 | 0.6294 | 1.0471 (0.8686,1.2623) | 0.0173 | 0.9533 | 1.0175 (0.5859,1.7670) | 0.0988 | 0.4239 | 1.1038 (0.8665,1.4062) |
| Preterm birth | GCSF | 9 | 0.1192 | 0.1352 | 1.1267 (0.9635,1.3175) | 0.0464 | 0.7491 | 1.0474 (0.7971,1.3764) | 0.1494 | 0.1285 | 1.1611 (0.9577,1.4077) |
| Preterm birth | GROA | 13 | -0.0727 | 0.1704 | 0.9299 (0.8381,1.0317) | -0.0723 | 0.4762 | 0.9303 (0.7677,1.1272) | -0.0759 | 0.2602 | 0.9269 (0.8122,1.0578) |
| Preterm birth | HGF | 9 | 0.1018 | 0.2285 | 1.1071 (0.9381,1.3066) | -0.0722 | 0.7114 | 0.9303 (0.6443,1.3433) | 0.1158 | 0.2749 | 1.1227 (0.9121,1.3821) |
| Preterm birth | IFNg | 12 | -0.0099 | 0.8965 | 0.9902 (0.8530,1.1494) | -0.0315 | 0.8503 | 0.9690 (0.7046,1.3327) | -0.1025 | 0.3035 | 0.9025 (0.7424,1.0972) |
| Preterm birth | IL-10 | 15 | 0.0508 | 0.3604 | 1.0521 (0.9436,1.1730) | 0.2532 | 0.1835 | 1.2881 (0.9048,1.8337) | 0.1001 | 0.1808 | 1.1052 (0.9546,1.2797) |
| Preterm birth | IL-12p70 | 15 | 0.0073 | 0.9090 | 1.0073 (0.8889,1.1415) | 0.0575 | 0.8063 | 1.0592 (0.6752,1.6614) | 0.0016 | 0.9862 | 1.0016 (0.8390,1.1956) |
| Preterm birth | IL-13 | 14 | -0.0233 | 0.6411 | 0.9770 (0.8857,1.0776) | -0.2269 | 0.0140 | 0.7970 (0.6827,0.9304) | 0.0114 | 0.8577 | 1.0115 (0.8926,1.1462) |
| Preterm birth | IL-16 | 10 | -0.0024 | 0.9690 | 0.9976 (0.8829,1.1272) | 0.0994 | 0.3402 | 1.1046 (0.9114,1.3386) | -0.0434 | 0.5355 | 0.9576 (0.8348,1.0984) |
| Preterm birth | IL-17 | 8 | 0.0178 | 0.8144 | 1.0179 (0.8775,1.1809) | -0.0246 | 0.8676 | 0.9757 (0.7399,1.2868) | -0.0406 | 0.6698 | 0.9602 (0.7967,1.1572) |
| Preterm birth | IL-18 | 13 | -0.0023 | 0.9598 | 0.9977 (0.9106,1.0930) | 0.0594 | 0.5214 | 1.0612 (0.8901,1.2653) | 0.0736 | 0.2568 | 1.0763 (0.9478,1.2223) |
| Preterm birth | IL-1ra | 10 | 0.0970 | 0.1153 | 1.1019 (0.9765,1.2433) | 0.2050 | 0.2824 | 1.2275 (0.8662,1.7395) | 0.0303 | 0.6827 | 1.0307 (0.8914,1.1919) |
| Preterm birth | IL-1β | 3 | 0.1162 | 0.4117 | 1.1233 (0.8510,1.4826) | 0.2628 | 0.6015 | 1.3006 (0.6379,2.6519) | 0.1061 | 0.4254 | 1.1119 (0.8566,1.4433) |
| Preterm birth | IL-2 | 9 | 0.0572 | 0.3007 | 1.0589 (0.9501,1.1800) | 0.1446 | 0.2392 | 1.1556 (0.9303,1.4355) | 0.0609 | 0.4337 | 1.0628 (0.9125,1.2379) |
| Preterm birth | IL-2ra | 14 | -0.0208 | 0.7621 | 0.9794 (0.8557,1.1209) | -0.1737 | 0.1847 | 0.8406 (0.6669,1.0594) | -0.0150 | 0.8375 | 0.9851 (0.8534,1.1372) |
| Preterm birth | IL-4 | 8 | 0.0438 | 0.5440 | 1.0448 (0.9069,1.2037) | -0.0054 | 0.9732 | 0.9946 (0.7309,1.3536) | 0.0459 | 0.6460 | 1.0469 (0.8609,1.2732) |
| Preterm birth | IL-5 | 11 | 0.0935 | 0.2506 | 1.0980 (0.9362,1.2877) | 0.1388 | 0.4931 | 1.1489 (0.7913,1.6680) | 0.1014 | 0.2240 | 1.1067 (0.9399,1.3032) |
| Preterm birth | IL-6 | 8 | 0.0172 | 0.8623 | 1.0173 (0.8380,1.2350) | -0.0443 | 0.8548 | 0.9567 (0.6031,1.5174) | 0.0609 | 0.5915 | 1.0628 (0.8509,1.3275) |
| Preterm birth | IL-7 | 12 | 0.0746 | 0.0780 | 1.0775 (0.9917,1.1708) | 0.0552 | 0.5879 | 1.0568 (0.8710,1.2823) | 0.0979 | 0.1027 | 1.1029 (0.9805,1.2405) |
| Preterm birth | IL-8 | 8 | 0.0175 | 0.8010 | 1.0176 (0.8884,1.1656) | -0.0101 | 0.9447 | 0.9900 (0.7534,1.3009) | 0.0547 | 0.5055 | 1.0563 (0.8991,1.2410) |
| Preterm birth | IL-9 | 6 | -0.0551 | 0.4727 | 0.9464 (0.8142,1.1000) | -0.0920 | 0.6460 | 0.9121 (0.6341,1.3121) | -0.0211 | 0.8184 | 0.9791 (0.8174,1.1728) |
| Preterm birth | IP-10 | 12 | 0.0002 | 0.9981 | 1.0002 (0.8755,1.1426) | 0.2018 | 0.1936 | 1.2236 (0.9213,1.6252) | -0.0223 | 0.7661 | 0.9780 (0.8446,1.1324) |
| Preterm birth | MCP1 | 16 | -0.0068 | 0.9212 | 0.9932 (0.8682,1.1363) | 0.0348 | 0.8618 | 1.0354 (0.7049,1.5207) | 0.0432 | 0.6214 | 1.0441 (0.8797,1.2393) |
| Preterm birth | MCP3 | 6 | 0.0073 | 0.8547 | 1.0073 (0.9320,1.0886) | -0.0480 | 0.6697 | 0.9531 (0.7766,1.1698) | -0.0154 | 0.7573 | 0.9847 (0.8930,1.0859) |
| Preterm birth | MCSF | 12 | -0.0478 | 0.2761 | 0.9533 (0.8747,1.0390) | -0.0257 | 0.7942 | 0.9747 (0.8078,1.1760) | -0.0420 | 0.4578 | 0.9588 (0.8581,1.0714) |
| Preterm birth | MIF | 10 | -0.0033 | 0.9526 | 0.9967 (0.8933,1.1121) | -0.0144 | 0.8984 | 0.9857 (0.7951,1.2219) | 0.0360 | 0.6195 | 1.0367 (0.8993,1.1950) |
| Preterm birth | MIG | 13 | -0.0024 | 0.9577 | 0.9976 (0.9115,1.0918) | 0.0186 | 0.8492 | 1.0188 (0.8445,1.2291) | 0.0196 | 0.7628 | 1.0198 (0.8979,1.1582) |
| Preterm birth | MIP1A | 4 | -0.1559 | 0.1984 | 0.8556 (0.6747,1.0851) | 0.3744 | 0.3111 | 1.4542 (0.8423,2.5104) | -0.0815 | 0.4905 | 0.9217 (0.7310,1.1621) |
| Preterm birth | MIP1B | 22 | -0.0120 | 0.8325 | 0.9881 (0.8841,1.1043) | -0.0133 | 0.9360 | 0.9868 (0.7155,1.3609) | -0.0805 | 0.2750 | 0.9227 (0.7986,1.0661) |
| Preterm birth | PDGFbb | 14 | 0.0094 | 0.8906 | 1.0094 (0.8831,1.1538) | 0.1132 | 0.4962 | 1.1199 (0.8163,1.5363) | 0.0383 | 0.6911 | 1.0391 (0.8600,1.2554) |
| Preterm birth | RANTES | 10 | 0.0050 | 0.9421 | 1.0050 (0.8775,1.1512) | -0.0456 | 0.8083 | 0.9555 (0.6692,1.3641) | -0.0113 | 0.8774 | 0.9887 (0.8560,1.1420) |
| Preterm birth | SCF | 10 | -0.0435 | 0.6075 | 0.9574 (0.8110,1.1303) | -0.0094 | 0.9612 | 0.9906 (0.6858,1.4309) | 0.0492 | 0.6409 | 1.0505 (0.8541,1.2920) |
| Preterm birth | SCGFb | 21 | 0.0138 | 0.6968 | 1.0139 (0.9459,1.0868) | 0.0188 | 0.8009 | 1.0190 (0.8822,1.1769) | 0.0288 | 0.5626 | 1.0292 (0.9336,1.1346) |
| Preterm birth | SDF1A | 9 | 0.1614 | 0.1911 | 1.1752 (0.9226,1.4969) | -0.0349 | 0.8932 | 0.9657 (0.5909,1.5783) | 0.0834 | 0.5182 | 1.0870 (0.8439,1.4001) |
| Preterm birth | TNFα | 4 | -0.0774 | 0.3562 | 0.9255 (0.7852,1.0909) | -0.0463 | 0.7580 | 0.9547 (0.7379,1.2352) | -0.1282 | 0.2203 | 0.8797 (0.7166,1.0799) |
| Preterm birth | TNFβ | 5 | 0.0279 | 0.6893 | 1.0283 (0.8969,1.1790) | 0.0247 | 0.8895 | 1.0251 (0.7437,1.4129) | 0.0291 | 0.6756 | 1.0295 (0.8985,1.1796) |
| Preterm birth | TRAIL | 15 | -0.1177 | 0.0254 | 0.8890 (0.8018,0.9856) | -0.0132 | 0.8501 | 0.9869 (0.8633,1.1282) | -0.0339 | 0.5784 | 0.9666 (0.8575,1.0896) |
| Preterm birth | VEGF | 18 | -0.0282 | 0.5734 | 0.9722 (0.8812,1.0726) | -0.0042 | 0.9699 | 0.9958 (0.8045,1.2327) | -0.0771 | 0.2706 | 0.9258 (0.8072,1.0619) |

Abbreviations: SNPs: single nucleotide polymorphisms; b: beta; OR: Odds Ratio; CI: Confidence interval; CHP: chronic hypertension combined with pregnancy; GDM: gestational diabetes mellitus; NA: For the SNPs were less than 3, no further analysis was conducted; bNGF, beta nerve growth factor; CTACK, cutaneous T cell-attracting chemokine; FGF-Basic, basic fibroblast growth factor; GCSF, granulocyte colony-stimulating factor; GROA, growth-regulated oncogene-A; HGF, hepatocyte growth factor; IFNg, interferon gamma; IL, interleukin; IP, interferon gamma-induced protein 10; MCP1, monocyte chemotactic protein 1; MCP3, monocyte-specific chemokine 3; MCSF, macrophage colony-stimulating factor; MIF, macrophage migration inhibitory factor; MIG, monokine induced by interferon gamma; MIP1a, macrophage inflammatory protein-1a; MIP1b, macrophage inflammatory protein-1b; PDGFbb, platelet-derived growth factor BB; RANTES, regulated upon activation normal T cell expressed and secreted factor; SCF, stem cell factor; SCGFβ, stem cell growth factor beta; SDF1α, stromal cellderived factor-1 alpha; TNFα, tumor necrosis factor alpha; TNFβ, tumor necrosis factor beta; TRAIL, TNF-related apoptosis-inducing ligand; VEGF, vascular endothelial growth factor.

1. **Supplementary Table 2. Heterogeneity and pleiotropy tests of inflammatory cytokines on placental syndromes**

| Outcome | Exposure | Q-Pval-MR Egger | Q-Pval-IVW | MR Egger intercept | se | P-value-MR Egger intercept |  |  |  |  |
| --- | --- | --- | --- | --- | --- | --- | --- | --- | --- | --- |
| Pregnancy hypetension | bNGF | 0.3986 | 0.5064 | 0.0362 | 0.0516 | 0.5555 |  |  |  |  |
| Pregnancy hypetension | CTACK | 0.2035 | 0.2270 | 0.0165 | 0.0222 | 0.4755 |  |  |  |  |
| Pregnancy hypetension | EOTAXIN | 0.5187 | 0.5095 | -0.0152 | 0.0144 | 0.3071 |  |  |  |  |
| Pregnancy hypetension | FGF-Basic | 0.0945 | 0.1450 | -0.0123 | 0.0422 | 0.7822 |  |  |  |  |
| Pregnancy hypetension | GCSF | 0.3956 | 0.5017 | -0.0004 | 0.0159 | 0.9787 |  |  |  |  |
| Pregnancy hypetension | GROA | 0.5941 | 0.5712 | -0.0192 | 0.0175 | 0.2947 |  |  |  |  |
| Pregnancy hypetension | HGF | 0.3038 | 0.3356 | -0.0177 | 0.0225 | 0.4554 |  |  |  |  |
| Pregnancy hypetension | IFNg | 0.4618 | 0.2149 | 0.0331 | 0.0155 | 0.0581 |  |  |  |  |
| Pregnancy hypetension | IL-10 | 0.4372 | 0.2877 | 0.0314 | 0.0174 | 0.0943 |  |  |  |  |
| Pregnancy hypetension | IL-12p70 | 0.9238 | 0.8854 | -0.0259 | 0.0210 | 0.2388 |  |  |  |  |
| Pregnancy hypetension | IL-13 | 0.9464 | 0.9168 | -0.0172 | 0.0147 | 0.2626 |  |  |  |  |
| Pregnancy hypetension | IL-16 | 0.7738 | 0.7247 | 0.0188 | 0.0164 | 0.2863 |  |  |  |  |
| Pregnancy hypetension | IL-17 | 0.4337 | 0.5165 | -0.0106 | 0.0195 | 0.6069 |  |  |  |  |
| Pregnancy hypetension | IL-18 | 0.3476 | 0.4097 | -0.0068 | 0.0146 | 0.6506 |  |  |  |  |
| Pregnancy hypetension | IL-1ra | 0.6440 | 0.4922 | 0.0318 | 0.0205 | 0.1604 |  |  |  |  |
| Pregnancy hypetension | IL-1β | 0.7499 | 0.4481 | 0.0421 | 0.0343 | 0.4355 |  |  |  |  |
| Pregnancy hypetension | IL-2 | 0.7672 | 0.4056 | 0.0311 | 0.0152 | 0.0801 |  |  |  |  |
| Pregnancy hypetension | IL-2ra | 0.1423 | 0.1788 | 0.0079 | 0.0183 | 0.6716 |  |  |  |  |
| Pregnancy hypetension | IL-4 | 0.1241 | 0.1849 | -0.0036 | 0.0207 | 0.8664 |  |  |  |  |
| Pregnancy hypetension | IL-5 | 0.5071 | 0.4477 | -0.0259 | 0.0209 | 0.2621 |  |  |  |  |
| Pregnancy hypetension | IL-6 | 0.2815 | 0.3559 | 0.0055 | 0.0187 | 0.7763 |  |  |  |  |
| Pregnancy hypetension | IL-7 | 0.1263 | 0.1713 | 0.0051 | 0.0212 | 0.8144 |  |  |  |  |
| Pregnancy hypetension | IL-8 | 0.3439 | 0.2069 | 0.0306 | 0.0190 | 0.1578 |  |  |  |  |
| Pregnancy hypetension | IL-9 | 0.0277 | 0.0498 | -0.0155 | 0.0610 | 0.8115 |  |  |  |  |
| Pregnancy hypetension | IP-10 | 0.4440 | 0.5308 | 0.0032 | 0.0172 | 0.8550 |  |  |  |  |
| Pregnancy hypetension | MCP1 | 0.8830 | 0.9154 | -0.0053 | 0.0187 | 0.7798 |  |  |  |  |
| Pregnancy hypetension | MCP3 | 0.0021 | 0.0030 | -0.0252 | 0.0483 | 0.6301 |  |  |  |  |
| Pregnancy hypetension | MCSF | 0.4470 | 0.4692 | 0.0188 | 0.0214 | 0.4012 |  |  |  |  |
| Pregnancy hypetension | MIF | 0.1181 | 0.0331 | -0.0414 | 0.0226 | 0.1048 |  |  |  |  |
| Pregnancy hypetension | MIG | 0.4171 | 0.4998 | 0.0029 | 0.0186 | 0.8776 |  |  |  |  |
| Pregnancy hypetension | MIP1A | 0.0141 | 0.0159 | 0.0526 | 0.0807 | 0.5817 |  |  |  |  |
| Pregnancy hypetension | MIP1B | 0.4955 | 0.5437 | 0.0074 | 0.0150 | 0.6298 |  |  |  |  |
| Pregnancy hypetension | PDGFbb | 0.6194 | 0.6167 | -0.0158 | 0.0160 | 0.3443 |  |  |  |  |
| Pregnancy hypetension | RANTES | 0.3695 | 0.4500 | 0.0094 | 0.0232 | 0.6965 |  |  |  |  |
| Pregnancy hypetension | SCF | 0.1868 | 0.0789 | -0.0397 | 0.0230 | 0.1227 |  |  |  |  |
| Pregnancy hypetension | SCGFb | 0.8196 | 0.6882 | 0.0228 | 0.0130 | 0.0948 |  |  |  |  |
| Pregnancy hypetension | SDF1A | 0.9840 | 0.9876 | -0.0094 | 0.0170 | 0.5974 |  |  |  |  |
| Pregnancy hypetension | TNFα | 0.8980 | 0.9118 | 0.0149 | 0.0264 | 0.6302 |  |  |  |  |
| Pregnancy hypetension | TNFβ | 0.2757 | 0.3108 | 0.0216 | 0.0257 | 0.4632 |  |  |  |  |
| Pregnancy hypetension | TRAIL | 0.0020 | 0.0031 | -0.0045 | 0.0148 | 0.7686 |  |  |  |  |
| Pregnancy hypetension | VEGF | 0.1155 | 0.0857 | 0.0224 | 0.0170 | 0.2064 |  |  |  |  |
| Preeclampsia | bNGF | 0.0971 | 0.1274 | 0.0006 | 0.0009 | 0.6089 |  |  |  |  |
| Preeclampsia | CTACK | NA | NA | NA | NA | NA |  |  |  |  |
| Preeclampsia | EOTAXIN | 0.6520 | 0.7688 | 0.0009 | 0.0765 | 0.9912 |  |  |  |  |
| Preeclampsia | FGF-Basic | NA | NA | NA | NA | NA |  |  |  |  |
| Preeclampsia | GCSF | 0.9798 | 0.9974 | -0.0001 | 0.0021 | 0.9501 |  |  |  |  |
| Preeclampsia | GROA | NA | NA | NA | NA | NA |  |  |  |  |
| Preeclampsia | HGF | 0.1745 | 0.2930 | -0.0055 | 0.0096 | 0.6674 |  |  |  |  |
| Preeclampsia | IFNg | 0.7536 | 0.8945 | 0.0002 | 0.0009 | 0.8556 |  |  |  |  |
| Preeclampsia | IL-10 | 0.7353 | 0.8005 | 0.0101 | 0.0453 | 0.8281 |  |  |  |  |
| Preeclampsia | IL-12p70 | 0.4381 | 0.4087 | 0.0514 | 0.0446 | 0.2929 |  |  |  |  |
| Preeclampsia | IL-13 | 0.5978 | 0.6618 | -0.0006 | 0.0008 | 0.5211 |  |  |  |  |
| Preeclampsia | IL-16 | NA | NA | NA | NA | NA |  |  |  |  |
| Preeclampsia | IL-17 | NA | NA | NA | NA | NA |  |  |  |  |
| Preeclampsia | IL-18 | 0.8840 | 0.6172 | -0.0006 | 0.0004 | 0.1980 |  |  |  |  |
| Preeclampsia | IL-1ra | 0.6351 | 0.1057 | -0.0009 | 0.0004 | 0.0929 |  |  |  |  |
| Preeclampsia | IL-1β | NA | NA | NA | NA | NA |  |  |  |  |
| Preeclampsia | IL-2 | 0.0166 | 0.0250 | -0.0009 | 0.0018 | 0.6493 |  |  |  |  |
| Preeclampsia | IL-2ra | 0.7541 | 0.2282 | 0.0007 | 0.0003 | 0.1257 |  |  |  |  |
| Preeclampsia | IL-4 | 0.4715 | 0.4626 | 0.0006 | 0.0005 | 0.4102 |  |  |  |  |
| Preeclampsia | IL-5 | NA | NA | NA | NA | NA |  |  |  |  |
| Preeclampsia | IL-6 | 0.3710 | 0.4293 | 0.0002 | 0.0002 | 0.4747 |  |  |  |  |
| Preeclampsia | IL-7 | 0.1990 | 0.2744 | 0.0385 | 0.0695 | 0.6185 |  |  |  |  |
| Preeclampsia | IL-8 | 0.0445 | 0.0033 | 0.0026 | 0.0019 | 0.4048 |  |  |  |  |
| Preeclampsia | IL-9 | NA | NA | NA | NA | NA |  |  |  |  |
| Preeclampsia | IP-10 | 0.2681 | 0.4490 | -0.0002 | 0.0020 | 0.9213 |  |  |  |  |
| Preeclampsia | MCP1 | 0.1188 | 0.2270 | -0.0359 | 0.1853 | 0.8643 |  |  |  |  |
| Preeclampsia | MCP3 | 0.0235 | 0.0662 | 0.0006 | 0.0025 | 0.8486 |  |  |  |  |
| Preeclampsia | MCSF | NA | NA | NA | NA | NA |  |  |  |  |
| Preeclampsia | MIF | 0.0063 | 0.0120 | 0.0874 | 0.2179 | 0.7272 |  |  |  |  |
| Preeclampsia | MIG | NA | NA | NA | NA | NA |  |  |  |  |
| Preeclampsia | MIP1A | NA | NA | NA | NA | NA |  |  |  |  |
| Preeclampsia | MIP1B | 0.5653 | 0.5992 | -0.0382 | 0.0482 | 0.4507 |  |  |  |  |
| Preeclampsia | PDGFbb | 0.7971 | 0.8918 | 0.0001 | 0.0005 | 0.9187 |  |  |  |  |
| Preeclampsia | RANTES | 0.3198 | 0.1105 | 0.0011 | 0.0006 | 0.3157 |  |  |  |  |
| Preeclampsia | SCF | 0.5882 | 0.7906 | 0.0357 | 0.0849 | 0.7466 |  |  |  |  |
| Preeclampsia | SCGFb | 0.3293 | 0.3337 | -0.0002 | 0.0002 | 0.3814 |  |  |  |  |
| Preeclampsia | SDF1A | 0.4343 | 0.5700 | 0.0383 | 0.0653 | 0.6172 |  |  |  |  |
| Preeclampsia | TNFα | NA | NA | NA | NA | NA |  |  |  |  |
| Preeclampsia | TNFβ | NA | NA | NA | NA | NA |  |  |  |  |
| Preeclampsia | TRAIL | 0.8101 | 0.8382 | -0.0639 | 0.0917 | 0.5243 |  |  |  |  |
| Preeclampsia | VEGF | 0.0367 | 0.0679 | 0.0112 | 0.0931 | 0.9103 |  |  |  |  |
| CHP | bNGF | 0.3349 | 0.3090 | -0.0698 | 0.0616 | 0.3748 |  |  |  |  |
| CHP | CTACK | 0.3180 | 0.1608 | -0.0437 | 0.0235 | 0.0931 |  |  |  |  |
| CHP | EOTAXIN | 0.0223 | 0.0189 | -0.0230 | 0.0225 | 0.3215 |  |  |  |  |
| CHP | FGF-Basic | 0.6458 | 0.7634 | -0.0003 | 0.0349 | 0.9933 |  |  |  |  |
| CHP | GCSF | 0.2317 | 0.2906 | -0.0105 | 0.0205 | 0.6256 |  |  |  |  |
| CHP | GROA | 0.3369 | 0.1343 | -0.0449 | 0.0212 | 0.0574 |  |  |  |  |
| CHP | HGF | 0.3693 | 0.4357 | 0.0145 | 0.0245 | 0.5735 |  |  |  |  |
| CHP | IFNg | 0.9748 | 0.9663 | 0.0165 | 0.0178 | 0.3748 |  |  |  |  |
| CHP | IL-10 | 0.5774 | 0.5976 | -0.0167 | 0.0198 | 0.4147 |  |  |  |  |
| CHP | IL-12p70 | 0.4222 | 0.2674 | 0.0446 | 0.0243 | 0.0894 |  |  |  |  |
| CHP | IL-13 | 0.3528 | 0.4043 | 0.0099 | 0.0176 | 0.5833 |  |  |  |  |
| CHP | IL-16 | 0.4079 | 0.4231 | -0.0176 | 0.0190 | 0.3809 |  |  |  |  |
| CHP | IL-17 | 0.9829 | 0.9927 | 0.0050 | 0.0223 | 0.8306 |  |  |  |  |
| CHP | IL-18 | 0.7761 | 0.8243 | -0.0071 | 0.0158 | 0.6616 |  |  |  |  |
| CHP | IL-1ra | 0.8807 | 0.9235 | 0.0066 | 0.0234 | 0.7840 |  |  |  |  |
| CHP | IL-1β | 0.2230 | 0.2153 | 0.0492 | 0.0476 | 0.4895 |  |  |  |  |
| CHP | IL-2 | 0.9020 | 0.9036 | 0.0141 | 0.0182 | 0.4654 |  |  |  |  |
| CHP | IL-2ra | 0.3332 | 0.2743 | 0.0237 | 0.0185 | 0.2407 |  |  |  |  |
| CHP | IL-4 | 0.9354 | 0.9491 | 0.0100 | 0.0174 | 0.5755 |  |  |  |  |
| CHP | IL-5 | 0.5325 | 0.1959 | -0.0525 | 0.0240 | 0.0713 |  |  |  |  |
| CHP | IL-6 | 0.5621 | 0.5970 | 0.0149 | 0.0192 | 0.4578 |  |  |  |  |
| CHP | IL-7 | 0.9128 | 0.8388 | 0.0267 | 0.0197 | 0.2057 |  |  |  |  |
| CHP | IL-8 | 0.8608 | 0.5771 | 0.0358 | 0.0203 | 0.1280 |  |  |  |  |
| CHP | IL-9 | 0.0075 | 0.0159 | -0.0044 | 0.0785 | 0.9583 |  |  |  |  |
| CHP | IP-10 | 0.7868 | 0.6863 | -0.0276 | 0.0197 | 0.1908 |  |  |  |  |
| CHP | MCP1 | 0.4994 | 0.5109 | 0.0197 | 0.0214 | 0.3732 |  |  |  |  |
| CHP | MCP3 | 0.0805 | 0.1164 | 0.0191 | 0.0390 | 0.6488 |  |  |  |  |
| CHP | MCSF | 0.2964 | 0.3652 | -0.0094 | 0.0266 | 0.7320 |  |  |  |  |
| CHP | MIF | 0.1334 | 0.1335 | -0.0232 | 0.0256 | 0.3910 |  |  |  |  |
| CHP | MIG | 0.0805 | 0.0956 | 0.0173 | 0.0269 | 0.5350 |  |  |  |  |
| CHP | MIP1A | 0.2679 | 0.2777 | 0.0494 | 0.0513 | 0.4376 |  |  |  |  |
| CHP | MIP1B | 0.3114 | 0.2964 | 0.0201 | 0.0182 | 0.2815 |  |  |  |  |
| CHP | PDGFbb | 0.8620 | 0.8967 | -0.0076 | 0.0184 | 0.6855 |  |  |  |  |
| CHP | RANTES | 0.5429 | 0.5034 | -0.0298 | 0.0255 | 0.2761 |  |  |  |  |
| CHP | SCF | 0.9671 | 0.9820 | 0.0062 | 0.0220 | 0.7863 |  |  |  |  |
| CHP | SCGFb | 0.7679 | 0.6261 | 0.0264 | 0.0149 | 0.0922 |  |  |  |  |
| CHP | SDF1A | 0.4281 | 0.3165 | 0.0292 | 0.0192 | 0.1729 |  |  |  |  |
| CHP | TNFα | 0.8501 | 0.9460 | 0.0065 | 0.0302 | 0.8487 |  |  |  |  |
| CHP | TNFβ | 0.5861 | 0.5739 | 0.0256 | 0.0260 | 0.3973 |  |  |  |  |
| CHP | TRAIL | 0.3482 | 0.3883 | 0.0074 | 0.0114 | 0.5299 |  |  |  |  |
| CHP | VEGF | 0.3199 | 0.3803 | -0.0042 | 0.0173 | 0.8107 |  |  |  |  |
| GDM | bNGF | 0.1845 | 0.3263 | -0.0004 | 0.0017 | 0.8506 |  |  |  |  |
| GDM | CTACK | 0.8707 | 0.8882 | 0.0329 | 0.0476 | 0.5049 |  |  |  |  |
| GDM | EOTAXIN | 0.0929 | 0.1242 | 0.0050 | 0.0437 | 0.9112 |  |  |  |  |
| GDM | FGF-Basic | 0.0766 | 0.0382 | 0.0010 | 0.0008 | 0.2501 |  |  |  |  |
| GDM | GCSF | 0.4741 | 0.5727 | -0.0117 | 0.0385 | 0.7708 |  |  |  |  |
| GDM | GROA | 0.8594 | 0.9054 | 0.0000 | 0.0001 | 0.9894 |  |  |  |  |
| GDM | HGF | 0.2928 | 0.3880 | -0.0045 | 0.0562 | 0.9379 |  |  |  |  |
| GDM | IFNg | 0.1508 | 0.2043 | 0.0065 | 0.0460 | 0.8902 |  |  |  |  |
| GDM | IL-10 | 0.5377 | 0.5816 | 0.0280 | 0.0427 | 0.5235 |  |  |  |  |
| GDM | IL-12p70 | 0.4852 | 0.5555 | -0.0172 | 0.0519 | 0.7458 |  |  |  |  |
| GDM | IL-13 | 0.1916 | 0.1737 | -0.0459 | 0.0419 | 0.2947 |  |  |  |  |
| GDM | IL-16 | 0.2216 | 0.2953 | 0.0000 | 0.0002 | 0.8394 |  |  |  |  |
| GDM | IL-17 | 0.0150 | 0.0177 | 0.0002 | 0.0003 | 0.5296 |  |  |  |  |
| GDM | IL-18 | 0.3587 | 0.3781 | -0.0391 | 0.0454 | 0.4085 |  |  |  |  |
| GDM | IL-1ra | 0.8861 | 0.9277 | -0.0111 | 0.0409 | 0.7921 |  |  |  |  |
| GDM | IL-1β | 0.5148 | 0.8062 | 0.0000 | 0.0006 | 0.9483 |  |  |  |  |
| GDM | IL-2 | 0.6696 | 0.7547 | -0.0194 | 0.0482 | 0.7008 |  |  |  |  |
| GDM | IL-2ra | 0.2042 | 0.2709 | 0.0163 | 0.0446 | 0.7264 |  |  |  |  |
| GDM | IL-4 | 0.0616 | 0.0721 | 0.0329 | 0.0496 | 0.5199 |  |  |  |  |
| GDM | IL-5 | 0.4938 | 0.2629 | -0.0742 | 0.0399 | 0.1122 |  |  |  |  |
| GDM | IL-6 | 0.3333 | 0.4131 | 0.0135 | 0.0451 | 0.7720 |  |  |  |  |
| GDM | IL-7 | 0.6798 | 0.7124 | 0.0312 | 0.0427 | 0.4819 |  |  |  |  |
| GDM | IL-8 | 0.1722 | 0.2475 | 0.0114 | 0.0634 | 0.8638 |  |  |  |  |
| GDM | IL-9 | 0.5774 | 0.6918 | -0.0004 | 0.0009 | 0.7023 |  |  |  |  |
| GDM | IP-10 | 0.7092 | 0.7752 | 0.0147 | 0.0427 | 0.7379 |  |  |  |  |
| GDM | MCP1 | 0.9179 | 0.9425 | 0.0136 | 0.0462 | 0.7729 |  |  |  |  |
| GDM | MCP3 | 0.2745 | 0.3738 | 0.0003 | 0.0007 | 0.6933 |  |  |  |  |
| GDM | MCSF | 0.8689 | 0.9110 | 0.0138 | 0.0530 | 0.7999 |  |  |  |  |
| GDM | MIF | 0.9402 | 0.8696 | 0.0656 | 0.0508 | 0.2320 |  |  |  |  |
| GDM | MIG | 0.4487 | 0.5199 | -0.0142 | 0.0345 | 0.6880 |  |  |  |  |
| GDM | MIP1A | 0.9105 | 0.9794 | 0.0000 | 0.0011 | 0.9773 |  |  |  |  |
| GDM | MIP1B | 0.9773 | 0.8962 | 0.0737 | 0.0373 | 0.0620 |  |  |  |  |
| GDM | PDGFbb | 0.5927 | 0.5679 | -0.0444 | 0.0397 | 0.2859 |  |  |  |  |
| GDM | RANTES | 0.3061 | 0.3347 | -0.0385 | 0.0482 | 0.4480 |  |  |  |  |
| GDM | SCF | 0.3704 | 0.4091 | 0.0438 | 0.0572 | 0.4659 |  |  |  |  |
| GDM | SCGFb | 0.2460 | 0.2611 | 0.0282 | 0.0351 | 0.4315 |  |  |  |  |
| GDM | SDF1A | 0.4041 | 0.5088 | 0.0057 | 0.0431 | 0.8990 |  |  |  |  |
| GDM | TNFα | 0.2449 | 0.4187 | 0.0011 | 0.0104 | 0.9252 |  |  |  |  |
| GDM | TNFβ | 0.1333 | 0.2249 | -0.0002 | 0.0008 | 0.8469 |  |  |  |  |
| GDM | TRAIL | 0.6319 | 0.6762 | -0.0140 | 0.0234 | 0.5601 |  |  |  |  |
| GDM | VEGF | 0.1821 | 0.1602 | 0.0468 | 0.0403 | 0.2619 |  |  |  |  |
| Pregnancy loss | bNGF | 0.0711 | 0.1519 | 0.0011 | 0.0447 | 0.9828 |  |  |  |  |
| Pregnancy loss | CTACK | 0.3548 | 0.4295 | -0.0037 | 0.0107 | 0.7381 |  |  |  |  |
| Pregnancy loss | EOTAXIN | 0.2444 | 0.3034 | 0.0003 | 0.0085 | 0.9705 |  |  |  |  |
| Pregnancy loss | FGF-Basic | 0.1268 | 0.1841 | 0.0078 | 0.0214 | 0.7282 |  |  |  |  |
| Pregnancy loss | GCSF | 0.9606 | 0.9696 | 0.0047 | 0.0082 | 0.5816 |  |  |  |  |
| Pregnancy loss | GROA | 0.9669 | 0.9779 | -0.0039 | 0.0093 | 0.6830 |  |  |  |  |
| Pregnancy loss | HGF | 0.7719 | 0.8464 | -0.0024 | 0.0109 | 0.8316 |  |  |  |  |
| Pregnancy loss | IFNg | 0.5649 | 0.2982 | -0.0172 | 0.0085 | 0.0733 |  |  |  |  |
| Pregnancy loss | IL-10 | 0.3584 | 0.3428 | -0.0106 | 0.0096 | 0.2935 |  |  |  |  |
| Pregnancy loss | IL-12p70 | 0.9205 | 0.9468 | 0.0023 | 0.0112 | 0.8388 |  |  |  |  |
| Pregnancy loss | IL-13 | 0.1906 | 0.1913 | 0.0084 | 0.0090 | 0.3689 |  |  |  |  |
| Pregnancy loss | IL-16 | 0.4226 | 0.5219 | -0.0009 | 0.0088 | 0.9217 |  |  |  |  |
| Pregnancy loss | IL-17 | 0.0527 | 0.0524 | 0.0126 | 0.0149 | 0.4297 |  |  |  |  |
| Pregnancy loss | IL-18 | 0.6884 | 0.7620 | 0.0009 | 0.0073 | 0.9033 |  |  |  |  |
| Pregnancy loss | IL-1ra | 0.1484 | 0.0811 | -0.0199 | 0.0134 | 0.1765 |  |  |  |  |
| Pregnancy loss | IL-1β | 0.2060 | 0.3797 | 0.0106 | 0.0230 | 0.7258 |  |  |  |  |
| Pregnancy loss | IL-2 | 0.9225 | 0.7993 | 0.0115 | 0.0081 | 0.1962 |  |  |  |  |
| Pregnancy loss | IL-2ra | 0.0232 | 0.0340 | 0.0029 | 0.0113 | 0.8041 |  |  |  |  |
| Pregnancy loss | IL-4 | 0.1822 | 0.2368 | -0.0052 | 0.0103 | 0.6318 |  |  |  |  |
| Pregnancy loss | IL-5 | 0.0508 | 0.0749 | 0.0065 | 0.0162 | 0.7023 |  |  |  |  |
| Pregnancy loss | IL-6 | 0.8074 | 0.8648 | -0.0024 | 0.0089 | 0.7916 |  |  |  |  |
| Pregnancy loss | IL-7 | 0.0241 | 0.0362 | -0.0035 | 0.0132 | 0.7989 |  |  |  |  |
| Pregnancy loss | IL-8 | 0.1784 | 0.2587 | -0.0001 | 0.0115 | 0.9930 |  |  |  |  |
| Pregnancy loss | IL-9 | 0.3730 | 0.1791 | 0.0361 | 0.0203 | 0.1502 |  |  |  |  |
| Pregnancy loss | IP-10 | 0.2177 | 0.2752 | -0.0038 | 0.0104 | 0.7221 |  |  |  |  |
| Pregnancy loss | MCP1 | 0.8563 | 0.8766 | 0.0065 | 0.0100 | 0.5247 |  |  |  |  |
| Pregnancy loss | MCP3 | 0.0835 | 0.1426 | -0.0021 | 0.0181 | 0.9145 |  |  |  |  |
| Pregnancy loss | MCSF | 0.3234 | 0.3930 | -0.0046 | 0.0122 | 0.7118 |  |  |  |  |
| Pregnancy loss | MIF | 0.5752 | 0.6739 | -0.0001 | 0.0095 | 0.9945 |  |  |  |  |
| Pregnancy loss | MIG | 0.1832 | 0.2210 | -0.0062 | 0.0114 | 0.5982 |  |  |  |  |
| Pregnancy loss | MIP1A | 0.3539 | 0.5413 | -0.0057 | 0.0212 | 0.8128 |  |  |  |  |
| Pregnancy loss | MIP1B | 0.0584 | 0.0645 | 0.0072 | 0.0099 | 0.4744 |  |  |  |  |
| Pregnancy loss | PDGFbb | 0.2228 | 0.2531 | 0.0065 | 0.0096 | 0.5149 |  |  |  |  |
| Pregnancy loss | RANTES | 0.2405 | 0.0557 | 0.0295 | 0.0135 | 0.0598 |  |  |  |  |
| Pregnancy loss | SCF | 0.1489 | 0.0598 | -0.0213 | 0.0126 | 0.1295 |  |  |  |  |
| Pregnancy loss | SCGFb | 0.1633 | 0.1214 | 0.0112 | 0.0079 | 0.1757 |  |  |  |  |
| Pregnancy loss | SDF1A | 0.1314 | 0.1764 | 0.0049 | 0.0113 | 0.6769 |  |  |  |  |
| Pregnancy loss | TNFα | 0.0760 | 0.1205 | -0.0115 | 0.0225 | 0.6612 |  |  |  |  |
| Pregnancy loss | TNFβ | 0.0373 | 0.0756 | -0.0012 | 0.0203 | 0.9578 |  |  |  |  |
| Pregnancy loss | TRAIL | 0.5552 | 0.6118 | 0.0025 | 0.0051 | 0.6327 |  |  |  |  |
| Pregnancy loss | VEGF | 0.6291 | 0.5285 | -0.0115 | 0.0075 | 0.1454 |  |  |  |  |
| Preterm birth | bNGF | 0.3717 | 0.4582 | -0.0462 | 0.0588 | 0.5147 |  |  |  |  |
| Preterm birth | CTACK | 0.0769 | 0.1008 | -0.0131 | 0.0284 | 0.6543 |  |  |  |  |
| Preterm birth | EOTAXIN | 0.0292 | 0.0421 | 0.0015 | 0.0220 | 0.9472 |  |  |  |  |
| Preterm birth | FGF-Basic | 0.5767 | 0.7004 | 0.0038 | 0.0350 | 0.9180 |  |  |  |  |
| Preterm birth | GCSF | 0.9144 | 0.9299 | 0.0113 | 0.0176 | 0.5436 |  |  |  |  |
| Preterm birth | GROA | 0.1274 | 0.1742 | -0.0001 | 0.0243 | 0.9960 |  |  |  |  |
| Preterm birth | HGF | 0.8638 | 0.8287 | 0.0244 | 0.0234 | 0.3328 |  |  |  |  |
| Preterm birth | IFNg | 0.1982 | 0.2614 | 0.0031 | 0.0205 | 0.8819 |  |  |  |  |
| Preterm birth | IL-10 | 0.9715 | 0.9506 | -0.0232 | 0.0197 | 0.2589 |  |  |  |  |
| Preterm birth | IL-12p70 | 0.6755 | 0.7418 | -0.0054 | 0.0239 | 0.8236 |  |  |  |  |
| Preterm birth | IL-13 | 0.6971 | 0.1443 | 0.0509 | 0.0167 | 0.0101 |  |  |  |  |
| Preterm birth | IL-16 | 0.0973 | 0.0600 | -0.0319 | 0.0243 | 0.2256 |  |  |  |  |
| Preterm birth | IL-17 | 0.4549 | 0.5574 | 0.0079 | 0.0222 | 0.7344 |  |  |  |  |
| Preterm birth | IL-18 | 0.6515 | 0.6747 | -0.0127 | 0.0157 | 0.4376 |  |  |  |  |
| Preterm birth | IL-1ra | 0.2427 | 0.2844 | -0.0173 | 0.0266 | 0.5338 |  |  |  |  |
| Preterm birth | IL-1β | 0.0960 | 0.1854 | -0.0303 | 0.0651 | 0.7227 |  |  |  |  |
| Preterm birth | IL-2 | 0.1323 | 0.0609 | 0.0336 | 0.0218 | 0.1681 |  |  |  |  |
| Preterm birth | IL-2ra | 0.2878 | 0.3493 | 0.0068 | 0.0190 | 0.7280 |  |  |  |  |
| Preterm birth | IL-4 | 0.4416 | 0.4642 | -0.0167 | 0.0183 | 0.3971 |  |  |  |  |
| Preterm birth | IL-5 | 0.0677 | 0.0965 | 0.0075 | 0.0256 | 0.7777 |  |  |  |  |
| Preterm birth | IL-6 | 0.0328 | 0.0531 | -0.0097 | 0.0362 | 0.7974 |  |  |  |  |
| Preterm birth | IL-7 | 0.4890 | 0.5751 | 0.0043 | 0.0196 | 0.8319 |  |  |  |  |
| Preterm birth | IL-8 | 0.1692 | 0.2413 | 0.0058 | 0.0250 | 0.8229 |  |  |  |  |
| Preterm birth | IL-9 | 0.9076 | 0.9574 | 0.0092 | 0.0420 | 0.8379 |  |  |  |  |
| Preterm birth | IP-10 | 0.0918 | 0.0426 | -0.0388 | 0.0250 | 0.1517 |  |  |  |  |
| Preterm birth | MCP1 | 0.1924 | 0.2426 | -0.0055 | 0.0244 | 0.8235 |  |  |  |  |
| Preterm birth | MCP3 | 0.7990 | 0.8517 | 0.0154 | 0.0270 | 0.5981 |  |  |  |  |
| Preterm birth | MCSF | 0.1805 | 0.2366 | -0.0076 | 0.0288 | 0.7978 |  |  |  |  |
| Preterm birth | MIF | 0.9056 | 0.9448 | 0.0024 | 0.0203 | 0.9090 |  |  |  |  |
| Preterm birth | MIG | 0.4823 | 0.5629 | -0.0052 | 0.0209 | 0.8064 |  |  |  |  |
| Preterm birth | MIP1A | 0.4824 | 0.1387 | -0.0892 | 0.0444 | 0.1821 |  |  |  |  |
| Preterm birth | MIP1B | 0.1202 | 0.1531 | 0.0002 | 0.0201 | 0.9931 |  |  |  |  |
| Preterm birth | PDGFbb | 0.5014 | 0.5418 | -0.0130 | 0.0183 | 0.4912 |  |  |  |  |
| Preterm birth | RANTES | 0.0718 | 0.1034 | 0.0103 | 0.0340 | 0.7687 |  |  |  |  |
| Preterm birth | SCF | 0.2849 | 0.3689 | -0.0050 | 0.0243 | 0.8414 |  |  |  |  |
| Preterm birth | SCGFb | 0.6974 | 0.7532 | -0.0011 | 0.0148 | 0.9390 |  |  |  |  |
| Preterm birth | SDF1A | 0.0230 | 0.0202 | 0.0265 | 0.0293 | 0.3963 |  |  |  |  |
| Preterm birth | TNFα | 0.4257 | 0.6145 | -0.0092 | 0.0301 | 0.7878 |  |  |  |  |
| Preterm birth | TNFβ | 0.0334 | 0.0687 | 0.0010 | 0.0440 | 0.9838 |  |  |  |  |
| Preterm birth | TRAIL | 0.3211 | 0.1341 | -0.0250 | 0.0118 | 0.0542 |  |  |  |  |
| Preterm birth | VEGF | 0.5461 | 0.6121 | -0.0040 | 0.0162 | 0.8065 |  |  |  |  |

Abbreviations: se: standard error; Q-Pval-MR Egger: *P-*value of Cochran Q statistics test from MR Egger method; Q-Pval-IVW: *P-*value of Cochran Q statistics test from the inverse variance weighted method; P-value-MR Egger intercept: *P-*value of MR Egger intercept; CHP, chronic hypertension combined with pregnancy; GDM, gestational diabetes mellitus; NA: For the SNPs were less than 3, no further analysis was conducted; bNGF, beta nerve growth factor; CTACK, cutaneous T cell-attracting chemokine; FGF-Basic, basic fibroblast growth factor; GCSF, granulocyte colony-stimulating factor; GROA, growth-regulated oncogene-A; HGF, hepatocyte growth factor; IFNg, interferon gamma; IL, interleukin; IP, interferon gamma-induced protein 10; MCP1, monocyte chemotactic protein 1; MCP3, monocyte-specific chemokine 3; MCSF, macrophage colony-stimulating factor; MIF, macrophage migration inhibitory factor; MIG, monokine induced by interferon gamma; MIP1a, macrophage inflammatory protein-1a; MIP1b, macrophage inflammatory protein-1b; PDGFbb, platelet-derived growth factor BB; RANTES, regulated upon activation normal T cell expressed and secreted factor; SCF, stem cell factor; SCGFβ, stem cell growth factor beta; SDF1α, stromal cellderived factor-1 alpha; TNFα, tumor necrosis factor alpha; TNFβ, tumor necrosis factor beta; TRAIL, TNF-related apoptosis-inducing ligand; VEGF, vascular endothelial growth factor.

1. **Supplementary Table 3. SNPs information of inflammation cytokines with placental syndromes**

| exposure | outcome | inflammatory cytokines(exposure) | | | | | | placental syndromes(outcome) | | | |
| --- | --- | --- | --- | --- | --- | --- | --- | --- | --- | --- | --- |
|  |  | SNPs | effect allele | other allele | beta | se | P-value | se | P-value | R2 | F |
| GROA | pregnancy hypertension | rs114991247 | T | C | -0.2202 | 0.0463 | 0.0000 | 0.0293 | 0.0063 | 0.0063 | 22.6062 |
|  |  | rs115214168 | T | C | 0.4528 | 0.0828 | 0.0000 | 0.0598 | 0.9584 | 0.0084 | 29.8887 |
|  |  | rs1361829 | A | G | -0.1106 | 0.0241 | 0.0000 | 0.0176 | 0.5925 | 0.0059 | 21.0490 |
|  |  | rs140734053 | A | G | 0.7333 | 0.1545 | 0.0000 | 0.0955 | 0.8966 | 0.0063 | 22.5144 |
|  |  | rs150194856 | T | C | -0.4223 | 0.0914 | 0.0000 | 0.0669 | 0.6894 | 0.0060 | 21.3356 |
|  |  | rs17171245 | T | G | 0.2446 | 0.0530 | 0.0000 | 0.0411 | 0.7584 | 0.0060 | 21.2871 |
|  |  | rs185768063 | A | G | 0.4038 | 0.0760 | 0.0000 | 0.0544 | 0.9014 | 0.0079 | 28.2137 |
|  |  | rs188345231 | T | C | 0.6177 | 0.1322 | 0.0000 | 0.1038 | 0.8983 | 0.0061 | 21.8196 |
|  |  | rs3026943 | A | C | -0.1246 | 0.0256 | 0.0000 | 0.0186 | 0.5965 | 0.0066 | 23.6761 |
|  |  | rs62024303 | A | G | -0.3013 | 0.0660 | 0.0000 | 0.0436 | 0.4020 | 0.0058 | 20.8288 |
|  |  | rs76215157 | C | G | -0.7398 | 0.1564 | 0.0000 | 0.0894 | 0.5908 | 0.0063 | 22.3620 |
|  |  | rs76390238 | C | G | 0.6223 | 0.1352 | 0.0000 | 0.0920 | 0.8960 | 0.0059 | 21.1739 |
|  |  | rs79454658 | T | C | 0.2784 | 0.0596 | 0.0000 | 0.0428 | 0.0221 | 0.0061 | 21.8072 |
| IL-9 | pregnancy hypertension | rs117807175 | C | G | -0.5225 | 0.1106 | 0.0000 | 0.0784 | 0.9525 | 0.0060 | 22.3062 |
|  |  | rs1259728 | A | G | -0.2381 | 0.0507 | 0.0000 | 0.0382 | 0.0532 | 0.0060 | 22.0428 |
|  |  | rs3736858 | C | G | -0.1351 | 0.0291 | 0.0000 | 0.0217 | 0.2685 | 0.0058 | 21.5421 |
|  |  | rs41294750 | T | C | 0.3442 | 0.0736 | 0.0000 | 0.0525 | 0.0020 | 0.0059 | 21.8590 |
|  |  | rs4880409 | T | C | -0.3552 | 0.0716 | 0.0000 | 0.0879 | 0.0298 | 0.0067 | 24.5971 |
|  |  | rs73443903 | A | C | 0.2162 | 0.0460 | 0.0000 | 0.0349 | 0.4547 | 0.0060 | 22.0780 |
| HGF | preeclampsia | rs11060254 | A | G | -0.0765 | 0.0166 | 0.0000 | 0.0001 | 0.0200 | 0.0025 | 21.2326 |
|  |  | rs11129909 | T | C | -0.0738 | 0.0161 | 0.0000 | 0.0001 | 0.8600 | 0.0025 | 21.0066 |
|  |  | rs13412535 | A | G | -0.1043 | 0.0213 | 0.0000 | 0.0002 | 0.0087 | 0.0029 | 23.9721 |
| IL-10 | preeclampsia | rs10457128 | A | G | -0.0854 | 0.0172 | 0.0000 | 0.0001 | 0.2100 | 0.0032 | 24.6460 |
|  |  | rs10493718 | A | C | -0.1081 | 0.0222 | 0.0000 | 0.0002 | 0.3700 | 0.0031 | 23.7046 |
|  |  | rs13412535 | A | G | -0.1347 | 0.0224 | 0.0000 | 0.0002 | 0.0087 | 0.0047 | 36.1515 |
|  |  | rs1530455 | T | C | 0.0820 | 0.0174 | 0.0000 | 0.0001 | 0.5300 | 0.0029 | 22.2033 |
|  |  | rs2086656 | T | C | -0.0800 | 0.0170 | 0.0000 | 0.0001 | 0.9900 | 0.0029 | 22.1396 |
|  |  | rs3025021 | T | C | 0.0913 | 0.0194 | 0.0000 | 0.0001 | 0.8300 | 0.0029 | 22.1424 |
|  |  | rs339203 | T | C | 0.0954 | 0.0203 | 0.0000 | 0.0002 | 0.8500 | 0.0039 | 22.0776 |
|  |  | rs4741748 | A | G | -0.0788 | 0.0169 | 0.0000 | 0.0001 | 0.8700 | 0.0028 | 21.7353 |
|  |  | rs6054847 | T | C | 0.0971 | 0.0207 | 0.0000 | 0.0002 | 0.9100 | 0.0028 | 21.9981 |
|  |  | rs7088799 | T | G | -0.0815 | 0.0166 | 0.0000 | 0.0001 | 0.9000 | 0.0031 | 24.0983 |
|  |  | rs73192842 | A | G | 0.0949 | 0.0206 | 0.0000 | 0.0002 | 0.8600 | 0.0027 | 21.2171 |
|  |  | rs7747448 | A | G | -0.1061 | 0.0189 | 0.0000 | 0.0001 | 0.3400 | 0.0041 | 31.5061 |
|  |  | rs9472173 | T | C | -0.2004 | 0.0174 | 0.0000 | 0.0001 | 0.7100 | 0.0169 | 132.6125 |
| IL-10 | CHP | rs10457128 | A | G | -0.0854 | 0.0172 | 0.0000 | 0.0451 | 0.8529 | 0.0032 | 24.6460 |
|  |  | rs10493718 | A | C | -0.1081 | 0.0222 | 0.0000 | 0.0598 | 0.9790 | 0.0031 | 23.7046 |
|  |  | rs13412535 | A | G | -0.1347 | 0.0224 | 0.0000 | 0.0548 | 0.5468 | 0.0047 | 36.1515 |
|  |  | rs1530455 | T | C | 0.0820 | 0.0174 | 0.0000 | 0.0453 | 0.1241 | 0.0029 | 22.2033 |
|  |  | rs2086656 | T | C | -0.0800 | 0.0170 | 0.0000 | 0.0456 | 0.1818 | 0.0029 | 22.1396 |
|  |  | rs3002131 | C | G | 0.1191 | 0.0260 | 0.0000 | 0.0641 | 0.7591 | 0.0027 | 20.9780 |
|  |  | rs3025021 | T | C | 0.0913 | 0.0194 | 0.0000 | 0.0460 | 0.8808 | 0.0029 | 22.1424 |
|  |  | rs383684 | A | G | 0.0920 | 0.0197 | 0.0000 | 0.0770 | 0.4901 | 0.0028 | 21.8037 |
|  |  | rs4741748 | A | G | -0.0788 | 0.0169 | 0.0000 | 0.0445 | 0.2255 | 0.0028 | 21.7353 |
|  |  | rs6054847 | T | C | 0.0971 | 0.0207 | 0.0000 | 0.0550 | 0.1053 | 0.0028 | 21.9981 |
|  |  | rs6680918 | T | C | -0.1202 | 0.0250 | 0.0000 | 0.0657 | 0.9540 | 0.0030 | 23.1109 |
|  |  | rs7088799 | T | G | -0.0815 | 0.0166 | 0.0000 | 0.0448 | 0.2599 | 0.0031 | 24.0983 |
|  |  | rs73192842 | A | G | 0.0949 | 0.0206 | 0.0000 | 0.0553 | 0.3798 | 0.0027 | 21.2171 |
|  |  | rs7747448 | A | G | -0.1061 | 0.0189 | 0.0000 | 0.0498 | 0.3432 | 0.0041 | 31.5061 |
|  |  | rs9472173 | T | C | -0.2004 | 0.0174 | 0.0000 | 0.0444 | 0.0435 | 0.0169 | 132.6125 |
| IL-18 | CHP | rs10409850 | A | G | 0.1791 | 0.0347 | 0.0000 | 0.0647 | 0.1350 | 0.0072 | 26.6254 |
|  |  | rs11214093 | T | C | 0.1143 | 0.0238 | 0.0000 | 0.0436 | 0.3763 | 0.0062 | 23.0517 |
|  |  | rs117266781 | T | C | 0.7051 | 0.1436 | 0.0000 | 0.2425 | 0.0028 | 0.0065 | 24.0966 |
|  |  | rs117371668 | T | G | 0.3712 | 0.0799 | 0.0000 | 0.1485 | 0.5723 | 0.0058 | 21.5718 |
|  |  | rs139468359 | T | C | 0.5101 | 0.1088 | 0.0000 | 0.1957 | 0.0247 | 0.0059 | 21.9693 |
|  |  | rs1979967 | T | C | 0.1400 | 0.0285 | 0.0000 | 0.0528 | 0.0277 | 0.0065 | 24.1174 |
|  |  | rs4952239 | A | T | -0.1156 | 0.0242 | 0.0000 | 0.0449 | 0.2352 | 0.0062 | 22.8060 |
|  |  | rs58701153 | A | T | -0.1265 | 0.0242 | 0.0000 | 0.0454 | 0.9799 | 0.0074 | 27.3095 |
|  |  | rs62312914 | T | C | -0.1265 | 0.0250 | 0.0000 | 0.0449 | 0.2313 | 0.0069 | 25.5897 |
|  |  | rs764078 | A | T | 0.1283 | 0.0278 | 0.0000 | 0.0515 | 0.4925 | 0.0058 | 21.2876 |
|  |  | rs77187209 | T | C | -0.4859 | 0.1041 | 0.0000 | 0.1806 | 0.1916 | 0.0059 | 21.7749 |
|  |  | rs78623212 | T | C | 0.8322 | 0.1676 | 0.0000 | 0.2575 | 0.5926 | 0.0067 | 24.6417 |
|  |  | rs78716465 | A | G | 0.3173 | 0.0679 | 0.0000 | 0.1193 | 0.7110 | 0.0059 | 21.8255 |
| MCSF | CHP | rs116274860 | T | G | 0.8262 | 0.1739 | 0.0000 | 0.1943 | 0.0217 | 0.0090 | 22.5540 |
|  |  | rs116887628 | A | G | -0.2741 | 0.0598 | 0.0000 | 0.0850 | 0.9880 | 0.0083 | 20.9927 |
|  |  | rs117867915 | T | C | 0.5224 | 0.1096 | 0.0000 | 0.1540 | 0.4565 | 0.0090 | 22.7006 |
|  |  | rs11963606 | C | G | -0.5353 | 0.1170 | 0.0000 | 0.1777 | 0.4851 | 0.0083 | 20.9158 |
|  |  | rs12962919 | T | C | 0.3025 | 0.0659 | 0.0000 | 0.0862 | 0.2886 | 0.0084 | 21.0539 |
|  |  | rs139457375 | A | C | -0.4047 | 0.0854 | 0.0000 | 0.1240 | 0.5289 | 0.0089 | 22.4390 |
|  |  | rs147378920 | A | G | -0.6064 | 0.1318 | 0.0000 | 0.1491 | 0.8932 | 0.0084 | 21.1514 |
|  |  | rs34089869 | T | C | 0.2194 | 0.0462 | 0.0000 | 0.0698 | 0.7610 | 0.0089 | 22.5342 |
|  |  | rs62294910 | A | G | 0.3472 | 0.0687 | 0.0000 | 0.0906 | 0.3511 | 0.0101 | 25.5210 |
|  |  | rs72723242 | T | G | -0.4969 | 0.1083 | 0.0000 | 0.1729 | 0.0520 | 0.0084 | 21.0346 |
|  |  | rs9387100 | T | C | -0.1350 | 0.0290 | 0.0000 | 0.0437 | 0.3006 | 0.0086 | 21.6533 |
|  |  | rs9626985 | T | C | 0.2277 | 0.0496 | 0.0000 | 0.0745 | 0.3347 | 0.0084 | 21.0579 |
| PDGFbb | CHP | rs10512952 | T | C | -0.2816 | 0.0587 | 0.0000 | 0.1646 | 0.6161 | 0.0028 | 23.0083 |
|  |  | rs116154010 | T | C | 0.3225 | 0.0662 | 0.0000 | 0.1823 | 0.5691 | 0.0028 | 23.7268 |
|  |  | rs11766649 | A | G | 0.0902 | 0.0196 | 0.0000 | 0.0538 | 0.7541 | 0.0025 | 21.1737 |
|  |  | rs12289510 | A | G | -0.0772 | 0.0158 | 0.0000 | 0.0434 | 0.0527 | 0.0029 | 23.8680 |
|  |  | rs12615784 | T | C | -0.1003 | 0.0193 | 0.0000 | 0.0529 | 0.4814 | 0.0032 | 27.0012 |
|  |  | rs147862316 | T | C | 0.2279 | 0.0411 | 0.0000 | 0.1146 | 0.8437 | 0.0037 | 30.7398 |
|  |  | rs2643354 | A | G | 0.1251 | 0.0261 | 0.0000 | 0.0722 | 0.5339 | 0.0027 | 22.9683 |
|  |  | rs35859699 | A | G | -0.3854 | 0.0838 | 0.0000 | 0.2127 | 0.3318 | 0.0025 | 21.1461 |
|  |  | rs62191444 | T | G | -0.1120 | 0.0239 | 0.0000 | 0.0608 | 0.3559 | 0.0026 | 21.9551 |
|  |  | rs6756793 | T | C | 0.0876 | 0.0157 | 0.0000 | 0.0437 | 0.2858 | 0.0037 | 31.1247 |
|  |  | rs6910518 | T | G | 0.0806 | 0.0162 | 0.0000 | 0.0443 | 0.1118 | 0.0030 | 24.7478 |
|  |  | rs72972467 | C | G | -0.1616 | 0.0328 | 0.0000 | 0.0865 | 0.8098 | 0.0029 | 24.2678 |
|  |  | rs73162807 | A | C | -0.2313 | 0.0499 | 0.0000 | 0.1417 | 0.0054 | 0.0026 | 21.4806 |
|  |  | rs9924851 | C | G | 0.0767 | 0.0163 | 0.0000 | 0.0452 | 0.7790 | 0.0026 | 22.1366 |
| MIF | GDM | rs1007888 | T | C | -0.1275 | 0.0245 | 0.0000 | 0.0206 | 0.4673 | 0.0076 | 27.0671 |
|  |  | rs113218956 | A | G | -0.8789 | 0.1876 | 0.0000 | 0.1625 | 0.1519 | 0.0062 | 21.9365 |
|  |  | rs11551183 | C | G | 0.3666 | 0.0795 | 0.0000 | 0.0693 | 0.0779 | 0.0060 | 21.2522 |
|  |  | rs12594190 | A | G | 0.1321 | 0.0266 | 0.0000 | 0.0219 | 0.7050 | 0.0069 | 24.6488 |
|  |  | rs141009259 | T | C | -0.6194 | 0.1285 | 0.0000 | 0.0951 | 0.0495 | 0.0065 | 23.2215 |
|  |  | rs2294689 | C | G | -0.1338 | 0.0287 | 0.0000 | 0.0835 | 0.3233 | 0.0061 | 21.7221 |
|  |  | rs35792361 | A | G | -0.2586 | 0.0527 | 0.0000 | 0.0429 | 0.0287 | 0.0068 | 24.0652 |
|  |  | rs35890933 | T | G | 0.1676 | 0.0365 | 0.0000 | 0.0285 | 0.1736 | 0.0059 | 21.0725 |
|  |  | rs3814097 | A | G | -0.1163 | 0.0251 | 0.0000 | 0.0204 | 0.5217 | 0.0060 | 21.4569 |
|  |  | rs78098071 | T | C | -0.4583 | 0.0915 | 0.0000 | 0.0791 | 0.1327 | 0.0071 | 25.0733 |
| IL-1β | pregnancy loss | rs143319329 | T | C | 0.4357 | 0.0930 | 0.0000 | 0.0576 | 0.7123 | 0.0065 | 21.9356 |
|  |  | rs4786740 | A | C | 0.1264 | 0.0265 | 0.0000 | 0.0096 | 0.0430 | 0.0067 | 22.7375 |
|  |  | rs61335305 | A | C | 0.4333 | 0.0928 | 0.0000 | 0.0346 | 0.0660 | 0.0065 | 21.7882 |
| TRAIL | preterm birth | rs113057689 | A | G | -0.2625 | 0.0489 | 0.0000 | 0.0479 | 0.0191 | 0.0035 | 28.8095 |
|  |  | rs11875481 | T | C | -0.0969 | 0.0211 | 0.0000 | 0.0268 | 0.7932 | 0.0026 | 21.0852 |
|  |  | rs12458564 | A | T | -0.1002 | 0.0175 | 0.0000 | 0.0221 | 0.8558 | 0.0040 | 32.7758 |
|  |  | rs13278062 | T | G | 0.0800 | 0.0157 | 0.0000 | 0.0200 | 0.4327 | 0.0031 | 25.9582 |
|  |  | rs139958028 | A | G | 0.1803 | 0.0395 | 0.0000 | 0.0478 | 0.7811 | 0.0025 | 20.8301 |
|  |  | rs183815186 | A | T | -0.3499 | 0.0602 | 0.0000 | 0.0730 | 0.3215 | 0.0041 | 33.7745 |
|  |  | rs550057 | T | C | -0.0783 | 0.0169 | 0.0000 | 0.0217 | 0.0137 | 0.0026 | 21.4607 |
|  |  | rs558572 | T | C | 0.1351 | 0.0265 | 0.0000 | 0.0341 | 0.0301 | 0.0031 | 25.9844 |
|  |  | rs57396456 | T | C | -0.5641 | 0.0516 | 0.0000 | 0.0671 | 0.5014 | 0.0143 | 119.4833 |
|  |  | rs616114 | T | C | -0.1033 | 0.0162 | 0.0000 | 0.0206 | 0.0360 | 0.0049 | 40.6504 |
|  |  | rs62093482 | T | C | 0.9827 | 0.0529 | 0.0000 | 0.0695 | 0.9339 | 0.0402 | 345.0047 |
|  |  | rs747324 | T | C | -0.0826 | 0.0178 | 0.0000 | 0.0222 | 0.6162 | 0.0026 | 21.5285 |
|  |  | rs75928541 | A | G | 0.2784 | 0.0591 | 0.0000 | 0.0682 | 0.5071 | 0.0027 | 22.1849 |
|  |  | rs7599203 | T | C | 0.0918 | 0.0200 | 0.0000 | 0.0254 | 0.0671 | 0.0026 | 21.0630 |
|  |  | rs78682108 | A | G | -0.2383 | 0.0394 | 0.0000 | 0.0459 | 0.9672 | 0.0044 | 36.5721 |
|  |  |  |  |  |  |  |  |  |  |  |  |

Abbreviations: se: standard error; SNPs, single nucleotide polymorphisms; CHP, chronic hypertension combined with pregnancy; GDM, gestational diabetes mellitus.GROA, growth-regulated oncogene-A; IL, interleukin; HGF, hepatocyte growth factor; MCSF, macrophage colony-stimulating factor; PDGFbb, platelet-derived growth factor BB; MIF, macrophage migration inhibitory factor; TRAIL, TNF-related apoptosis-inducing ligand.


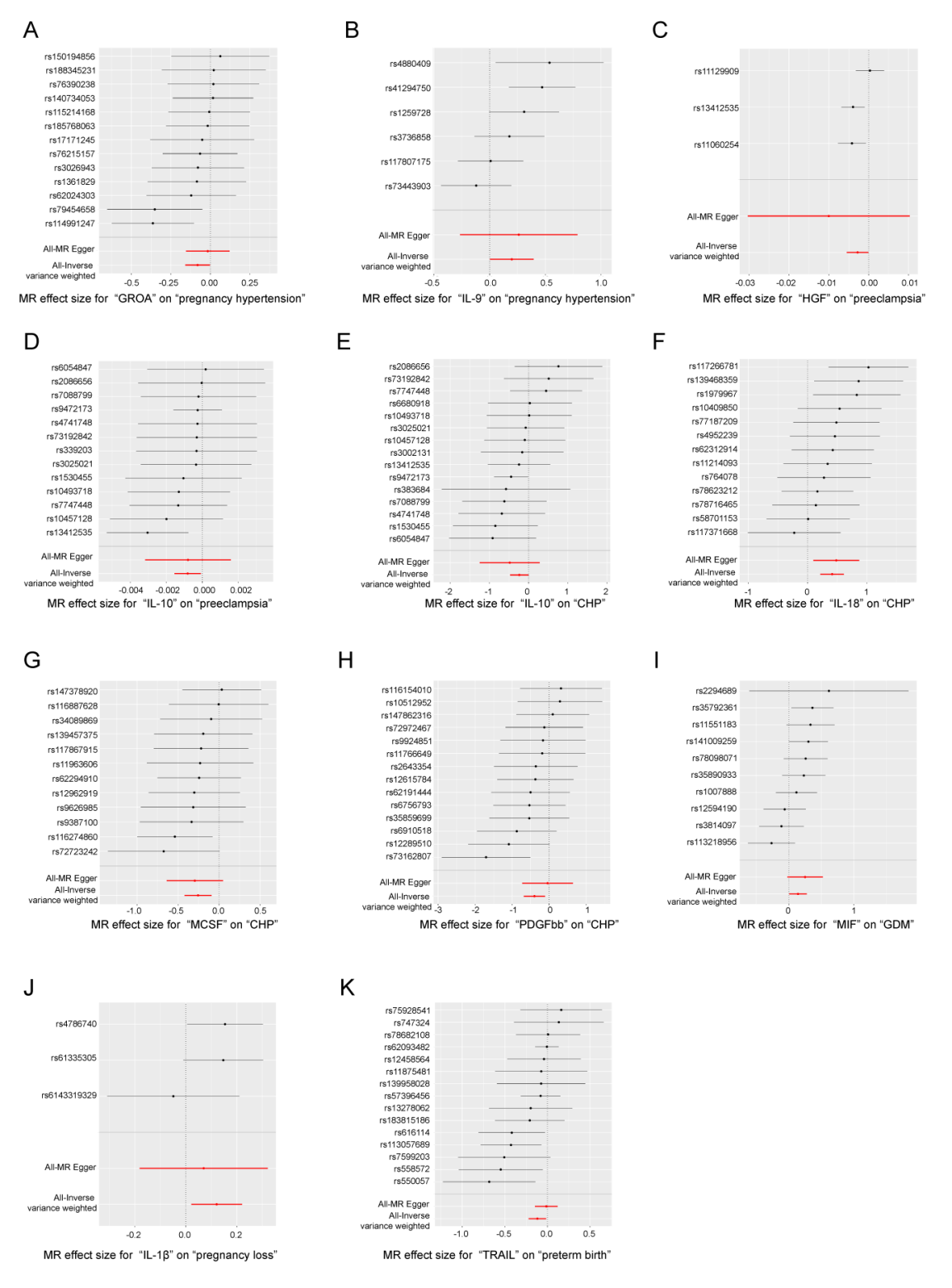


**Supplementary Figure 1. Forest plots of mendelian randomization analyses for inflammatory cytokines on placental syndromes.** A and B. GROA and IL-9 on pregnancy hypertension. C and D. HGF and IL-10 on preeclampsia. E,F,G and H. IL-10, IL-18, MCSF and PDGFbb on CHP. I. MIF on GDM. J. IL-1β on pregnancy loss. K. TRAIL on preterm birth. Abbreviations: GROA, growth-regulated oncogene-A; IL, interleukin; HGF, hepatocyte growth factor; MCSF, macrophage colony-stimulating factor; PDGFbb, platelet-derived growth factor BB; CHP, chronic hypertension combined with pregnancy; MIF, macrophage migration inhibitory factor; GDM, gestational diabetes mellitus; TRAIL, TNF-related apoptosis-inducing ligand.


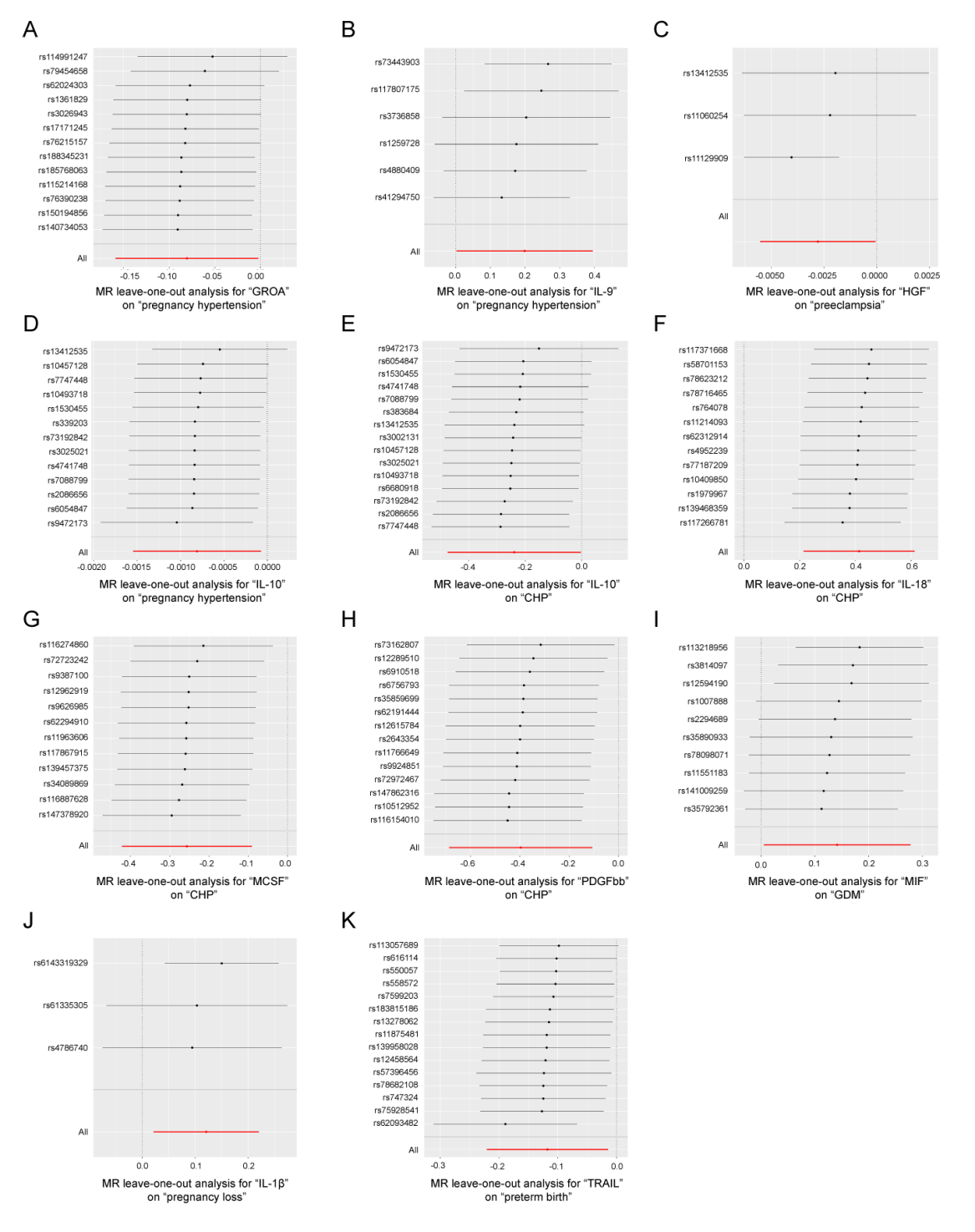


**Supplementary Figure 2. Leave-one-out sensitivity analyses of mendelian randomization analyses between inflammatory cytokines and placental syndromes.** A and B. GROA and IL-9 on pregnancy hypertension. C and D. HGF and IL-10 on preeclampsia. E,F,G and H. IL-10, IL-18, MCSF and PDGFbb on CHP. I. MIF on GDM. J. IL-1β on pregnancy loss. K. TRAIL on preterm birth. Abbreviations: GROA, growth-regulated oncogene-A; IL, interleukin; HGF, hepatocyte growth factor; MCSF, macrophage colony-stimulating factor; PDGFbb, platelet-derived growth factor BB; CHP, chronic hypertension combined with pregnancy; MIF, macrophage migration inhibitory factor; GDM, gestational diabetes mellitus; TRAIL, TNF-related apoptosis-inducing ligand.
